# -Omics biomarker identification pipeline for translational medicine

**DOI:** 10.1186/s12967-019-1912-5

**Published:** 2019-05-14

**Authors:** Laura Bravo-Merodio, John A. Williams, Georgios V. Gkoutos, Animesh Acharjee

**Affiliations:** 1 College of Medical and Dental Sciences, Institute of Cancer and Genomic Sciences, Centre for Computational Biology, University of Birmingham, Birmingham, B15 2TT UK; 2 Institute of Translational Medicine, University Hospitals Birmingham NHS Foundation Trust, Birmingham, B15 2TT UK; 3 Mammalian Genetics Unit, Medical Research Council Harwell Institute, Harwell Campus, Didcot, OX11 0RD UK; 4 MRC Health Data Research UK (HDR UK), London, UK; 5 NIHR Experimental Cancer Medicine Centre, Birmingham, B15 2TT UK; 6 NIHR Surgical Reconstruction and Microbiology Research Centre, Birmingham, B15 2TT UK; 7 NIHR Biomedical Research Centre, Birmingham, B15 2TT UK

**Keywords:** Biomarker, -Omics, Regularization, Feature selection, Translational medicine

## Abstract

**Background:**

Translational medicine (TM) is an emerging domain that aims to facilitate medical or biological advances efficiently from the scientist to the clinician. Central to the TM vision is to narrow the gap between basic science and applied science in terms of time, cost and early diagnosis of the disease state. Biomarker identification is one of the main challenges within TM. The identification of disease biomarkers from -omics data will not only help the stratification of diverse patient cohorts but will also provide early diagnostic information which could improve patient management and potentially prevent adverse outcomes. However, biomarker identification needs to be robust and reproducible. Hence a robust unbiased computational framework that can help clinicians identify those biomarkers is necessary.

**Methods:**

We developed a pipeline (workflow) that includes two different supervised classification techniques based on regularization methods to identify biomarkers from -omics or other high dimension clinical datasets. The pipeline includes several important steps such as quality control and stability of selected biomarkers. The process takes input files (outcome and independent variables or -omics data) and pre-processes (normalization, missing values) them. After a random division of samples into training and test sets, Least Absolute Shrinkage and Selection Operator and Elastic Net feature selection methods are applied to identify the most important features representing potential biomarker candidates. The penalization parameters are optimised using 10-fold cross validation and the process undergoes 100 iterations and a combinatorial analysis to select the best performing multivariate model. An empirical unbiased assessment of their quality as biomarkers for clinical use is performed through a Receiver Operating Characteristic curve and its Area Under the Curve analysis on both permuted and real data for 1000 different randomized training and test sets. We validated this pipeline against previously published biomarkers.

**Results:**

We applied this pipeline to three different datasets with previously published biomarkers: lipidomics data by Acharjee et al. (Metabolomics 13:25, 2017) and transcriptomics data by Rajamani and Bhasin (Genome Med 8:38, 2016) and Mills et al. (Blood 114:1063–1072, 2009). Our results demonstrate that our method was able to identify both previously published biomarkers as well as new variables that add value to the published results.

**Conclusions:**

We developed a robust pipeline to identify clinically relevant biomarkers that can be applied to different -omics datasets. Such identification reveals potentially novel drug targets and can be used as a part of a machine-learning based patient stratification framework in the translational medicine settings.

**Electronic supplementary material:**

The online version of this article (10.1186/s12967-019-1912-5) contains supplementary material, which is available to authorized users.

## Background

Translational medicine (TM) [1–3] is an emerging and fast growing area of research that aims to facilitate medical or biological advances efficiently from the scientist to the clinician. TM approaches uses diagnostic tools and treatments by commonly employing interdisciplinary frameworks, in a highly collaborative manner to reach out to the patient community for disease treatment, stratification and prevention. A notion central to TM is to narrow the gap between basic science and applied science in terms of time, cost and early diagnosis of the disease state. Over the last few decades the influx of untargeted -omics (phenomics, transcriptomics, metabolomics, epigenomics, lipidomics and others) datasets have enabled the identification of biological markers of disease (so-called biomarkers) [4], and have become one of the main avenues towards discovery within TM. The identification of the disease biomarkers from -omics data does not only facilitate the stratification of patient cohorts but also provides early diagnostic information to improve patient management and prevent adverse outcomes. However, biomarker identification, a task that is commonly comprised of biological and computational processes, needs to be robust and reproducible if it is to be clinically useful and actionable in patient-care settings or in response to new therapies. Therefore, a robust unbiased computational framework is necessary to identify biological signals that can reveal potential novel biomarkers.

In -omics literature, there has been a recent trend towards the identification of data pre- and post-processing steps. For example, Satagopam et al. [5] developed an infrastructure comprised by a combination of web services, tranSMART, Galaxy, and MINERVA platforms. Narayanasam et al. [6] developed an integrated reference-independent analysis of metagenomic and metatranscriptomic data for the analysis of microbiome derived datasets. Feng [7] developed a proteomics pipeline called Firmiana. Firmiana is a cloud platform that allows scientists to deposit mass spectrometry (MS) raw files online and performs automated bioinformatic analyses on the uploaded data. Such existing robust pipelines for analysing -omics data are often either focused on specific -omics data or can be used only for either classification or regression purposes. For example, Xia et al. [8] developed a workflow for quantitative metabolomics datasets, Acharjee et al. [9] developed an -omics fusion tool but focused on metabolomics data in regression mode only. Hermida et al. [10] developed a pipeline based on transcriptomics data called Confero that extracts gene lists from research papers and performs automatic extraction and storage of gene sets. While this is useful for downstream analysis, there is a need to combine these approaches and deal with multiple types of outcome data as well as consider their categorical or continuous nature. In some cases, the complexity of machine learning models associated visualizations used hinder the interpretability of the results and therefore impair their translation into clinical science.

In this study, we develop a pipeline that includes two machine learning algorithms, inspired by simple linear models, coupled with follow-up approaches for systematic data analysis. Our systematic analysis includes data quality checks, identification of important features, as well as combinatorial and stability analyses. We applied and validated our pipeline with three different previously published -omics datasets. Our approach successfully identified the markers reported in the literature as well as potential novel markers.

## Materials and methods

Our pipeline is composed of statistical machine learning modules whose methods are described below. Additionally, we applied and validated our pipeline against three independent published datasets; two RNA microarray datasets and one lipidomics experiment.

### Machine learning methods

We used two feature selection methods, LASSO [11] and Elastic Net [12]. These are two forms of regularization methods that are able to automatically select the features from the dataset and hence provide a sparse solution. Regularization works in the following way: Starting from simple linear regression models we consider $x_{1} \ldots x_{p}$ as *x* number of predictor variables (features) and $y$ as an outcome or response variable:

1 $$\hat{y} = \hat{\beta }_{0} + \hat{\beta }_{1} x_{1} + \hat{\beta }_{2} x_{2} + \cdots \hat{\beta }_{p} x_{p}$$

Here, the outcome of model fitting produces the vector of estimated regression coefficients through ordinary least squares (OLS), with the objective function as the minimum of the residual sum of the squares (RSS) equation (Eq. 2). The values minimizing the function are the estimated regression coefficients (β).

2 $$Residual sum of squares\left( {RSS} \right) = \mathop \sum \limits_{i = 1}^{N} \left( {y_{i} - x_{i}^{T} \beta } \right)^{2}$$

In regularization methods, an extra term is added (Eq. 3) and so the new objective function to minimize becomes:

3 $$RSS\left( \beta \right) + p\lambda \left( \beta \right)$$

Here $p$ is a function to penalize and $\lambda$ forms the penalty/regularization parameter. The penalty function $\lambda$ controls the trade-off between likelihood and penalty and so influences the variables to be selected. The higher the value of $\lambda$, the fewer number of variables are selected and vice versa. The differences between regularization methods lie in the different functions $p$ they penalize. In LASSO, the penalty is applied to the sum of the absolute values of the regression coefficients (L1 norm). Mathematically, we can write this as:

4 $$\frac{minimize}{{\beta \in R^{p} }}\frac{1}{2}\left\| {y - X\beta } \right\|_{2}^{2} + \lambda \left\| \beta \right\|_{1 }$$

The left part of the equation is the normal least squares criterion, whereas the right part is the penalized sum of the absolute values of the regression coefficients.

In Ridge regression [13], the precursor of LASSO, the penalization $p$ is incurred in the L2 norm of the coefficients (sum of the squares). In this case, selection is not sparse since coefficients are never zero but close and so, a rank of features based on the penalised regression coefficients, is produced. Elastic Net [12], on the other hand, is a mixed version of both LASSO and Ridge (Eq. 5). It allows for the sparse representation, similarly to LASSO, and theoretically improves its performance in $p \gg n$ cases with high collinear groups of features by allowing grouped selection or de selection of correlated variables. LASSO instead tends to select only one “random” variable from the group of pairwise correlations. EN is created through the merging of both Ridge and LASSO penalizations (Eq. 5). A different representation of the same equation can be seen below (Eq. 6), with a single parameter $\alpha$ regulating the relationship between Ridge and LASSO. When $\alpha$ is equal or closer to 0 we have a stronger penalization and so a solution closer or equal to LASSO whereas, when $\alpha$ is equal or closer to 1, the behaviour resembles Ridge.

5 $$\frac{minimize}{{\beta \in R^{p} }}\frac{1}{2}\left\| {y - X\beta } \right\|_{2}^{2} + \lambda_{1} \left\| \beta \right\|_{1} + \lambda_{2} \left\| \beta \right\|_{2}^{2}$$

6 $$\frac{minimize}{{\beta \in R^{p} }}\frac{1}{2}\left\| {y - X\beta } \right\|_{2}^{2} \;subject\;\left( {1 - \alpha } \right)\left\| \beta \right\|_{1} + \alpha \left\| \beta \right\|^{2}$$

This combination of LASSO and EN methods comprise the backbone of our pipeline and the construction is described below.

### Pipeline construction and follow-up analysis methods

All analyses were performed in the R statistical computing (R version 3.4.3) environment [14]. All R packages can be found in our project’s github repository stated below. The necessary software dependencies are described in the README file located in the repository. All analyses can be performed on a standard PC environment with the run time increasing with larger datasets. For example, an analysed RNA microarray acute myeloid leukaemia (AML) dataset described below took 8 h to complete, but at no time did the R environment use more than 1 Gb of RAM. The workflow is embedded in a R Markdown file which, when altered with a user’s working and output directories and the name of the input data file, runs the analysis in real time. After running, it compiles a PDF report of containing both all code generated and figures produced. Figures generated include analogues to Figs. 2 and 3. Additionally, ROC AUC curves are generated via stability analyses for individual selected features as well as combinations shown to be significant in predicting binary outcome. Importantly, a list of significant features (genes, metabolites, etc.) is printed in the PDF report. These lists can easily be copied so as to be used as input for pathway and ontology enrichment analyses. For the purposes of our validation studies, pathway analysis and ontology enrichment wereperformed with the EnrichR tool with default settings (analysed on Sept 7, 2018) [15].

### Lipidomics data

To assess the performance of our framework, we employed a previously published lipidomics dataset from Acharjee et al. [16]. The lipidomics data was generated from The Cambridge Baby Growth Study (CBGS), a prospective observational birth cohort. For details about the processes related to the data generation as well as sample information please check Acharjee et al., and Prentice et al. [16, 17].

From the CBGS cohort we used 3 different datasets, namely CBGS-1, CBGS-2 and POPS (Pregnancy Outcome Prediction Study). All data was obtained from dried blood spots and generated with direct infusion high-resolution mass spectrometry (HRMS).

A summary of the cohort is listed below (Table 1).

**Table 1:** Cohort statistics of samples analysed form the Cambridge Baby Growth Study (CBGS) and Pregnancy Outcome Prediction Study (POPS)

Cohort name	Sample information			Total sample number (n)
	Formula milk (FM)	Breast milk(HM)	Mixed (FM and HM)	
CBGS-1	85	87	67	239
CBGS-2	43	25	27	95
POPS	16	11	13	40

### Transcriptomic data

A pancreatic ductal adenocarcinoma (PDAC) microarray expression dataset (n = 36 control, n = 36 cases) GSE15471, [18], as well as microarray expression data from a three-cohort study of acute myeloid leukaemia (AML) cell lines with n = 404 AML samples and n = 138 control samples, excluding a third transitional cohort of MDS samples GSE15061 [19] was analysed. In each case, the Robust Multichip Average standardized Affymetrix Human Genome U133 Plus 2.0 data submitted by the authors to the Gene Expression Omnibus was taken as inputalong with class information indicating case or control condition. Pre-processing of microarray data included; (a) taking the median of duplicate probes across all conditions to yield one unique probe per experiment, (b) collapsing rows of probes belonging to identical genes and taking maximum expressed probe via the WGCNA R package [20], and (c) testing features (samples) for low variance via the caret R package and removing those with near-zero variance among all genes [21]. The resulting numerical matrix of normalized gene expression values was used as input for each experiment, yielding 22,880 genes and 542 samples for the AML dataset, and an equal number of genes and 78 samples for the pancreatic cancer dataset. Normalized data matrices produced both for validating the reproducibility of our pipeline, as well as the ones used as example input, are available in our project github repository. The different steps comprising our pipeline are described in the results section.

### Availability of code

All code and functions are available on our hosted GitHub repository: https://github.com/jaw-bioinf/Biomarker_Identification/.

## Results

### Pipeline features

**Fig. 1:**
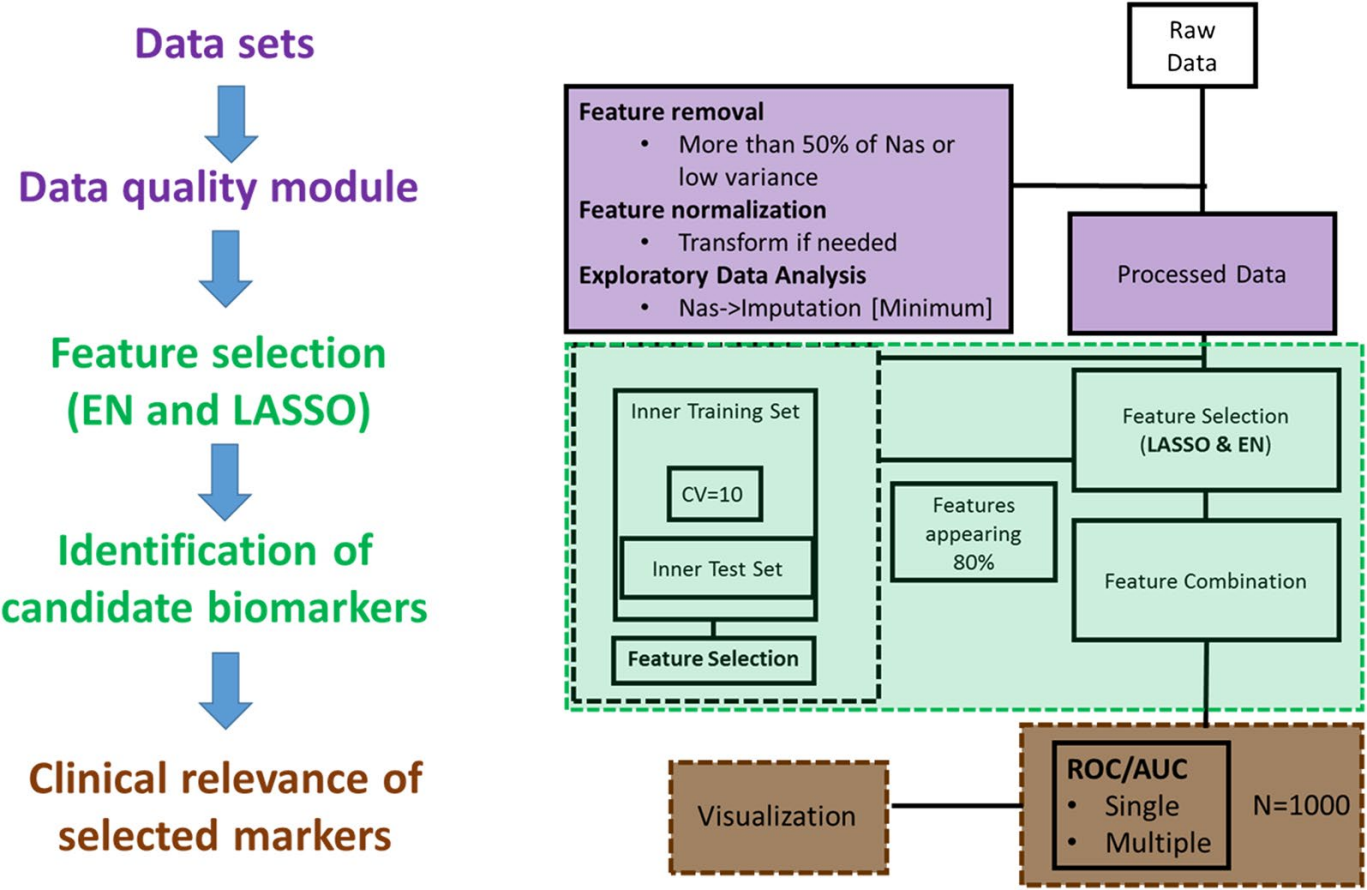
Structure of the pipeline and different steps are shown. This consist of the data quality checks (Purple), feature selection with optimised parameters (Green), identification of the biomarkers (Green), and finally accessing the clinical relevance of the markers based on the stability analysis and data visualization (Brown)

A graphical depiction of our workflow can be seen in Fig. 1. Our pipeline can be divided in the three modules that we describe below with the purple data quality module offering different options depending on the type of data introduced e.g. microarray and generic/other data.

### Data quality module

The data quality module consists of different checks on both features and samples to exclude or data based on the amount of missingness, and to standardize data to make measurements of features in different experiments comparable. Missing value imputation and normalisation steps can be implemented as needed by end users. Normalization methods can be bypassed if RNA microarray datasets are downloaded pre-processed directly from repositories. For these and other microarray datasets, optional filtering steps to reduce dimensionality include many-to-one probe-to-gene mapping. Features may be further reduced by using external tools or expert biological insight to exclude features, but such reduction is optional. In all cases, the input for the ‘feature selection’ segment of the pipeline is a matrix (file or data frame) of features (potential biomarkers) as well as a set of samples along with a target or outcome variable.

### Feature selection and parameter optimization

In order to apply the LASSO and EN algorithms for biologically relevant feature selection, we need to optimize the penalty parameter associated with each of the methods in an unbiased way. To achieve this, the pipeline divides samples randomly into a training set composed of 75% of the total number of the samples and a test set consisting of the remaining 25% samples. To ensure optimal training in real-world datasets, all data splits retained class balances of the target variable, so each split reflected a proportion of the target observed in the underlying dataset. We note that class balancing measures, such as boosting or under sampling, are not used to artificially balance training/testing data in each split (outer loop set). Then we apply a 10-fold cross validation on the training set (inner loop set) aiming to have an optimised penalty parameter that can be plugged into the LASSO and EN models. Mathematically, LASSO and EN models can be defined by using a single penalty function “$\alpha$” [22] (Eq. 6). For example, by using a penalty parameter $\alpha = 1$, we are applying the LASSO algorithm, whereas $\alpha = 0.5$ will perform Elastic Net. A high value for the penalty parameter ($\alpha$) will result in a strong penalty and hence fewer variables will be selected.

### Identification of candidate biomarkers

Our pipeline iterates model creation 100 times and selects the features that appear more than 90 times in the analysis, as these we deem to be the more significant for the classification model. Moreover, in order to better understand the relationship between the features selected and the outcome variable analysed, a display of the weight ($\beta$ coefficients) distribution per model (see Additional file 1) and a box plot of the class differences per feature is generated (Fig. 3b). These selected features are then considered potential candidate biomarkers. In order to ascertain their validity as biomarkers, their performance is evaluated both alone and in combination.

### Performance evaluation and visualization

In order to investigate the performance of the selected markers, our pipeline performs stability analysis through a permutation test. This consists of the randomization of the label features, resulting in incorrect sample labels for predictions and generating models with ROC AUC values showing a performance subject to the random distribution. Both the real model and permutation tests are produced by sampling 1000 random training and test sets, then using simple machine learning models to consider the fit of data, with ROC AUC performance results plotted as density plots alongside their means and standard deviation. The ROC AUC offers a graphical overview of the diagnostic ability of binary classifiers with varying thresholds. In addition to this, more information on the predictive ability of the model is obtained through the calculation of the sensitivity, specificity, precision and accuracy values.

### Validation of the approach

#### Lipidomics data

**Fig. 2:**
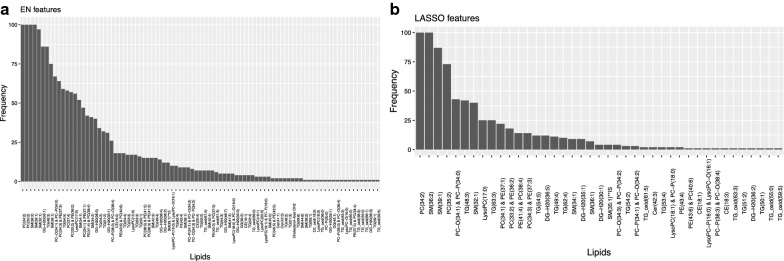
**a** The frequency of the lipids (y axis) ranked by their selection out of 100 randomized sampling splits using Elastic Net. **b** A similar analysis is shown using LASSO. Features identified in both analyses are used for further investigation

We applied our pipeline in the published lipidomics data available from three cohorts: the Cambridge Baby Growth Study (CBGS1and CBGS2) and the Pregnancy Outcome Prediction Study (POPS). Our objective was to identify potentially nutritional lipid biomarkers for the classification of babies fed with Formula, Human or a mix of Human and Formula milk. In Fig. 2a, we display the frequency of appearance of the lipids in 100 different Elastic Net models of classification between Formula and Human milk nutrition from CBGS2 data. Figure 2b shows the same results but for LASSO. It can be seen that EN allows for a less stringent solution with more features appearing. Additional file 2: Table 2 reveals the high-ranking lipids identified by our approach as well as their associated nutritional outcomes.

**Fig. 3:**
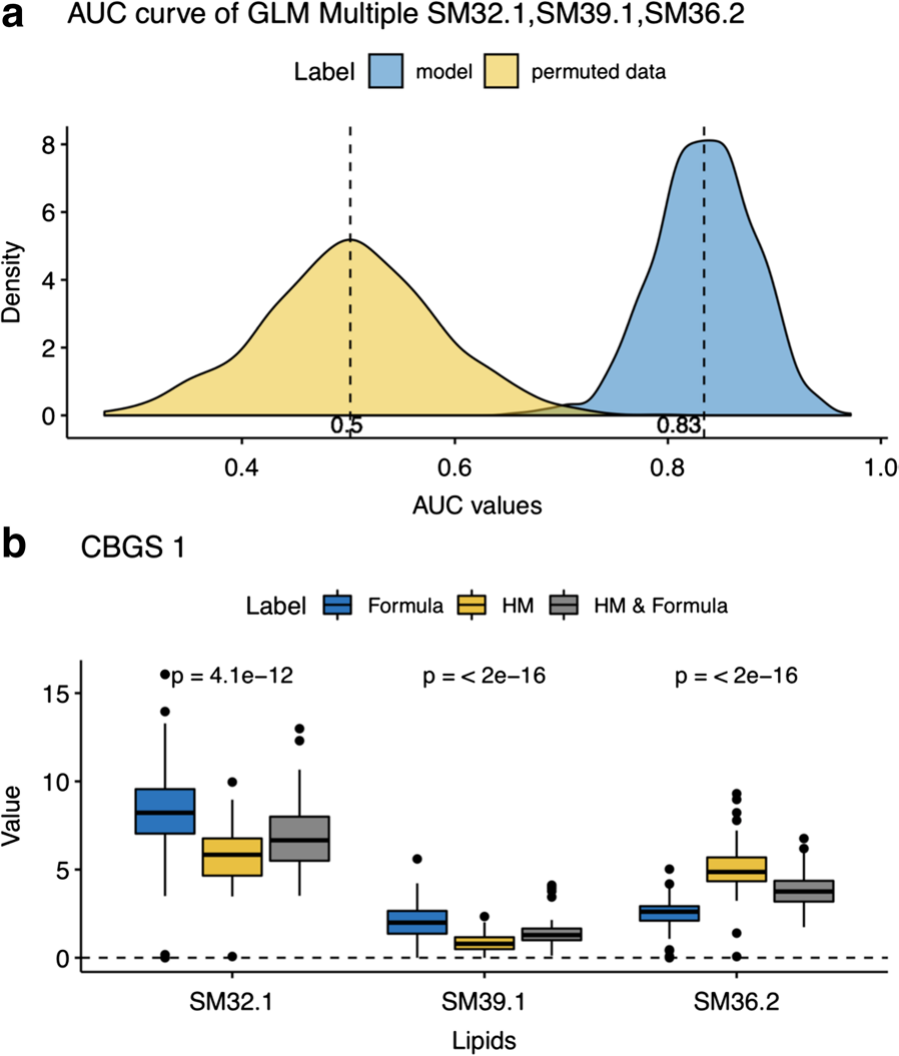
**a** Stability of combination of lipids SM(39:1), SM(32:1) and SM(36:2) (blue) versus random sampling-derived null distribution (yellow) in CBGS1, formula milk versus formula milk and human milk model prediction. X-axis is the area under the receiver operating characteristic curve (ROC), and dashed lines represent the mean over all 1000 sampled trials conducted. **b** Probability values showing a significant difference between the measured levels of selected lipids between all the nutritional classes (formula vs. human milk vs. mixed)

For those selected features, performance evaluation was then performed. Results can be seen in Fig. 3a where, a combination of the three selected lipids SM(39:1), SM(32.1) and SM(36.2) shows a significant improvement in the models ability to classify between Human milk and Formula and Human mixed milk (from a 0.5 in permuted data to a 0.83 ROC AUC value) in the CBGS1 data. Moreover, as seen in Fig. 3b direct visualization of the selected lipids in a box plot, allows for a clear display of the differing prevalence of this feature in babies fed with these different milk nutrition and so explaining its selection and inclusion in the classification model. These plots are easy to interpret and hence reach out to a non-expert domain. Moreover, our analysis revealed a consistent biomarker robustness, between HM and FM diets, across three different cohorts, summarised in the Additional file 2. For example, SM(39:1) is identified as a robust biomarker for segregating infants on HM vs. FM diets (Additional file 2: Table 2).

#### Transcriptomics data

In the pancreatic cancer dataset GSE15471, top features selected included the following 20 genes: SULF1, COL10A1, MIR34AHG, INHBA, COL8A1, FN1, THBS2, NOX4, NTM, RASAL2, ADAMTS12, CAPG, CTHRC1, FAP, VCAN, SLPI, WISP1, LTBP1, GPRC5A, TIMP1. Our biological pathway analysis revealed a class of biomarkers enriched in several known cancer pathways. After stringent multiple testing correction, the following pathways were identified as being enriched:

- Senescence and Autophagy in Cancer
- Integrated Pancreatic Cancer Pathway
- TGF Beta Signalling Pathway (mouse)
- miRNA targets in ECM and membrane receptors
- TGF Beta Receptor Signalling (human).

The gene set was further enriched in the Human Proteome Map in adult Esophagus, Lung, and Pancreas tissues, indicating a potential cross-talk among tissue-specific cancer pathways. The full results of our pipeline, including feature ranking graphs and ROC AUC, sensitivity, specificity, and accuracy scores of each variable, as well as all the executed code are included in Additional file 1.

In addition to the validation of our method against pancreatic cancer, a validation was performed against an acute myeloid leukaemia (AML) cohort. This analysis revealed sixty genes contributing significantly to AML (see Additional file 3). This gene set interacts with several AML-associated transcription factors, including NKX2-3, HOXA7, and MYB. The analysis of genes active in cell lines available in the Cancer Cell Line Encyclopedia [23] revealed that our derived gene set is significantly enriched in multiple haematopoietic and lymphoid tissue lines. Additionally, investigation of the presence of our depicted genes in biological pathways, annotated in the KEGG database [24], confirmed their known AML-gene associations mediated by the ‘Hematopoietic stem cell lineage’ and ‘Transcriptional misregulation in cancer’ pathways. Further results from these analyses are presented in Additional file 3.

## Discussion

We developed a systematic way of analysing -omics datasets to identify potential biomarkers from large-scale -omics datasets. We used three different datasets (two transcriptomics and one lipidomics) to validate our approach by identifying potential markers or signatures and comparing with existing markers found in the literature.

### Lipidomics data

Acharjee et al. [16] investigated and identified relevant lipid biomarkers that were able to predict infant feeding patterns. A narrow down list of candidate biomarkers was produced based on a combination of supervised Random Forests and iterative backward elimination. In our analysis, we used two methods, LASSO and EN, that perform automatic feature selection. Our analysis revealed two types of relevant lipids; four Sphingomyelin and two Phosphocholine. Out of these six, three were previously reported by Acharjee et al. [16]. For our validation analysis, selected features were singled out in one step, whereas in the previous study a two-step procedure was employed. Moreover, to assess the stability of the relevant identified features, we employed multiple sampling and permutation testing to test against an empirical null distribution based on ROC AUC scores (Fig. 3a).

### Transcriptomics data

In order to assess the wider applicability of our approach for identifying target molecules in different types of -omics data, we also applied it in two transcriptomic datasets, one for Acute Myeloid Leukemia and one for Pancreatic Ductal AdenoCarcinoma (PDAC). Whereas the lipidomic analysis could be validated by expert curation, our transcriptomic analyses were validated via external pathway and ontology gene set enrichment tools.

AML driver gene analysis revealed a set of genes known to be enriched for targets of the MYB transcription factor. MYB is known to play a crucial role in hematopoietic stem cell cycles, including proliferation and survival, and recent research has shown that AML-specific microRNAs target c-MYB [25]. Additionally, a potentially drugable compound targeting MYB was recently discovered [26], highlighting the clinical role of MYB targets. By highlighting the genes which are both predictors of AML and enriched as a set for MYB targeting, we have identified a set of novel gene targets of the MYB transcription factor.

The genes identified by our pipeline are often discussed in pancreatic cancer literature [27]. Not only did we identify gene sets in relevant tissues which are, in combination, highly discriminative between pancreatic cancer and control, but the in-built multivariate analysis revealed interacting networks which model differences between cancer and control patient data better than single genes alone. Our analysis also highlighted the cross-talk between autophagy and certain cancer types. Given the prevalence of autophagy pathways perturbed in pancreatic cancers, this result confirms recent novel studies demonstrating autophagic control of pancreatic cancer metabolism [28, 29].

### Workflow features

In high-dimensional -omics data analysis, we are interested in finding a relevant smaller subset of variables that are associated with the response (a clinical phenotype). Procedures to identify such smaller subsets are called variable or feature selection procedures. By employing such procedures, it is possible to reduce the dimensionality of the data [30]. Moreover, feature selection can assist in removing noise variables (variables which have no predictive power for the response variable) in the dataset. More specifically, typical reasons to employ feature selection procedure include: large number parameters, features or variables (p) compared to the number of the samples or individuals (n) and correlated features.

One advantage of using feature selection algorithms is that the final model is built automatically, including only those biomarkers which are useful in predicting patient condition. Thus, we do not have to rely on the cut off for selection of genes and metabolites upfront. All the estimates are decided based on either biomarkers’ effects. However, one of the drawbacks of this method lies with the selection of the appropriate penalty parameters. Failure to decide on appropriate penalty factor will result in underfitting or over fitting of the results. To address this, we split the data into two subsets, training and testing. Within the training subset, we estimated the penalty factor by using ten-fold cross validation. The optimized model was then fit to the unseen testing subset.

While either LASSO or EN can be used for both classification and regression tasks, our method focused on validation tasks based on binary outcomes (classification). In a planned update of the software accompanying our method, we will enable users to switch between classification and regression tasks. Users will also be able to choose between different feature selection algorithms and machine learning models including Random Forests, Artificial Neural Networks, and Deep Leaning which can capture alternative patterns of interactions in the data that we might miss out with regularized linear models [31]. We also plan to implement our code in a portable Docker environment to eliminate the need for end users from dealing with version control and software dependencies. Lastly, it should be noted that our model currently accepts numerical variables whilst categorical variables should be dummy (one-hot) encoded.

A unique strength of our approach lies with the provision of automated pre-processing and feature selection. Based on our approach, we were able to reduce the number of potential causative genes in each experiment to under 100 (from an input of over 22,000 genes) whilst the high confidence selections were reduced to less than 15. This robust selection creates a useful feature for end users, eliminating the need to pre-filter data based on perceived biological knowledge thus eliminating bias.

### Future trends

#### Multi -omics data integration

To completely understand the underlying biological mechanisms driving diverse phenotypes, a multi-omics approach is often necessary. However, this is a challenging step due to the data size, measurements, and data analysis involved. Different approaches are currently suggested in the literature to link or integrate them. For example, Shen et al. used multi-omics datasets which include copy number, gene expression, and methylation data from TCGA in an unsupervised matrix factorization algorithms using the software i-Cluster [32]. Seoane et al. used a pathway-based data integration framework for prediction of breast cancer progression. They used multiple kernel learning supervised learning methods on multi-omics datasets that includes clinical data, gene expression and copy number data [33]. A similar method was further applied by Zhu et al. to integrate somatic mutation, DNA copy number, DNA methylation, mRNA and miRNA expression datasets from TCGA [34]. Acharjee et al. used Random Forests to integrate clinical, lipidomics, and metabolomics datasets. They first selected features from metabolomics and lipidomicsdataset and linked selected features by correlation analysis [35]. Pedersen et al. developed a computational framework to integrate multi-omics datasets that included human phenotype, serum metabolome and gut microbiome data. This framework allowed for a stepwise flexible choice of methods, adaptable to different -omics datasets with feature selection as one of the important first step. Additional examples of multiomics integration include linking genome, metabolome and gut microbiome [36], and the linking of somatic mutations, RNA expression, DNA methylation and ex vivo drug responses [37].

In addition to -omics datasets, there are other unstructured clinical phenotypic datasets such as medical images, electronic health records, and medical questionnaires. These pose new challenges for data integration and reproducibility that needs standardization and to put into clinical practices [38]. Proposed strategies for integrating these data into our current pipeline include deriving numerical features from these unstructured data, for example by creating vectors of word representations with word2vec models [39].

#### Single cell sequencing

It is worth mentioning that certain areas of precision medicine benefit greatly from incorporating single-cell sequencing data, especially cancer. While multiple -omics approaches can be used with single-cell sequencing [40, 41], RNA-Sequencing applied to single-cell data has been used extensively, and we will focus our discussion on this area [42–44]. Single-cell transcriptomics (scRNA-Seq) have potential for monitoring patient response to treatment and characterizing lineage-specific mutations which may respond to variable treatment protocols. Before incorporating scRNA-Seq data into the pipelines described above, experimentalists and analysists must be aware of several differences in protocol which affect normalization of scRNA-Seq data. Stegele et al. [45] produced a fundamental review of challenges which are currently being addressed by the community. In essence, data must be carefully curated, select data must be normalized after additional quality controls not applicable to bulk RNA-Seq. This may necessitate the inclusion of synthetic or alternate species controls (spike-ins) in the sequencing experiments not always used in bulk data analysis. After normalization, populations of cells may be identified by several unsupervised learning methods, from clustering to tSNE [46]. Our pipeline may add value to single-cell analyses by picking up at this stage and integrating count matrices of cell-types separated by clustering approaches. With the separation of cell populations, dominated by driver genes characterizing cell types or disease states as labels, our pipeline can be applied to select gene transcripts which act as biomarkers. With these biomarkers identified, subsequent patient monitoring may be applied to surveil tissues for tumour progression and guide the application of treatment or help reveal mechanisms in cells which survive treatment after resequencing [44]. Finally, it is worth mentioning too, that useful clinical translation will only follow from a better understanding of the underlying biological mechanisms for the biomarker´s discovered.

The application of single-cell omics to our pipeline may be useful in model organism and basic research to guide future translational projects or prioritize experiments for biomarker validation.

## Conclusion

We present a data-driven, generalizable, robust, low-bias machine learning workflow that generates easily interpretable outputs and focus on simple visualizations aiming at actionable biomarker discovery. We believe that our workflow will help researchers to identify significant explanatory features of experimental -omics data, reducing the search space for good candidates for experimental validation and follow up. Robustly optimizing feature selection to changes in data perturbation provides a high confidence in the selection of potential novel features, which forms a crucial advantage in translational medicine applications.

## Additional files

**Additional file 1.** R Markdown analysis results from the workflow developed on GSE15471.

**Additional file 2.** Lipids identified in three cohorts are listed with different category. Category A: HM vs. Mixed (FM and HM combined) feeding; Category B: FM vs. Mixed (FM and HM combined ) feeding; Category C: HM vs. FM.

**Additional file 3.** R Markdown analysis results from the workflow developed on GSE15061.

## Data Availability

The datasets analysed during the current study are available in the Gene Expression Omnibus. Transcriptomic datasets accessed include https://www.ncbi.nlm.nih.gov/geo/query/acc.cgi?acc=GSE15471 and https://www.ncbi.nlm.nih.gov/geo/query/acc.cgi?acc=GSE15061. Lipidomics data were analysed from the supplementary materials of following article: https://www.ncbi.nlm.nih.gov/pmc/articles/PMC5272886/.

## Abbreviations

AML: acute myeloid leukemia
CV: cross validation
EN: Elastic Net
FM: formula milk
HM: human milk
miRNAs: microRNAs
LASSO: Least Absolute Shrinkage and Selection Operator
PDAC: pancreatic ductal adenocarcinoma
ROC AUC: Receiver Operating Characteristic Area Under the Curve
RSS: residual sum of squares
SM: sphingomyelin
SNP: single nucleotide polymorphism
PC: phosphatidylcholine
TM: translational medicine
