# Supplementary material for: -Omics biomarker identification pipeline for translational medicine
Source: J Transl Med. 2019 May 14;17:155. doi: 10.1186/s12967-019-1912-5 (PMC6518609; doi:10.1186/s12967-019-1912-5)
Supplement: Supplementary file 1 — Additional file 1. R Markdown analysis results from the workflow developed on GSE15471. [file 12967_2019_1912_MOESM1_ESM.pdf]

Pancreatic Cancer Analysis

Bravo, Williams, and Acharjee

19 Sept 2018

```r
#Libraries



##### Begin Block 1

setwd("~/AnimeshReview/LauraPaper/Journal of Translational Medicine /LauraFinal/")
library(readxl)
library(data.table)
library(tidyverse)
library(dplyr)
library(devtools)
library(ggcorrplot)
library(car)
library(ggpubr)
library(glmnet)
library(summarytools)
library(knitr)
library(htmltools)
library(corrplot)
library(caret)
library(factoextra)
library(Metrics)
library(readr)
library(gplots)
library(dplyr)
library(stringr)
library(readxl)
library(plotly)
library(e1071)
library(ggplot2)
library(reshape2)
library(multtest)
library(ROCR)
library(gridExtra)
library(MLmetrics)


#### Begin Load Laura's Functions
doubleAUCfun <- function(xtrain, ytrain,xtest,ytest,s,k) {

    yy3<-data.frame(Outcome=ytrain, Predictor=xtrain[,s],Predictor2=xtrain[,k])
    model3<-glm(Outcome~Predictor+Predictor2,data=yy3, family=binomial(link='logit'))
    new3<-data.frame(Predictor=xtest[,s],Predictor2=xtest[,k])
    p3<-predict(model3,new3,type="response")
    new5<-data.frame(Outcome=ytest)
    pr3 <- prediction(p3, new5)
```

```
    prf3 <- performance(pr3, measure = "tpr", x.measure = "fpr")
    auc3 <- performance(pr3, measure = "auc")
    auc3 <- auc3@y.values[[1]]
    result2<-auc3
}

doubleAUCfunNB <- function(xtrain, ytrain,xtest,ytest,s,k) {

    yy3<-data.frame(Outcome=ytrain, Predictor=xtrain[,s],Predictor2=xtrain[,k])
    model4<-naiveBayes(Outcome~Predictor+Predictor2,data=yy3)
    new4<-data.frame(Predictor=xtest[,s],Predictor2=xtest[,k])
    p4<-predict(model4,new4,type="raw")
    #without raw write confusion matrix directly
    prob2<-NULL
    for (i in 1:dim(p4)[1]){
        prob2[i]<-p4[i,2]/p4[i,1]
    }
    new5<-data.frame(Outcome=ytest)
    pr3 <- prediction(prob2, new5)
    prf3 <- performance(pr3, measure = "tpr", x.measure = "fpr")
    auc3 <- performance(pr3, measure = "auc")
    auc3 <- auc3@y.values[[1]]
    result2<-auc3
}

doubleAUCfunRFCross <- function(xtrain, ytrain,xtest,ytest,s,k) {

    yy3<-data.frame(Outcome=ytrain, Predictor=xtrain[,s],Predictor2=xtrain[,k])
    control <- trainControl(method="repeatedcv", number=10, repeats=3) #Is this the only thing needed f
    seed <- 7
    metric <- "Accuracy"
    set.seed(seed)
    #mtry <- 2
    tunegrid <- expand.grid(.mtry=c(1:6))
    set.seed(seed)
    rf_default <- train(Outcome~Predictor+Predictor2,data=yy3, method="rf", metric=metric, tuneGrid=tun
    #print(rf_default)
    new4<-data.frame(Predictor=xtest[,s],Predictor2=xtest[,k])
    pred=predict(rf_default, new4,type="prob")
    pred2=predict(rf_default, new4)
    prob2<-NULL
    for (i in 1:dim(pred)[1]){
        prob2[i]<-pred[i,2]/pred[i,1]
    }
    new5<-data.frame(Outcome=ytest)
    pr3 <- prediction(prob2, new5)
    prf3 <- performance(pr3, measure = "tpr", x.measure = "fpr")
    auc3 <- performance(pr3, measure = "auc")
    auc3 <- auc3@y.values[[1]]
    result2<-auc3

}
```

```
doubleAUCfunSVM <- function(xtrain, ytrain,xtest,ytest,s,k) {

    yy3<-data.frame(Outcome=ytrain, Predictor=xtrain[,s],Predictor2=xtrain[,k])
    seed <- 7
    set.seed(seed)
    model3<-svm(Outcome~Predictor+Predictor2,data=yy3)
    new3<-data.frame(Predictor=xtest[,s],Predictor2=xtest[,k])
    p3<-predict(model3,new3,decision.values = TRUE)
    p4<-attr(p3,"decision.values")
    new5<-data.frame(Outcome=ytest)
    pr3<- prediction(p4, new5)
    #table(p3, new5)
    #confusionMatrix(p3, new5)
    prf3 <- performance(pr3, measure = "tpr", x.measure = "fpr")
    auc3 <- performance(pr3, measure = "auc")
    auc3 <- auc3@y.values[[1]]
    result2<-auc3
}

doubleAUCfunSVMCross <- function(xtrain, ytrain,xtest,ytest,s,k) {

    yy3<-data.frame(Outcome=ytrain, Predictor=xtrain[,s],Predictor2=xtrain[,k])
    seed <- 7
    set.seed(seed)
    model4<-svm(Outcome~Predictor+Predictor2,data=yy3,method="C-classification",
                kernel="radial", gamma = 0.01, cost = 100,cross=10, probability=TRUE)
    new4<-data.frame(Predictor=xtest[,s],Predictor2=xtest[,k])
    p4<-predict(model4,new4,decision.values = TRUE)
    p5<-attr(p4,"decision.values")
    new5<-data.frame(Outcome=ytest)
    pr3 <- prediction(p5, new5)
    prf3 <- performance(pr3, measure = "tpr", x.measure = "fpr")
    auc3 <- performance(pr3, measure = "auc")
    auc3 <- auc3@y.values[[1]]
    result2<-auc3
}

doublePlusfun <- function(xtrain, ytrain,xtest,ytest,s,k) {

    yy3<-data.frame(Outcome=ytrain, Predictor=xtrain[,s],Predictor2=xtrain[,k])
    model3<-glm(Outcome~Predictor+Predictor2,data=yy3, family=binomial(link='logit'))
    new3<-data.frame(Predictor=xtest[,s],Predictor2=xtest[,k])
    p3<-predict(model3,new3,type="response")
    new5<-data.frame(Outcome=ytest)
    pr <- prediction(p3, new5)
    Acc <- performance(pr, measure="acc")
    AccV<-Acc@y.values[[1]][max(which(Acc@x.values[[1]] >= 0.5))]
    Sens <- performance(pr, measure= "sens")
    SensV<-Sens@y.values[[1]][max(which(Sens@x.values[[1]] >= 0.5))]
    Spec <- performance(pr, measure= "spec")
    SpecV<-Spec@y.values[[1]][max(which(Spec@x.values[[1]] >= 0.5))]
    Prec <- performance(pr, measure= "prec")
    PrecV<-Prec@y.values[[1]][max(which(Prec@x.values[[1]] >= 0.5))]
```

```
    AllV<-data.frame(Vector=c(AccV, SensV, SpecV, PrecV))
    return(AllV)
}

doubleROCfun <- function(xtrain, ytrain,xtest,ytest,s,k) {

    yy3<-data.frame(Outcome=ytrain, Predictor=xtrain[,s],Predictor2=xtrain[,k])
    model3<-glm(Outcome~Predictor+Predictor2,data=yy3, family=binomial(link='logit'))
    new3<-data.frame(Predictor=xtest[,s],Predictor2=xtest[,k])
    p3<-predict(model3,new3,type="response")
    new5<-data.frame(Outcome=ytest)
    pr3 <- prediction(p3, new5)
    prf3 <- performance(pr3, measure = "tpr", x.measure = "fpr")
    result<-prf3
    auc3 <- performance(pr3, measure = "auc")
    auc3 <- auc3@y.values[[1]]
    return(result)

}

MeansNames <- function(doubleAUCR,trial){

    doubleAUCR<-as.data.frame(doubleAUCR)
    doubleAUCR<-mutate(doubleAUCR, Means=rowMeans(doubleAUCR))
    row.names(doubleAUCR)<-trial
    return(doubleAUCR)
}

multipleAUCfun <- function(xtrain,ytrain,xtest,ytest) {

    yy3<-data.frame(Outcome=ytrain,xtrain)
    model3<-glm(Outcome~.,data=yy3, family=binomial(link='logit'))
    new3<-data.frame(xtest)
    p3<-predict(model3,new3,type="response")
    new5<-data.frame(Outcome=ytest)
    pr3 <- prediction(p3, new5)
    prf3 <- performance(pr3, measure = "tpr", x.measure = "fpr")
    auc3 <- performance(pr3, measure = "auc")
    auc3 <- auc3@y.values[[1]]
    result2<-auc3
    return(result2)
}

multipleAUCfunNB <- function(xtrain, ytrain,xtest,ytest) {

    yy3<-data.frame(Outcome=ytrain,xtrain)
    model4<-naiveBayes(Outcome~.,data=yy3)
    new3<-data.frame(xtest)
    p4<-predict(model4,new3,type="raw")
    #without raw write confusion matrix directly
    prob2<-NULL
    for (i in 1:dim(p4)[1]){
        prob2[i]<-p4[i,2]/p4[i,1]
```

```r
    }
    new5<-data.frame(Outcome=ytest)
    pr3 <- prediction(prob2, new5)
    prf3 <- performance(pr3, measure = "tpr", x.measure = "fpr")
    auc3 <- performance(pr3, measure = "auc")
    auc3 <- auc3@y.values[[1]]
    result2<-auc3
    return(result2)
}

multipleROCfun <- function(xtrain,ytrain,xtest,ytest) {

    yy3<-data.frame(Outcome=ytrain,xtrain)
    model3<-glm(Outcome~.,data=yy3, family=binomial(link='logit'))
    new3<-data.frame(xtest)
    p3<-predict(model3,new3,type="response")
    new5<-data.frame(Outcome=ytest)
    pr3 <- prediction(p3, new5)
    prf3 <- performance(pr3, measure = "tpr", x.measure = "fpr")
    auc3 <- performance(pr3, measure = "auc")
    auc3 <- auc3@y.values[[1]]
    result2<-auc3
    return(prf3)

}

multipleROCfunNB <- function(xtrain, ytrain,xtest,ytest) {

    yy3<-data.frame(Outcome=ytrain,xtrain)
    model4<-naiveBayes(Outcome~.,data=yy3)
    new3<-data.frame(xtest)
    p4<-predict(model4,new3,type="raw")
    #without raw write confusion matrix directly
    prob2<-NULL
    for (i in 1:dim(p4)[1]){
        prob2[i]<-p4[i,2]/p4[i,1]
    }
    new5<-data.frame(Outcome=ytest)
    pr3 <- prediction(prob2, new5)
    prf3 <- performance(pr3, measure = "tpr", x.measure = "fpr")
    auc3 <- performance(pr3, measure = "auc")
    auc3 <- auc3@y.values[[1]]
    result2<-auc3
    return(prf3)
}

singleAUCfun <- function(xtrain, ytrain,xtest,ytest,s) {

    yy2<-data.frame(Outcome=ytrain, Predictor=xtrain[,s])
    model2<-glm(Outcome~Predictor,data=yy2,family=binomial(link='logit'))
    new2<-data.frame(Predictor=xtest[,s])
    p<-predict(model2,new2,type = "response")
    new4<-data.frame(Outcome=ytest)
```

```
    pr <- prediction(p, new4)
    prf <- performance(pr, measure = "tpr", x.measure = "fpr")
    auc <- performance(pr, measure = "auc")
    auc <- auc@y.values[[1]]
    return(auc)
}

singlePlusfun <- function(xtrain, ytrain,xtest,ytest,s) {


    yy2<-data.frame(Outcome=ytrain, Predictor=xtrain[,s])
    model2<-glm(Outcome~Predictor,data=yy2,family=binomial(link='logit'))
    new2<-data.frame(Predictor=xtest[,s])
    p<-predict(model2,new2,type = "response")
    new4<-data.frame(Outcome=ytest)
    pr <- prediction(p, new4)
    Acc <- performance(pr, measure="acc")
    AccV<-Acc@y.values[[1]][max(which(Acc@x.values[[1]] >= 0.5))]
    Sens <- performance(pr, measure= "sens")
    SensV<-Sens@y.values[[1]][max(which(Sens@x.values[[1]] >= 0.5))]
    Spec <- performance(pr, measure= "spec")
    SpecV<-Spec@y.values[[1]][max(which(Spec@x.values[[1]] >= 0.5))]
    Prec <- performance(pr, measure= "prec")
    PrecV<-Prec@y.values[[1]][max(which(Prec@x.values[[1]] >= 0.5))]
    AllV<-data.frame(Vector=c(AccV, SensV, SpecV, PrecV))
    return(AllV)
}

singleROCfun <- function(xtrain, ytrain,xtest,ytest,s) {

    yy2<-data.frame(Outcome=ytrain, Predictor=xtrain[,s])
    model2<-glm(Outcome~Predictor,data=yy2,family=binomial(link='logit'))
    new2<-data.frame(Predictor=xtest[,s])
    p<-predict(model2,new2,type = "response")
    new4<-data.frame(Outcome=ytest)
    pr <- prediction(p, new4)
    prf <- performance(pr, measure = "tpr", x.measure = "fpr")
    resultU2<-prf
    tomeany<-as.data.frame(prf@y.values)
    tomeanx<-as.data.frame(prf@x.values)
    auc <- performance(pr, measure = "auc")
    auc <- auc@y.values[[1]]
    return(resultU2)

}





set.seed(132)
```

```
### Load Pancreatic Cancer Dta
setwd("/Users/j.williams/AnimeshReview/LauraPaper/Journal of Translational Medicine /LauraFinal/Sept18_
All2 <- readRDS("All2.Pancreatic.rds"); savee <- readRDS("savee.Pancreatic.rds")





#TRAIN

### set n to 10 for now to make quicker
n<-100 #Number of LASSO and EN loops. Per loop a model with features selected. The 80% most popular fea

#N and nn control the number of models created for combinatorial analysis to help in further feature se
N <-10
nn <-25

ErrorsFinEN<-vector(mode="double", length=n)
BetasFinEN<-vector(mode="character", length=n)
LambdaFinEN<-vector(mode="double", length=n)
BNumFinEN<-vector(mode="double", length=n)
see2EN<-data.frame(All="All")
LauCoef1<-data.frame(Coeff="See",stringsAsFactors=FALSE)
BetasTodo<-data.frame(Features="Name",Coefficients=1)

ListError<-vector(mode="double", length=n)
BetasFin<-vector(mode="character", length=n)
LambdaFin<-vector(mode="double", length=n)
BNumFin<-vector(mode="double", length=n)
see2<-data.frame(All="All")
LauCoef1L<-data.frame(Coeff="See",stringsAsFactors=FALSE)
BetasTodoL<-data.frame(Features="Name",Coefficients=1)

##### End Block 1
```

```
##### Begin Block 2


for (i in 1:n){

  smp_size = floor(0.75 * nrow(All2))

  train_ind <- caret::createDataPartition(All2$Label, p = 0.75, list = FALSE, times = 1)

  #Training set
  train = All2[train_ind, ]

  #Test set
  test = All2[-train_ind, ]

  #Creates matrices for independent and dependent variables.
  xtrain <- train[ , !(names(train) %in% "Label")] %>% as.matrix()
  ytrain = train$Label
```

```
xtest <- test[ , !(names(test) %in% "Label")] %>% as.matrix()
ytest = test$Label


#Choose lambda value that minimize missclassification error.
#0.5 as elastic nets, all variables with EN are based on ElasticNets analysis. 100 lambdas sampled wi
CVEN=cv.glmnet(xtrain,ytrain,family="binomial",type.measure="class",alpha=0.5,nlambda=100)
attach(CVEN)
Lambda.BestEN<-CVEN$lambda.min #can be either minimum or 1 standard deviation
print(Lambda.BestEN)

CVFinEN=glmnet(xtrain,ytrain,family="binomial",alpha=0.5,lambda=Lambda.BestEN)
CoefEN<-coef(CVFinEN) #Beta coefficients obtained from here
InterceptEN<-CoefEN@x[1]
BetasEN<-CVFinEN$beta
Betas2EN<-data.frame(Features=BetasEN@Dimnames[[1]][BetasEN@i+1], Coefficients=BetasEN@x) #Beta coeff
CVPred1EN = predict(CVFinEN, family="binomial", s=Lambda.BestEN, newx = xtest,type="class") #predict

#Calculate error for categorical values
ytest2<-as.factor(ytest)
ResultsEN<-table(CVPred1EN,ytest)
confusionMatrix(as.factor(CVPred1EN),ytest)
AccuracyEN<-(ResultsEN[1]+ResultsEN[4])/sum(ResultsEN[1:4])
ErrorEN<-1-AccuracyEN

LauCoef<-Betas2EN$Coefficients
LauCoefEN<-data.frame(Coeff=LauCoef,stringsAsFactors=FALSE)
LauCoef1<-rbind(LauCoef1,LauCoefEN)
BetasTodo<-rbind(BetasTodo,Betas2EN) #store coefficients and store betas

seeEN<-Betas2EN$Features
seeEN1<-data.frame(All=seeEN)
see2EN<-rbind(see2EN,seeEN1)   #all beta names stored


mEN<-count(see2EN, All) #frequency of the betas stored counted
see3EN<-toString(seeEN)
ErrorsFinEN[i]<-ErrorEN #error of the model stored
BetasFinEN[i]<-see3EN #name of features the model used
BNumFinEN[i]<-length(seeEN) #number of features the model used
LambdaFinEN[i]<-Lambda.BestEN #lambda chosen for model
detach(CVEN)

#Change between Lasso and EN, alpha=1 (*)
CV=cv.glmnet(xtrain,ytrain,family="binomial",type.measure="class",alpha=1,nlambda=100)

attach(CV)

Lambda.Best<-CV$lambda.min
CVFin=glmnet(xtrain,ytrain,family="binomial",alpha=1,lambda=Lambda.Best)
Coef<-coef(CVFin)
Intercept<-Coef@x[1]
Betas<-CVFin$beta
```

```
  Betas2<-data.frame(Features=Betas@Dimnames[[1]][Betas@i+1], Coefficients=Betas@x)
  CVPred1 = predict(CVFin, family="binomial", s=Lambda.Best, newx = xtest,type="class")


  #Calculate error for categorical values
  ytest2<-as.factor(ytest)
  confusionMatrix(as.factor(CVPred1),ytest)
  Results<-table(CVPred1,ytest)
  Accuracy<-(Results[1]+Results[4])/sum(Results[1:4])
  Error<-1-Accuracy

  #visual display of for

  BetasTodoL<-rbind(BetasTodoL,Betas2)
  see<-Betas2$Features
  see1<-data.frame(All=see)
  see2<-rbind(see2,see1)
  m<-count(see2, All)

  see3<-toString(see)
  ListError[i]<-Error
  BetasFin[i]<-see3
  BNumFin[i]<-length(see)
  LambdaFin[i]<-Lambda.Best
  detach(CV)

}
```

```
## [1] 0.6689277
## [1] 0.6303686
## [1] 0.7294298
## [1] 0.2883708
## [1] 0.4733006
## [1] 0.5943159
## [1] 0.235993
## [1] 0.6584265
## [1] 0.1317309
## [1] 0.395802
## [1] 0.6748829
## [1] 0.6907273
## [1] 0.4808845
## [1] 0.2339522
## [1] 0.6926208
## [1] 0.4017377
## [1] 0.6815938
## [1] 0.3682275
## [1] 0.6878564
## [1] 0.5334466
## [1] 0.25522
## [1] 0.7062928
## [1] 0.5021274
## [1] 0.2454332
## [1] 0.3415769
## [1] 0.1445318
```

```
## [1] 0.2492792
## [1] 0.7286193
## [1] 0.4040431
## [1] 0.5608647
## [1] 0.4575239
## [1] 0.639378
## [1] 0.1181126
## [1] 0.194737
## [1] 0.1059382
## [1] 0.4479945
## [1] 0.3634272
## [1] 0.4787447
## [1] 0.5555517
## [1] 0.5364108
## [1] 0.1343051
## [1] 0.4800724
## [1] 0.3865033
## [1] 0.6247686
## [1] 0.2318932
## [1] 0.1884252
## [1] 0.6696531
## [1] 0.5132077
## [1] 0.502099
## [1] 0.629618
## [1] 0.06499661
## [1] 0.6696552
## [1] 0.3857286
## [1] 0.2742923
## [1] 0.2977953
## [1] 0.607225
## [1] 0.2129634
## [1] 0.4963329
## [1] 0.4782131
## [1] 0.2843202
## [1] 0.3724457
## [1] 0.4973665
## [1] 0.6293572
## [1] 0.251071
## [1] 0.7596586
## [1] 0.4509715
## [1] 0.4194573
## [1] 0.2410539
## [1] 0.6456791
## [1] 0.7355681
## [1] 0.631905
## [1] 0.69536
## [1] 0.5778142
## [1] 0.4760281
## [1] 0.642023
## [1] 0.36937
## [1] 0.4357793
## [1] 0.2766556
## [1] 0.5132012
## [1] 0.1636617
```

```
## [1] 0.7393992
## [1] 0.4958403
## [1] 0.172681
## [1] 0.6463179
## [1] 0.5903157
## [1] 0.393137
## [1] 0.7239418
## [1] 0.3621122
## [1] 0.6498011
## [1] 0.2339883
## [1] 0.5483706
## [1] 0.2081683
## [1] 0.5177668
## [1] 0.6192567
## [1] 0.609796
## [1] 0.2968183
## [1] 0.6362316
## [1] 0.4532429
## [1] 0.6682868
## [1] 0.4565895
```

```
#Visualizing data from LASSO and EN ####


#obtain in a data frame all error, betas names, number and lamda for the N models for each lasso and EN
All_info<-data.frame(Error=ListError, BetasNames=BetasFin, BetasNum=BNumFin, Lambda=LambdaFin)
All_infoEN<-data.frame(Error=ErrorsFinEN, BetasNames=BetasFinEN, BetasNum=BNumFinEN, Lambda=LambdaFinEN)

m<-m[-1,]
mEN<-mEN[-1,]

Final_LASSO<-m[order(-m$n),] #order highest frequencies above and filter with those that appear more th
Final_LASSO1<-filter(Final_LASSO,n>40) #threshold selected - 80%



Final_EN<-mEN[order(-mEN$n),]
Final_EN1<-filter(Final_EN,n>40)
Final_Plot_Names<-filter(Final_EN,n>40)

outputVenn2<-venn(list(EN= Final_EN$All, LASSO = Final_LASSO$All))
```

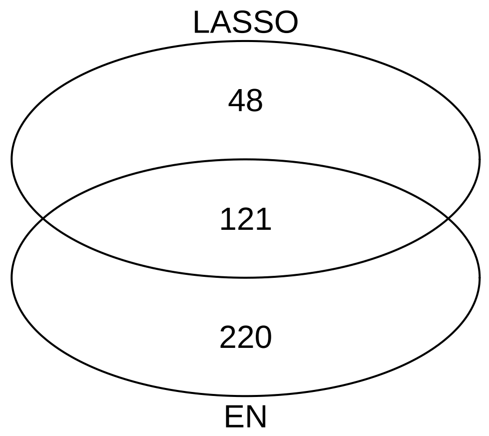


```
outputVenn<-venn(list(EN= Final_EN1$All, LASSO = Final_LASSO1$All))
```

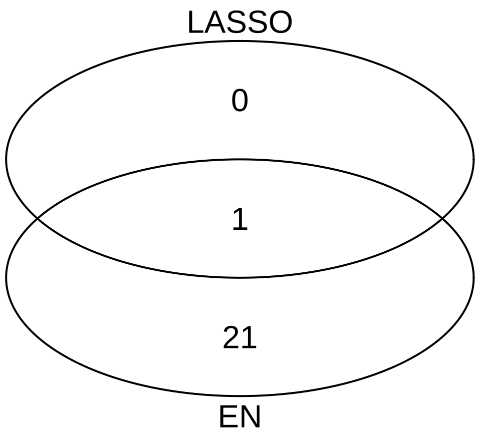


```
Freqs<-m[order(-m$n),]
num<-length(Freqs$All)

Freqs$All <- factor(Freqs$All, levels = Freqs$All[order(-Freqs$n)]) #plot in a bar graph the frequencie
ggplot(Freqs, aes(All, n))+geom_bar(stat="identity")+theme(axis.text.x = element_text(size=8, angle=90))
```

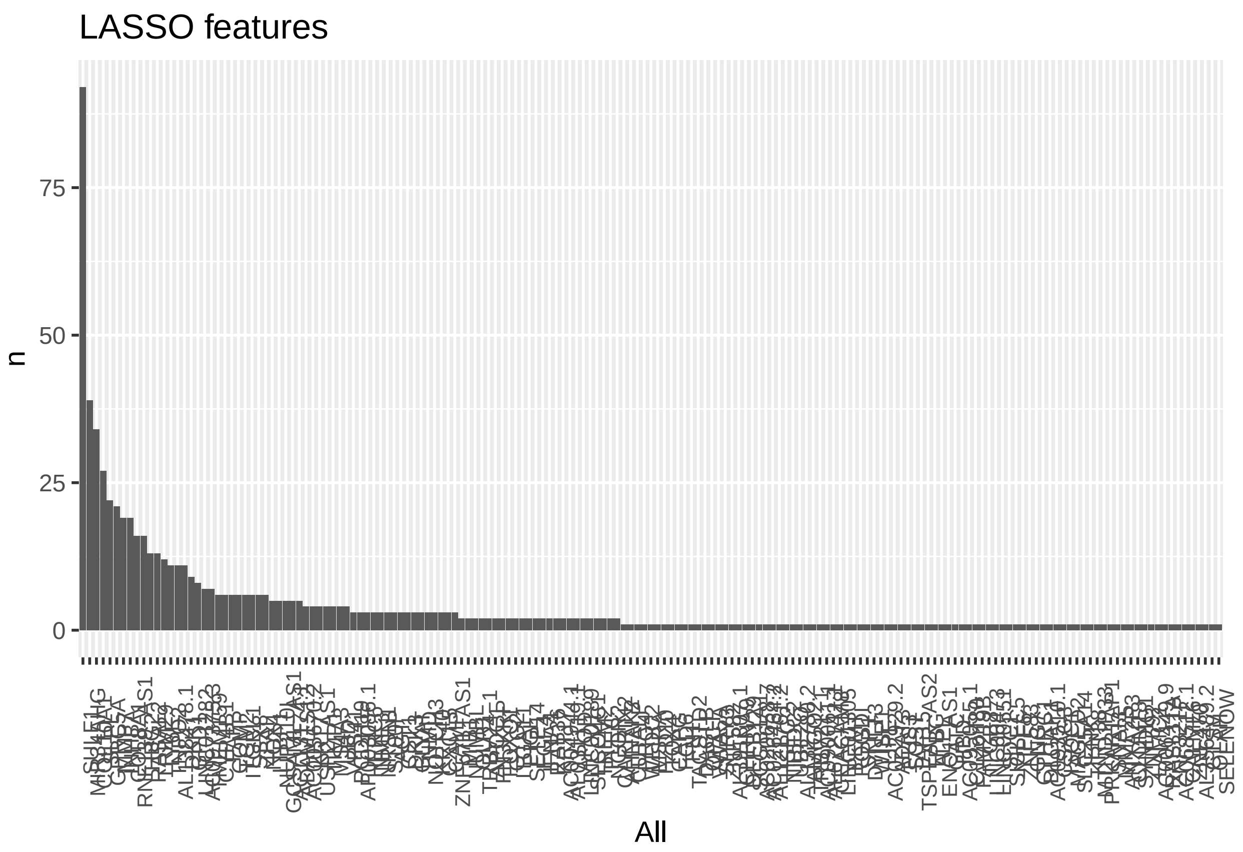


```
FreqsEN<-mEN[order(-mEN$n),]
numEN<-length(FreqsEN$All)

FreqsEN$All <- factor(FreqsEN$All, levels = FreqsEN$All[order(-FreqsEN$n)])
ggplot(FreqsEN, aes(All, n))+geom_bar(stat="identity")+theme(axis.text.x = element_text(size=8, angle=9
```

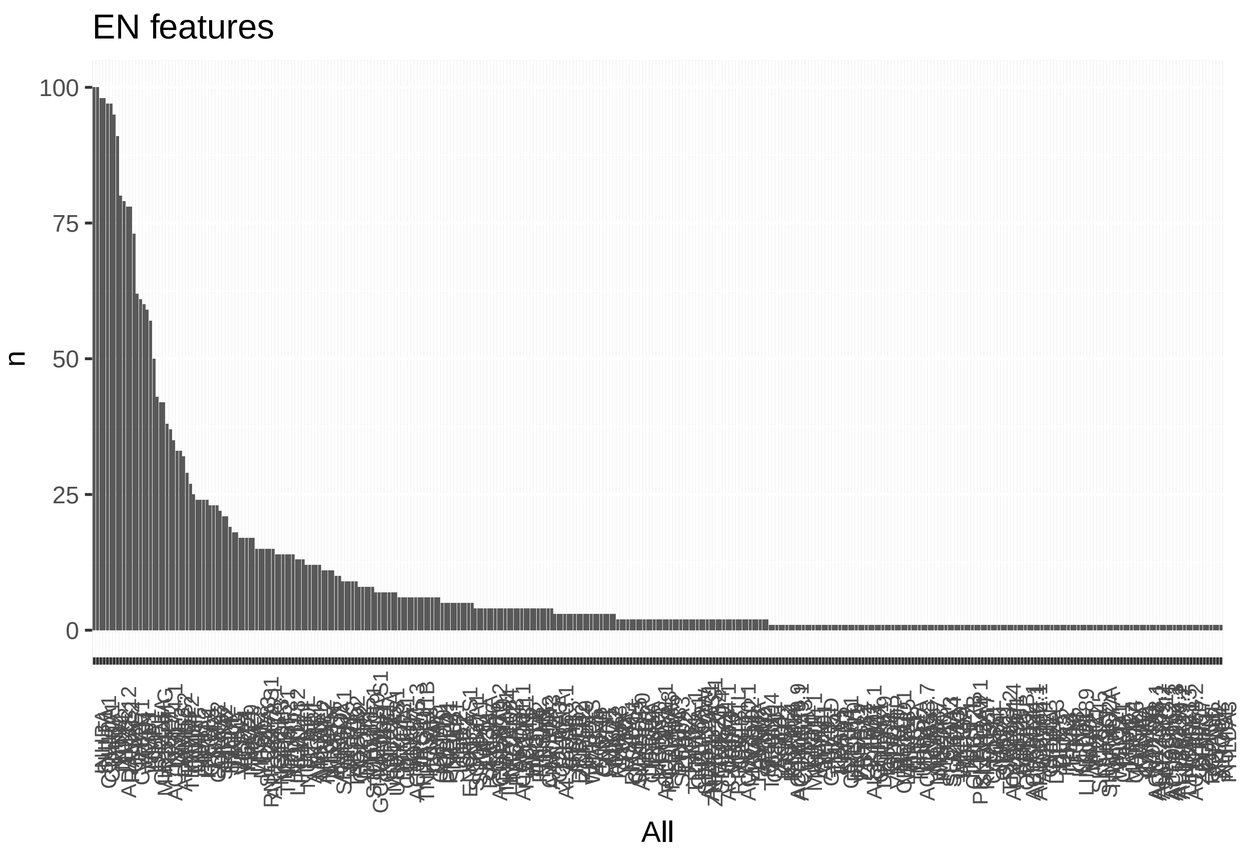


```r
#plot of how many times each feature appears. Most important will appear in all models = N.


#Boxplot with Betas and its coefficients

Boxplot1<-BetasTodo[BetasTodo$Features %in% Final_EN1$All,] #see which features appear in the filtered
ggplot(Boxplot1,aes(Boxplot1$Features,Boxplot1$Coefficients))+geom_boxplot()+geom_jitter()
```

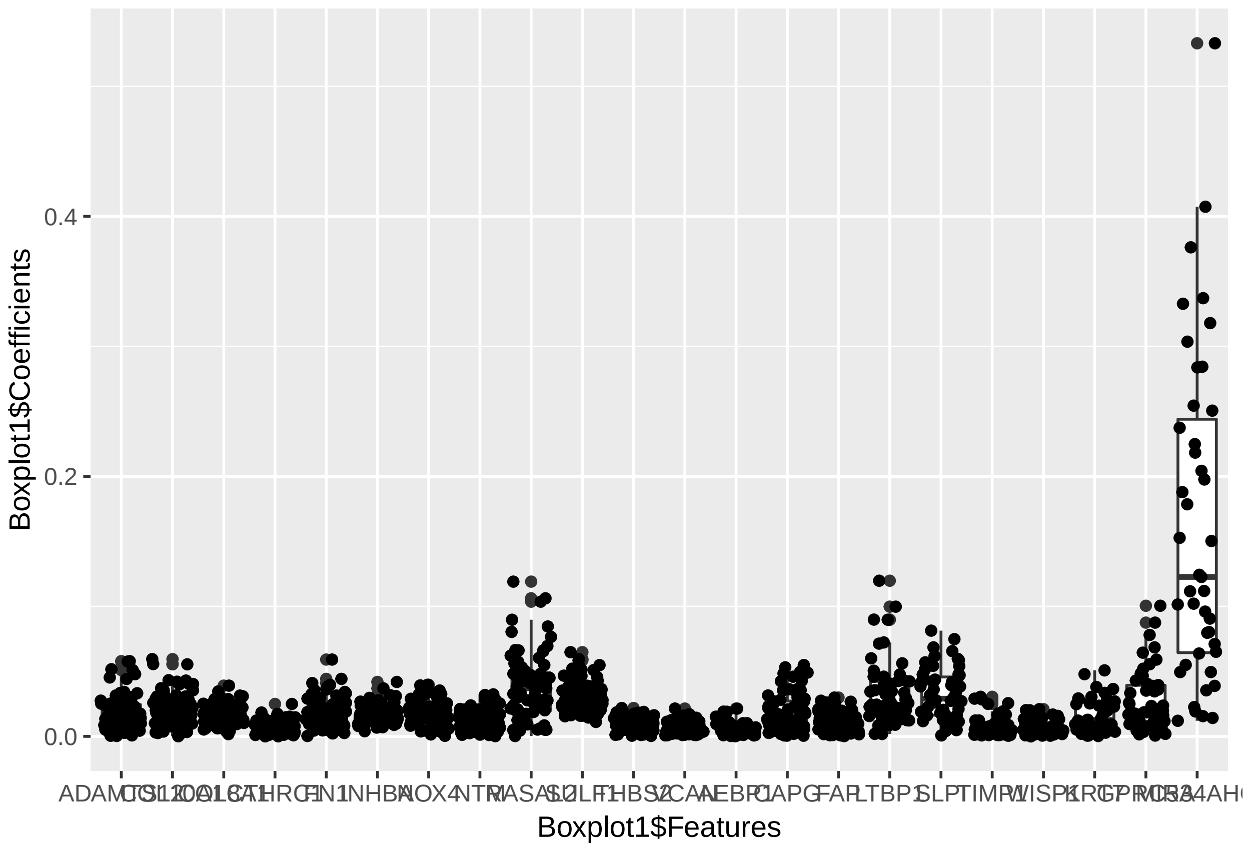


```
Boxplot1["Method"]<-as.factor("EN")

Boxplot2<-BetasTodoL[BetasTodoL$Features %in% Final_LASSO1$All,]
ggplot(Boxplot2,aes(Boxplot2$Features,Boxplot2$Coefficients))+geom_boxplot()+geom_jitter()
```

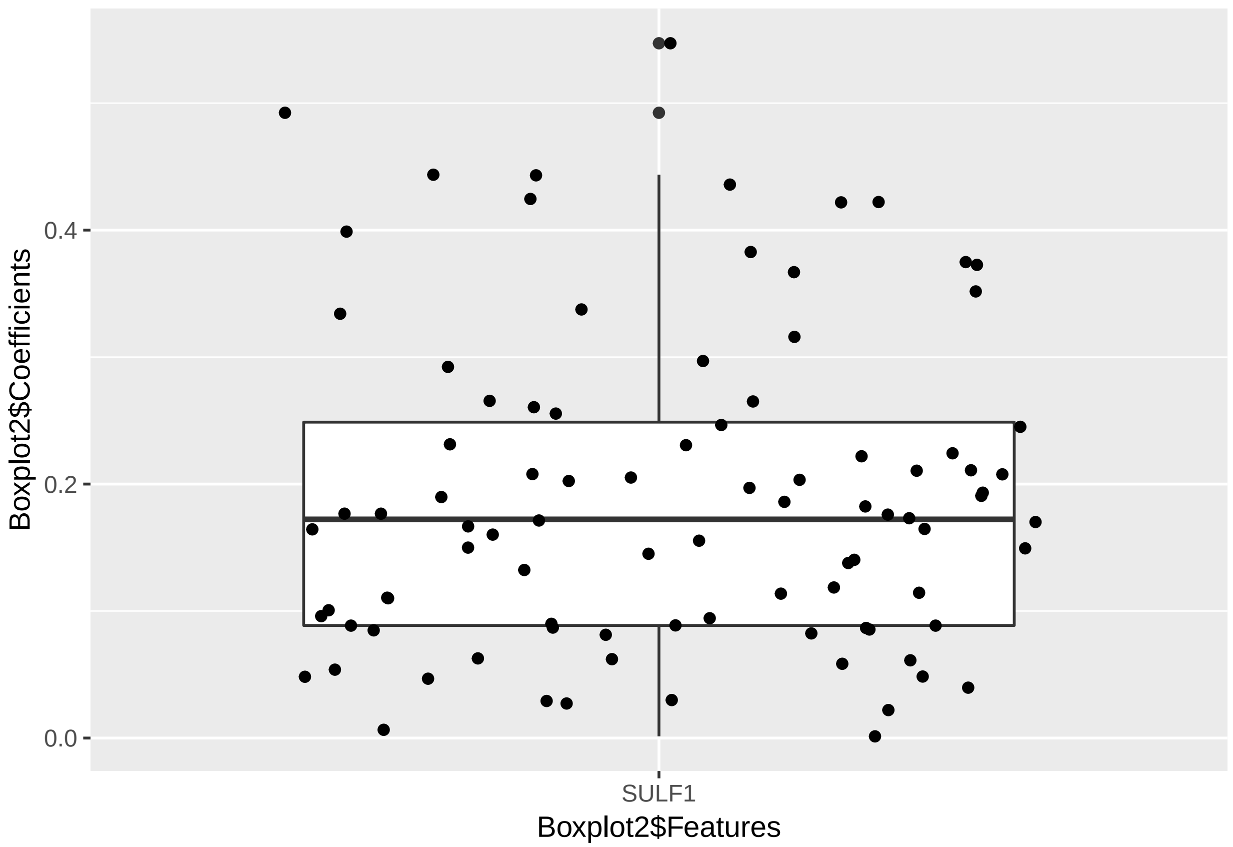


```
Boxplot2["Method"]<-as.factor("LASSO")

Fin_Boxplot<-rbind(Boxplot1,Boxplot2) #Unite both boxplots LASSO and EN
ggplot(Fin_Boxplot,aes(Fin_Boxplot$Features,Fin_Boxplot$Coefficients))+geom_boxplot(aes(color=Method))+g
```

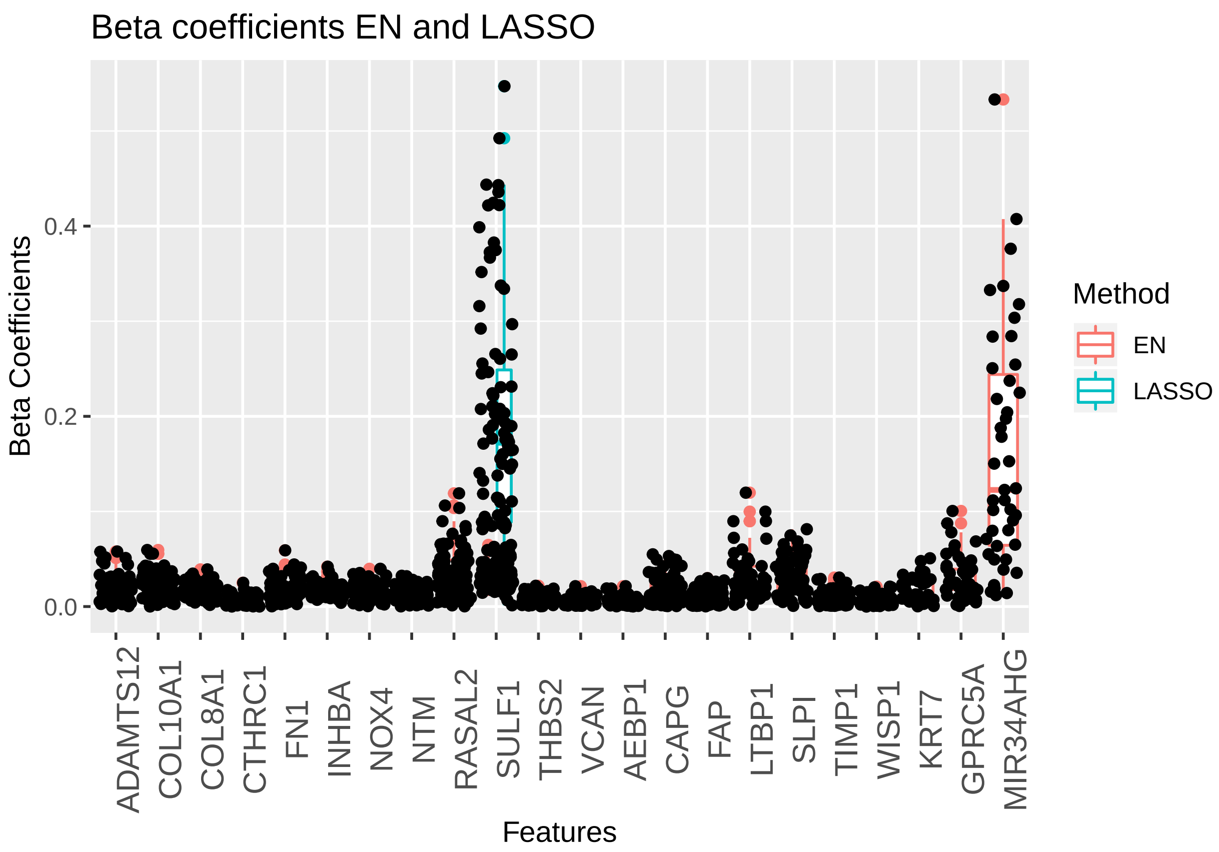


```
All_Feat<-rbind(Final_LASSO1,Final_EN1)
All_Feat2<-unique(All_Feat$All) #select the filtered betas in both.
#All_Feat2<-data.frame(All_Feat2)
Betas_select<-All2[,colnames(All2[,intersect(gsub("`", "", All_Feat2), colnames(All2))])]
Betas_select["Label"]<-All2$Label
Betas_select<-data.frame(Betas_select)

#Beta_select are the final features selected.

##### End Block 2
```

```
##### Begin Block 3

###Combinatorial analysis ####
NumVar<-length(Betas_select)
maxCombF<-data.frame(Name=toString("Trial"),AUC=0)
auc3max<-0
cont<-0
cont2<-0


for (p in 1:N){ #N different measurements of AUC values, mean done at the end.
  smp_size<-floor(0.65 * nrow(Betas_select))
  train_ind <- caret::createDataPartition(All2$Label, p = 0.65, list = FALSE, times = 1)

  # Training set
  train<-Betas_select[train_ind, ]
```

```
# Test set
test<-Betas_select[-train_ind, ]

xtrain <- train[ , !(names(train) %in% "Label")] %>% as.matrix()

xtest <- test[ , !(names(test) %in% "Label")] %>% as.matrix()

ytrain<-train$Label
ytest<-test$Label

xtest<-data.frame(xtest)
xtrain<-data.frame(xtrain)

y<-Betas_select[,NumVar]
X<-Betas_select[,1:(NumVar-1)]


levels(ytrain)[1]<-"0"
levels(ytrain)[2]<-"1"
levels(ytest)[1]<-"0"
levels(ytest)[2]<-"1"


for (k in 1:nn){
  columns<-c(1:dim(xtrain)[2])
  columns<-sample(columns)
  d<-xtrain[,columns]
  for (i in 1:dim(xtrain)[2]){
    for (j in 1:dim(xtrain)[2]){
      yy3<-data.frame(Outcome=ytrain,d[i:j])
      model3<-glm(Outcome~.,data=yy3, family=binomial(link='logit'))
      dd<-xtest[,columns]
      new3<-data.frame(dd[i:j])
      p3<-predict(model3,new3,type="response")
      new5<-data.frame(Outcome=ytest)
      pr3 <- prediction(p3, new5)
      prf3 <- performance(pr3, measure = "tpr", x.measure = "fpr")
      auc3 <- performance(pr3, measure = "auc")
      auc3 <- auc3@y.values[[1]]
      result2<-auc3
      if (auc3>auc3max){
        if (i>j){
          maxComb<-data.frame(Name=toString(names(d)[j:i]),AUC=auc3)
          auc3max<-auc3
          cont<-cont+1
        }
        else{

          maxComb<-data.frame(Name=toString(names(d)[i:j]),AUC=auc3)
          auc3max<-auc3
          cont<-cont+1
        }
      }
```

```
        else{
          cont2<-cont2+1
        }

      }
    }
  }
  maxCombF<-rbind(maxCombF,maxComb)
  auc3max<-0
}

maxCombF2<-maxCombF[order(maxCombF$AUC),]
names<-maxCombF2$Name[dim(maxCombF2)[1]]
names1<-as.character(names)
names1<-strsplit(names1,", ")
names<-as.data.frame(names1)

Betas_select2<-All2[,colnames(All2[,intersect(gsub("`", "", names[,1]), colnames(All2))])]
Betas_select2["Label"]<-savee
Betas_select2<-as.data.frame(Betas_select2)

##### End Block 3
```

```
##### Begin Block 4


#Random mix between variables and AUC value obtained through GLM model, rough approximation of future p
#Betas_select2, final features selected.


NumVar<-length(Betas_select2)


N<-1000 #number of models produced  (both permuted (random) and real) - for all univariate (Each featur
#Real program runs with 1000 - Takes 12 hours.

multipleAUC<-matrix(rnorm(2),1,N)
multipleAUCR<-matrix(rnorm(2),1,N)
multipleAUCNB<-matrix(rnorm(2),1,N)
multipleAUCNBR<-matrix(rnorm(2),1,N)

multipleROC<-matrix(as.list(rnorm(2)),1,N)
multipleROCR<-matrix(as.list(rnorm(2)),1,N)
multipleNBROC<-matrix(as.list(rnorm(2)),1,N)
multipleNBROCR<-matrix(as.list(rnorm(2)),1,N)

singleROC<-list()
doubleROC<-list()
singleROCR<-list()
doubleROCR<-list()

doublePlus<-list()
singlePlus<-list()
```

```r
singleAUC<-matrix(rnorm(2),NumVar-1,N)
doubleAUC<-matrix(rnorm(2),(NumVar-1),N)
singleAUCR<-matrix(rnorm(2),NumVar-1,N)
doubleAUCR<-matrix(rnorm(2),(NumVar-1),N)

doubleAUCSVMR<-matrix(rnorm(2),(NumVar-1),N)
doubleAUCSVM<-matrix(rnorm(2),(NumVar-1),N)
doubleAUCSVMCrossR<-matrix(rnorm(2),(NumVar-1),N)
doubleAUCSVMCross<-matrix(rnorm(2),(NumVar-1),N)
doubleAUCRFCross<-matrix(rnorm(2),(NumVar-1),N)
doubleAUCRFCrossR<-matrix(rnorm(2),(NumVar-1),N)
doubleAUCNBR<-matrix(rnorm(2),(NumVar-1),N)
doubleAUCNB<-matrix(rnorm(2),(NumVar-1),N)

MatsingleROC<-matrix(as.list(rnorm(2)),NumVar-1,N)
MatsingleROCR<-matrix(as.list(rnorm(2)),NumVar-1,N)
MatdoubleROC<-matrix(as.list(rnorm(2)),(NumVar-1),N)
MatdoubleROCR<-matrix(as.list(rnorm(2)),(NumVar-1),N)

MatsinglePlus<-matrix(as.list(rnorm(2)),NumVar-1,N)
MatdoublePlus<-matrix(as.list(rnorm(2)),(NumVar-1),N)
MatsinglePlusR<-matrix(as.list(rnorm(2)),NumVar-1,N)
MatdoublePlusR<-matrix(as.list(rnorm(2)),(NumVar-1),N)
##### End Block 4
```

```r
##### Begin Block 5



for (j in 1:N){ #N different measurements of AUC values, mean done at the end.
  smp_size<-floor(0.65 * nrow(Betas_select2))
  train_ind <- caret::createDataPartition(All2$Label, p = 0.65, list = FALSE, times = 1)

  # Training set
  train<-Betas_select2[train_ind, ]

  # Test set
  test<-Betas_select2[-train_ind, ]

  xtrain <- train[ , !(names(train) %in% "Label")] %>% as.matrix()

  xtest <- test[ , !(names(test) %in% "Label")] %>% as.matrix()

  ytrain<-train$Label
  ytest<-test$Label

  xtest<-data.frame(xtest)
  xtrain<-data.frame(xtrain)

  y<-Betas_select2[,NumVar]
  X<-Betas_select2[,1:(NumVar-1)]


  levels(ytrain)[1]<-"0"
```

```r
levels(ytrain)[2]<-"1"
levels(ytest)[1]<-"0"
levels(ytest)[2]<-"1"



# source('AUCFun29-3.R')

## multiple, good
multipleAUCNB[1,j]<-multipleAUCfunNB(xtrain, ytrain,xtest,ytest)
multipleAUC[1,j]<-multipleAUCfun(xtrain, ytrain,xtest,ytest)

multipleNBROC[[j]]<-multipleROCfunNB(xtrain, ytrain,xtest,ytest)
multipleROC[[j]]<-multipleROCfun(xtrain, ytrain,xtest,ytest)


#separate


## single, should be good
for (s in (1:(NumVar-1))){
  s<-as.numeric(s)
  singleAUC[s,j]<-singleAUCfun(xtrain, ytrain,xtest,ytest,s)
  singleROC[[s]]<-singleROCfun(xtrain, ytrain,xtest,ytest,s)
  MatsinglePlus[s,j]<-singlePlusfun(xtrain, ytrain,xtest,ytest,s)
}
MatsingleROC[,j]<-matrix(singleROC)






# Training set

train$Label<-sample(train$Label)
test$Label<-sample(test$Label)
#Permuted data, will make sure that are models are really valid as randomizing the label should yield

# Test set

xtrain <- train[ , !(names(train) %in% "Label")] %>% as.matrix()

xtest <- test[ , !(names(test) %in% "Label")] %>% as.matrix()

ytrain<-train$Label
ytest<-test$Label

xtest<-data.frame(xtest)
xtrain<-data.frame(xtrain)


y<-Betas_select2[,NumVar]
X<-Betas_select2[,1:(NumVar-1)]
```

```
  levels(ytrain)[1]<-"0"
  levels(ytrain)[2]<-"1"
  levels(ytest)[1]<-"0"
  levels(ytest)[2]<-"1"




  multipleAUCNBR[1,j]<-multipleAUCfunNB(xtrain, ytrain,xtest,ytest)
  multipleAUCR[1,j]<-multipleAUCfun(xtrain, ytrain,xtest,ytest)

  multipleNBROCR[[j]]<-multipleROCfunNB(xtrain, ytrain,xtest,ytest)
  multipleROCR[[j]]<-multipleROCfun(xtrain, ytrain,xtest,ytest)


  for (s in (1:(NumVar-1))){
    s<-as.numeric(s)
    singleAUCR[s,j]<-singleAUCfun(xtrain, ytrain,xtest,ytest,s)
    singleROCR[[s]]<-singleROCfun(xtrain, ytrain,xtest,ytest,s)
    MatsinglePlusR[s,j]<-singlePlusfun(xtrain, ytrain,xtest,ytest,s)
  }
  MatsingleROCR[,j]<-matrix(singleROCR)


  #
}

##### End Block 5
```

```
##### Begin Block 6


  names2<-sapply(1:(NumVar-1), function(i){paste0("NISS/",names(xtrain)[i])})


trial<-NULL

h=1
for (b in (2:NumVar-1)) {
  print(b)
  trial[b]<-names2[h]
  h=h+1
}
```

```
## [1] 1
## [1] 2
## [1] 3
## [1] 4
## [1] 5
## [1] 6
## [1] 7
## [1] 8
## [1] 9
```

```
## [1] 10
## [1] 11
## [1] 12
## [1] 13
## [1] 14
```

```
singleAUC<-as.data.frame(singleAUC)
singleAUC<-mutate(singleAUC, Means=rowMeans(singleAUC))
row.names(singleAUC)<-names(xtrain)

singleAUCR<-as.data.frame(singleAUCR)
singleAUCR<-mutate(singleAUCR, Means=rowMeans(singleAUCR))
row.names(singleAUCR)<-names(xtrain)

multipleAUCNBR<-as.data.frame(multipleAUCNBR)
multipleAUCNBR["Means"]<-rowMeans(multipleAUCNBR)
multipleAUCR<-as.data.frame(multipleAUCR)
multipleAUCR["Means"]<-rowMeans(as.data.frame(multipleAUCR))

multipleAUCNB<-as.data.frame(multipleAUCNB)
multipleAUCNB["Means"]<-rowMeans(as.data.frame(multipleAUCNB))
multipleAUC<-as.data.frame(multipleAUC)
multipleAUC["Means"]<-rowMeans(as.data.frame(multipleAUC))

Final<-data.frame(MultiNB=t(multipleAUCNB),MultiNBRand=t(multipleAUCNBR),Multi=t(multipleAUC),MultiRand[illegible]
FinalMeans<-data.frame(MultiNB=multipleAUCNB$Means,MultiNBRand=multipleAUCNBR$Means,Multi=multipleAUC$M[illegible]

#sacar ROC CURVES #####

print("here")
```

```
## [1] "here"
```

```
for (g in 1:(NumVar-1)){

  plot(MatsingleROC[[g,1]],lwd=3,main=paste("ROC curve of", names(xtrain)[g]))
  for (b in 1:N){

    plot(MatsingleROCR[[g,b]],col=b,lty=3,add=TRUE)
  }
}
```

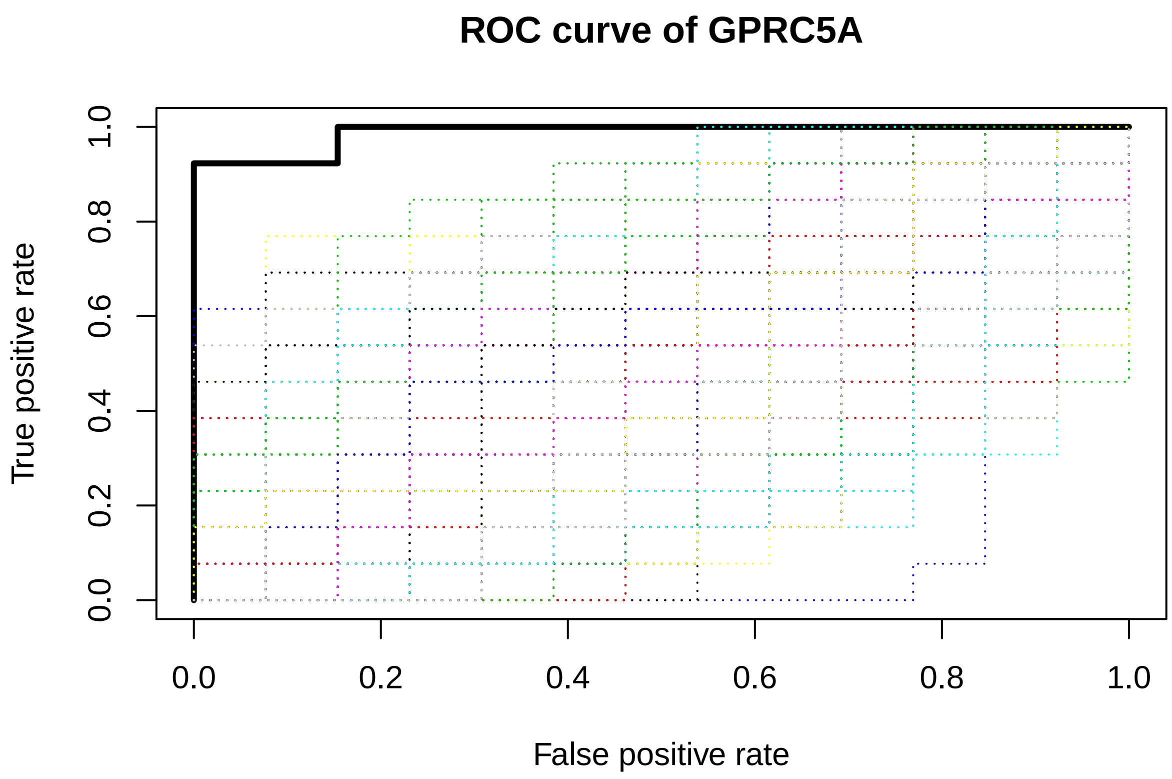
ROC curve of GPRC5A
True positive rate
False positive rate
0.0
0.2
0.4
0.6
0.8
1.0
0.0
0.2
0.4
0.6
0.8
1.0

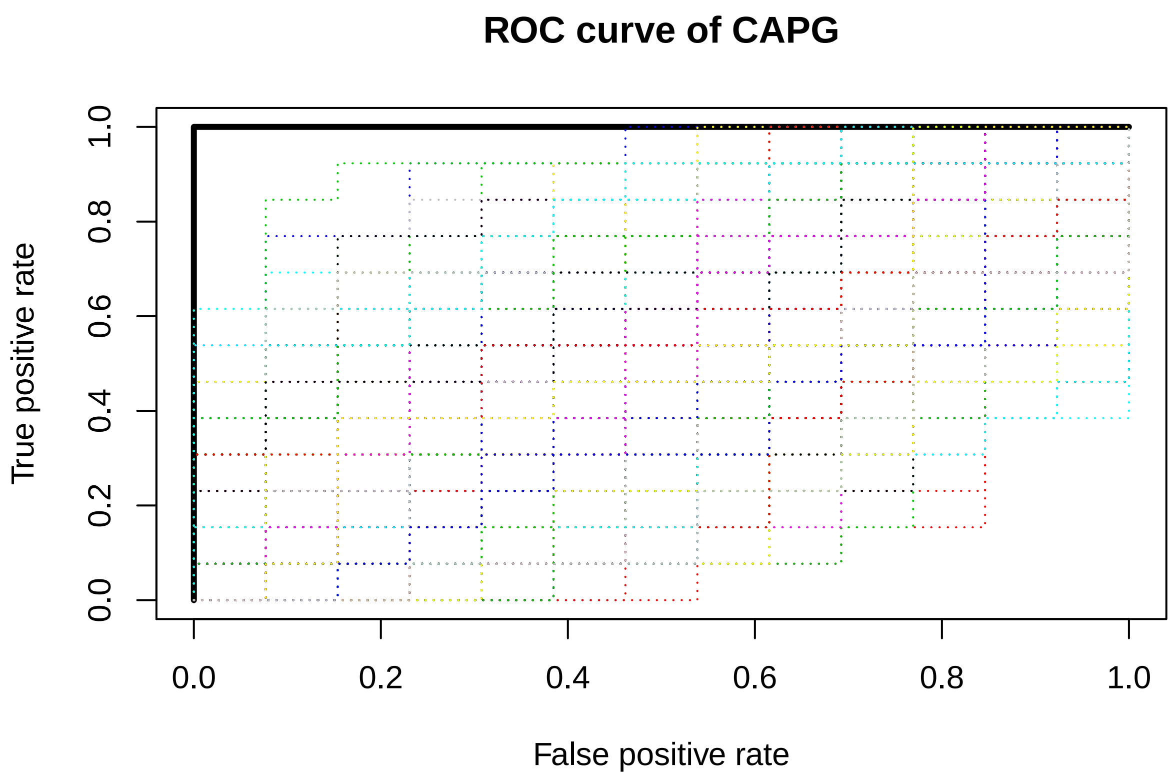
ROC curve of CAPG
True positive rate
0.0
0.2
0.4
0.6
0.8
1.0
False positive rate
0.0
0.2
0.4
0.6
0.8
1.0

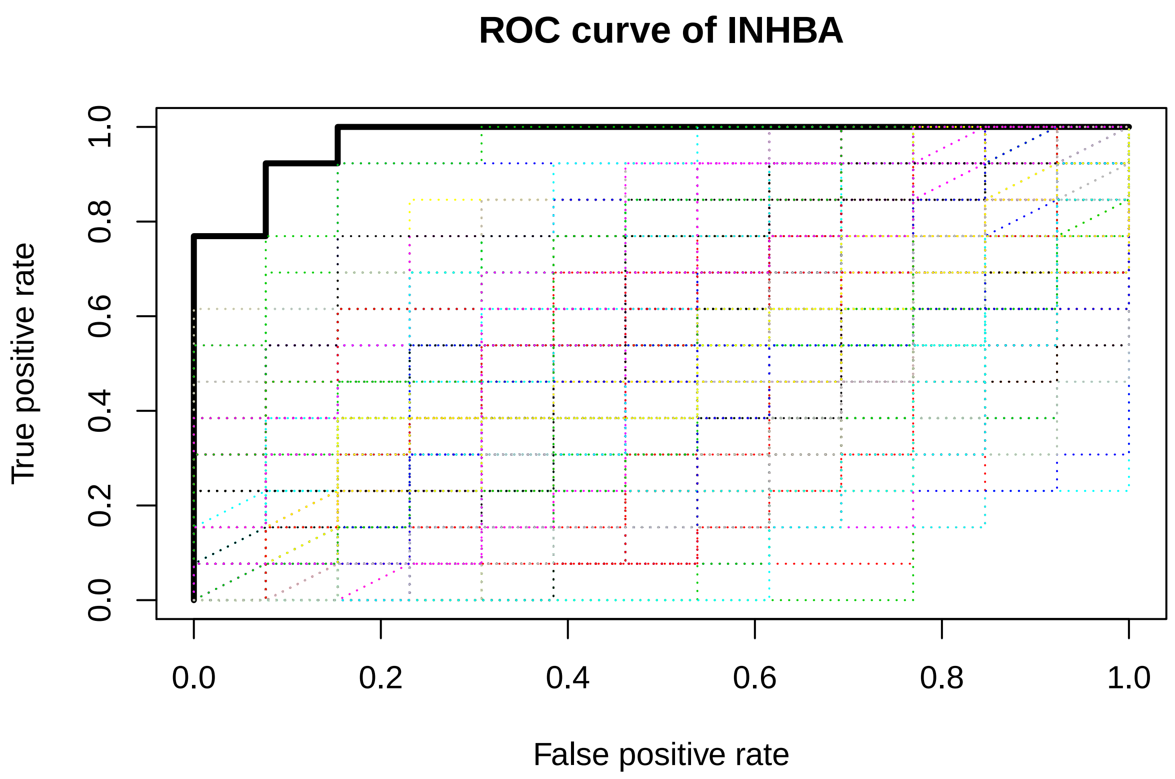
ROC curve of INHBA
True positive rate
False positive rate
0.0
0.2
0.4
0.6
0.8
1.0

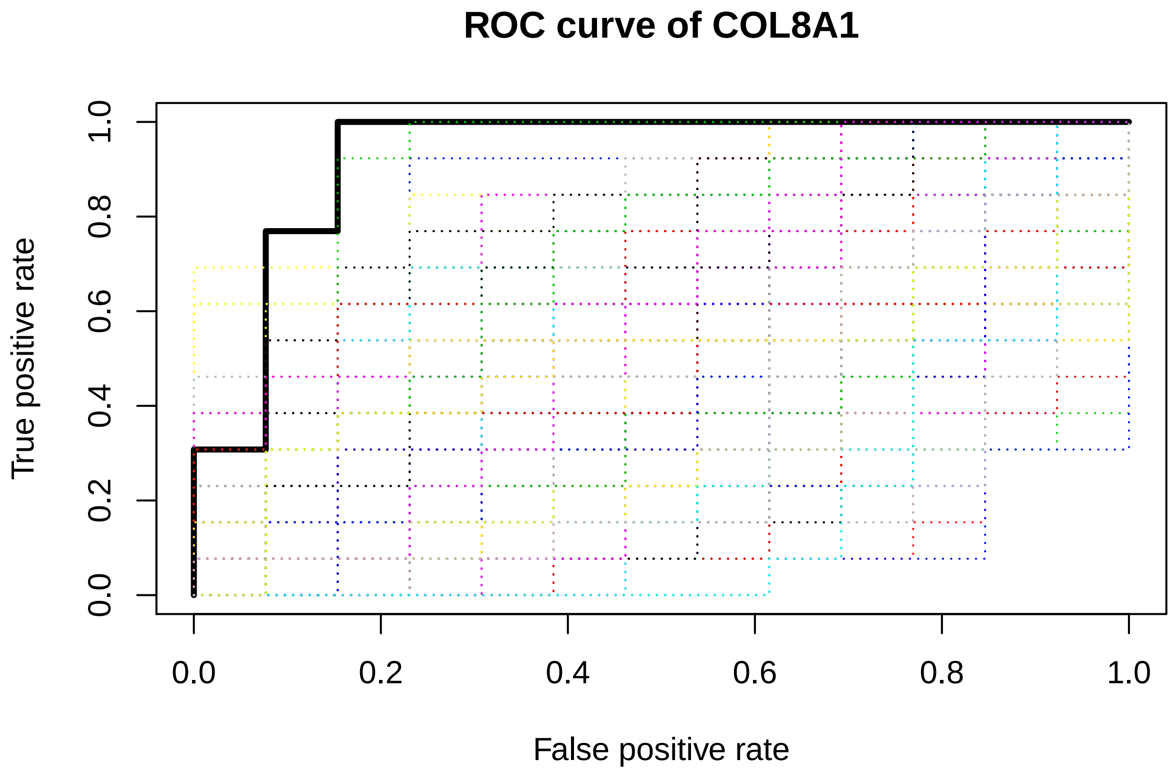
ROC curve of COL8A1
True positive rate
0.0
0.2
0.4
0.6
0.8
1.0
False positive rate
0.0
0.2
0.4
0.6
0.8
1.0

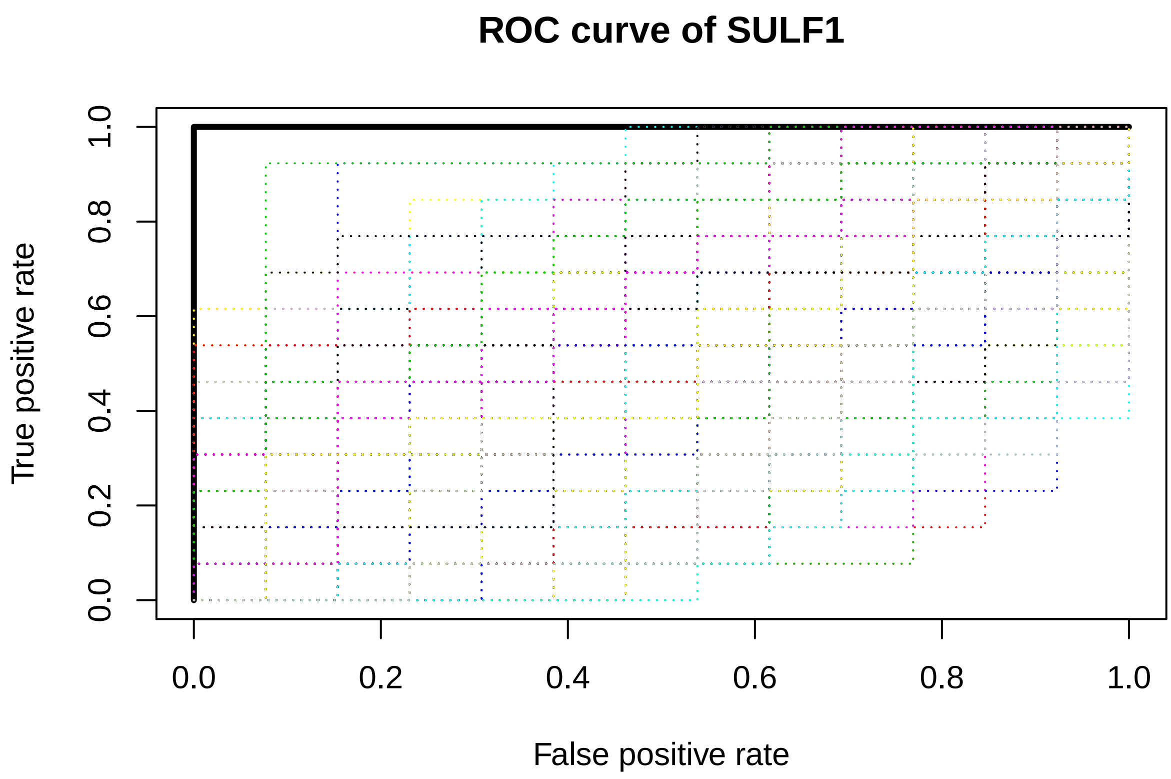
ROC curve of SULF1
True positive rate
False positive rate
0.0
0.2
0.4
0.6
0.8
1.0

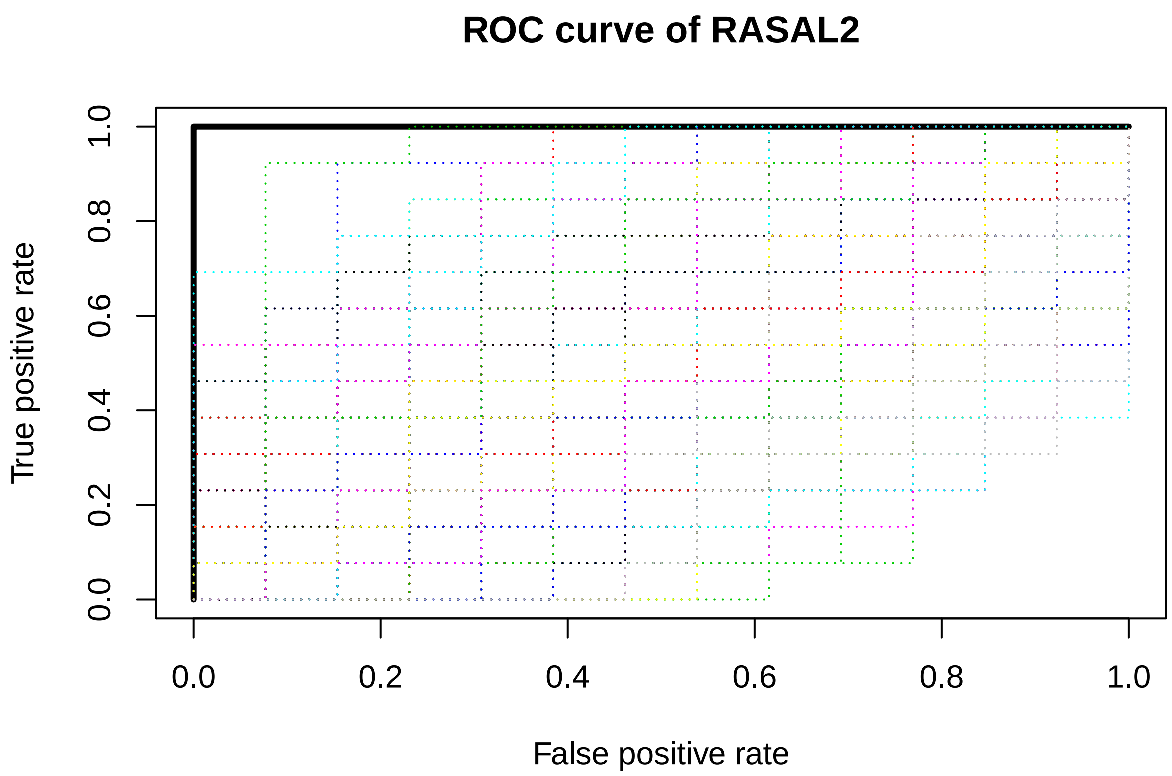
ROC curve of RASAL2
True positive rate
0.0
0.2
0.4
0.6
0.8
1.0
False positive rate
0.0
0.2
0.4
0.6
0.8
1.0

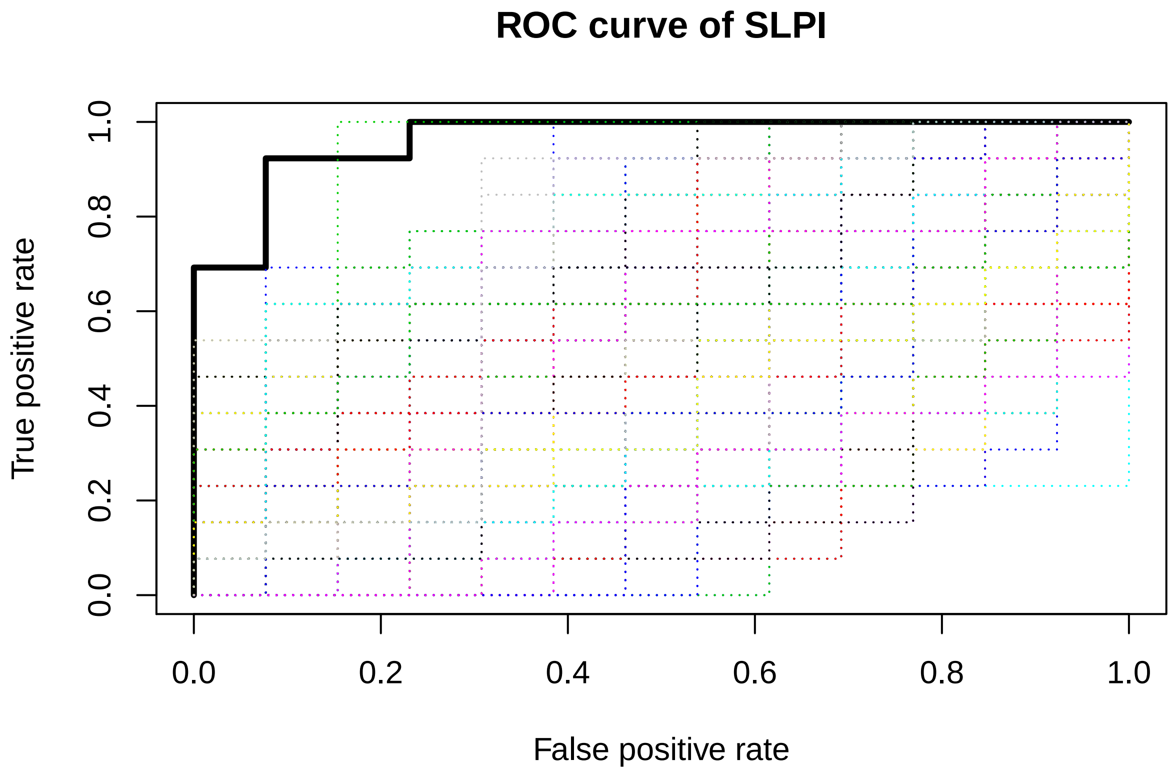

ROC curve of SLPI
True positive rate
False positive rate
0.0
0.2
0.4
0.6
0.8
1.0

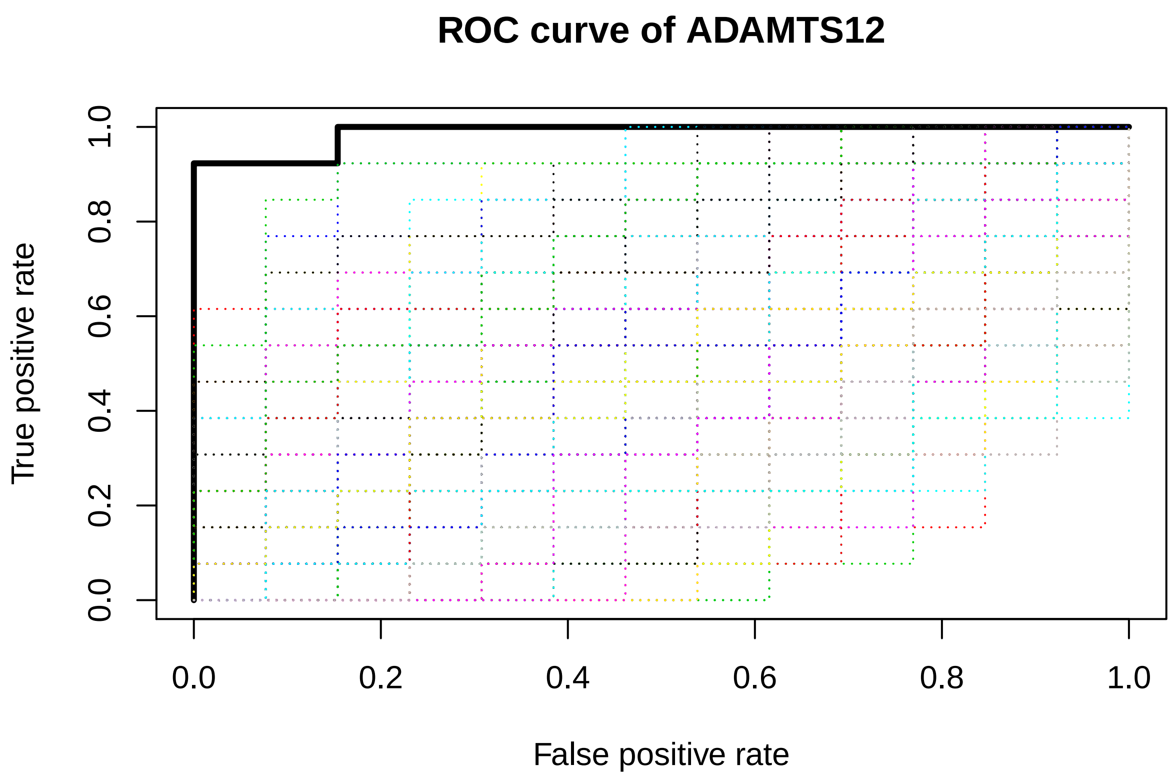
ROC curve of ADAMTS12
True positive rate
False positive rate
0.0
0.2
0.4
0.6
0.8
1.0

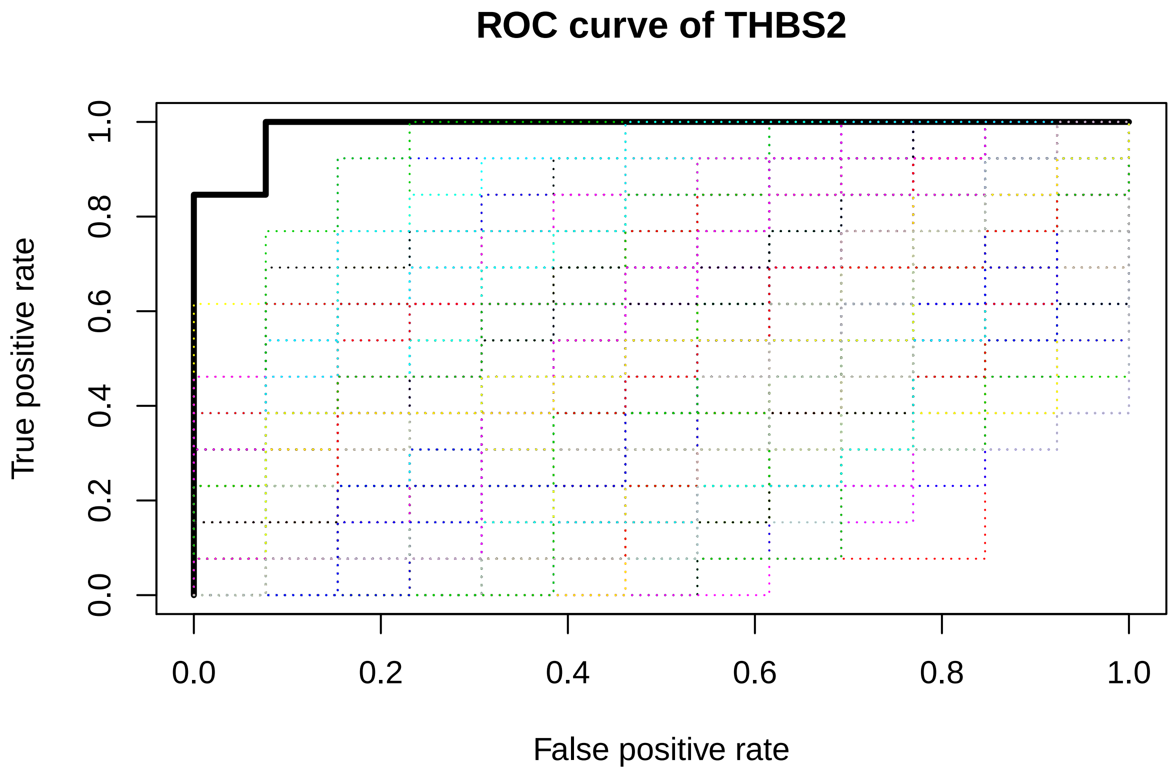

ROC curve of THBS2
True positive rate
False positive rate
0.0
0.2
0.4
0.6
0.8
1.0
0.0
0.2
0.4
0.6
0.8
1.0

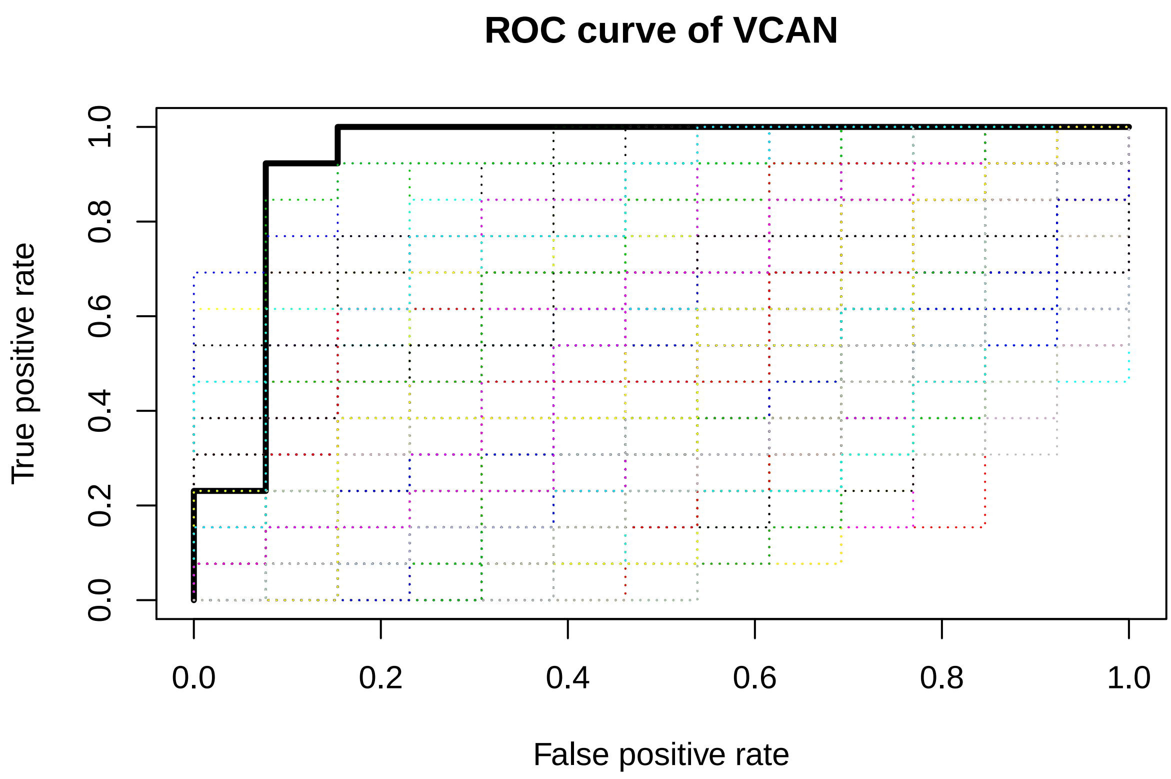
ROC curve of VCAN
True positive rate
0.0
0.2
0.4
0.6
0.8
1.0
False positive rate
0.0
0.2
0.4
0.6
0.8
1.0

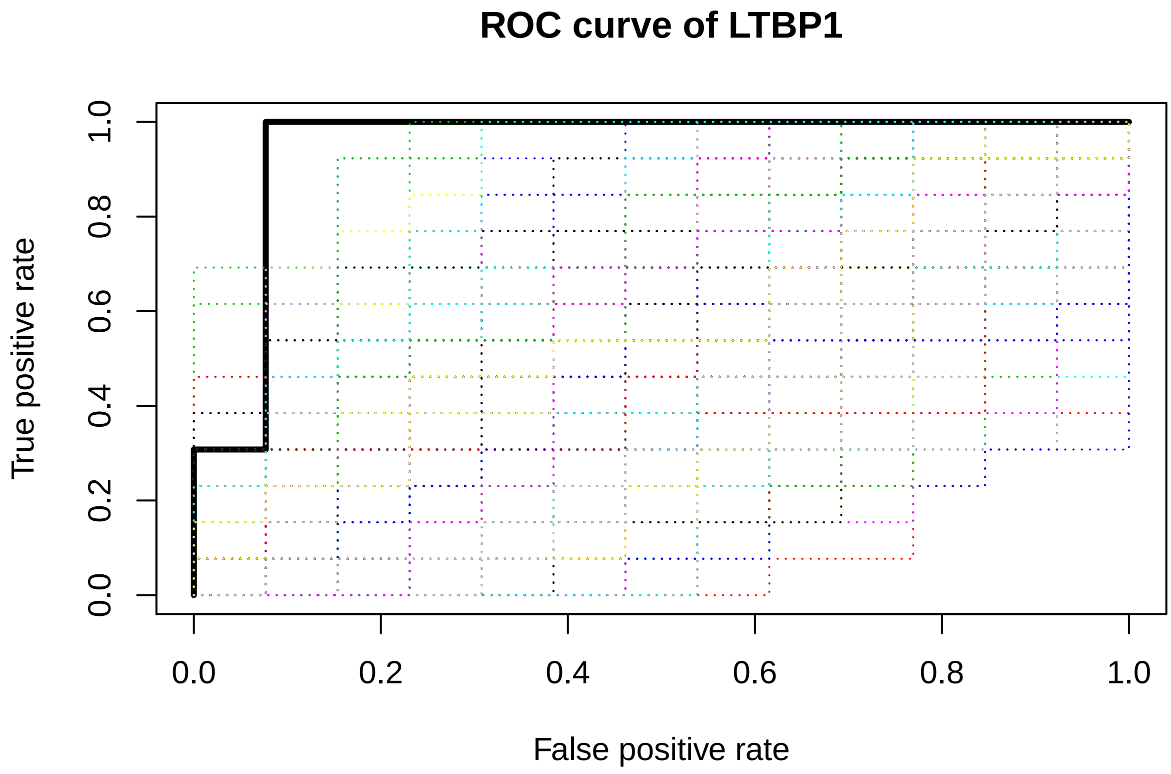

ROC curve of LTBP1
True positive rate
False positive rate
0.0
0.2
0.4
0.6
0.8
1.0

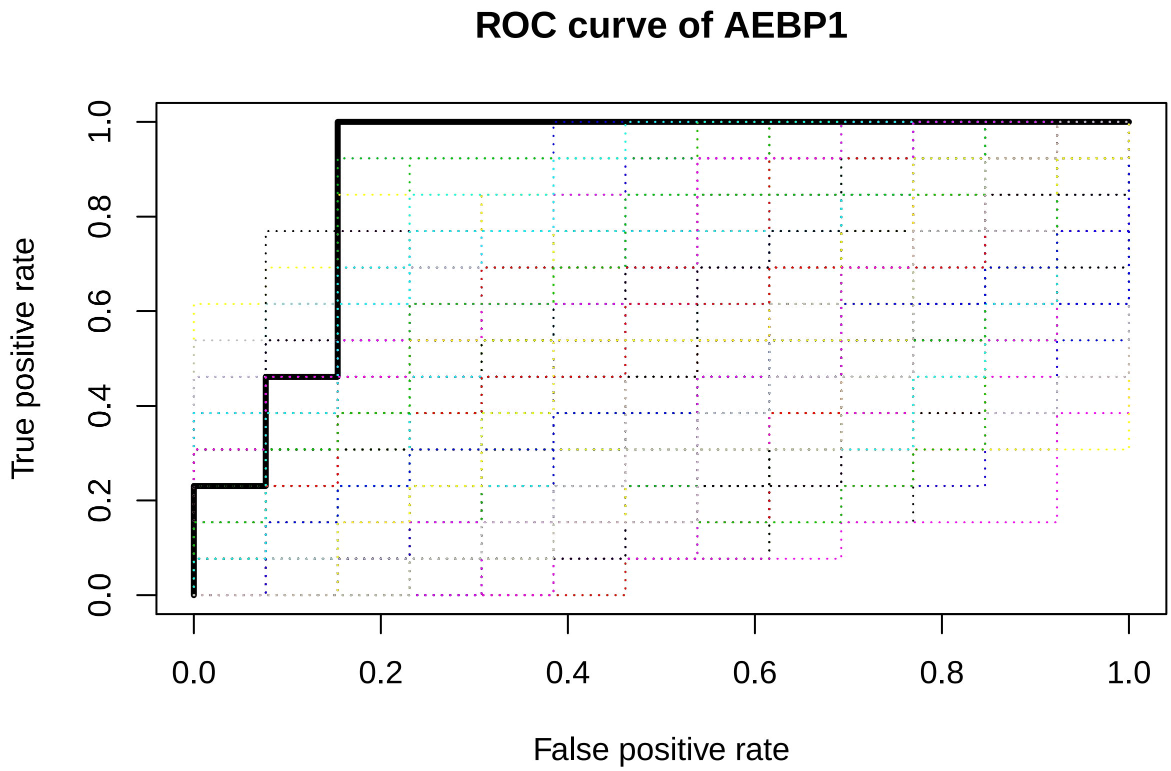

ROC curve of AEBP1
True positive rate
False positive rate
0.0
0.2
0.4
0.6
0.8
1.0

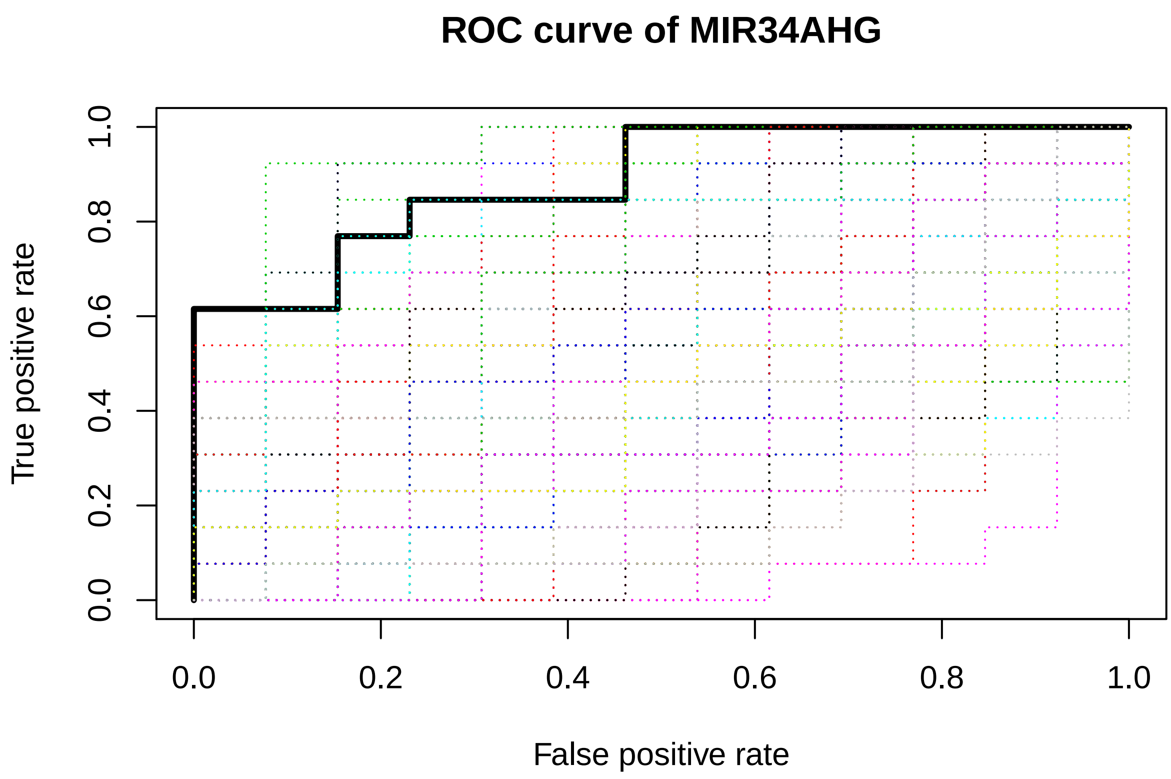
ROC curve of MIR34AHG
True positive rate
False positive rate
0.0
0.2
0.4
0.6
0.8
1.0

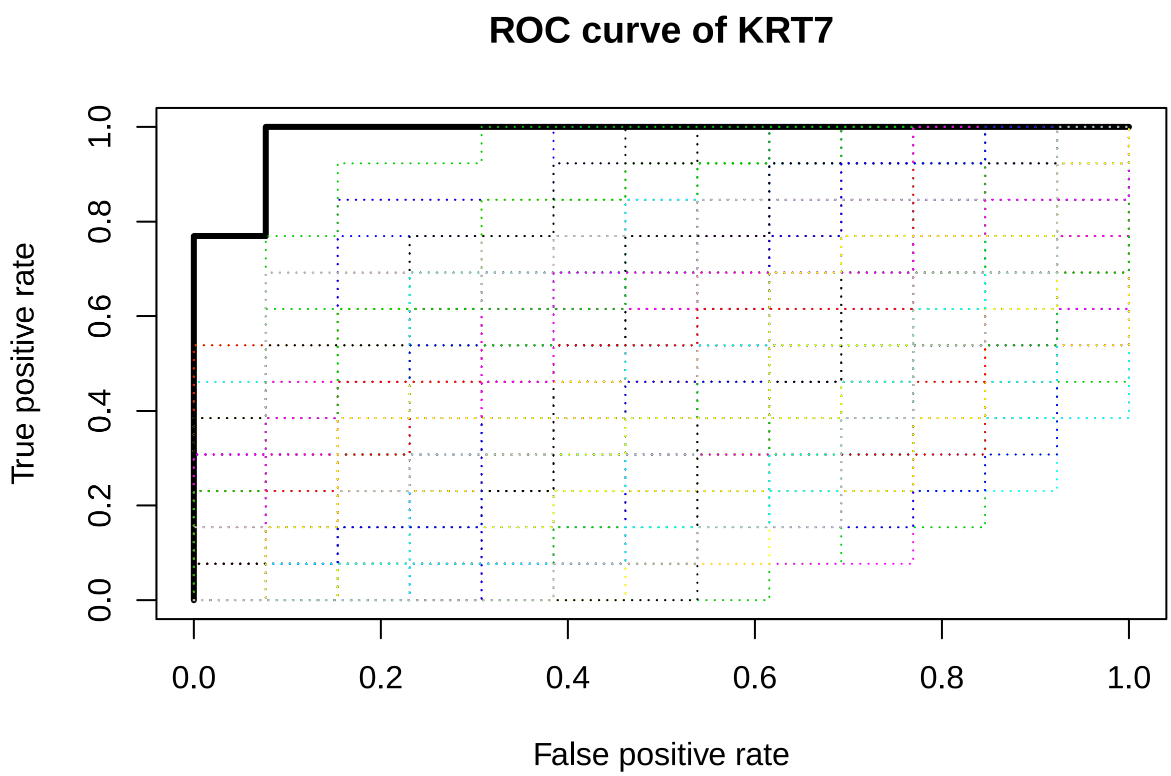


```
plot(multipleROC[[1]],lwd=3,main=paste("ROC curve of", trial[1]))
for (b in 1:N){
  plot(multipleNBROCR[[b]],col=b,lty=3,add=TRUE)
}
```

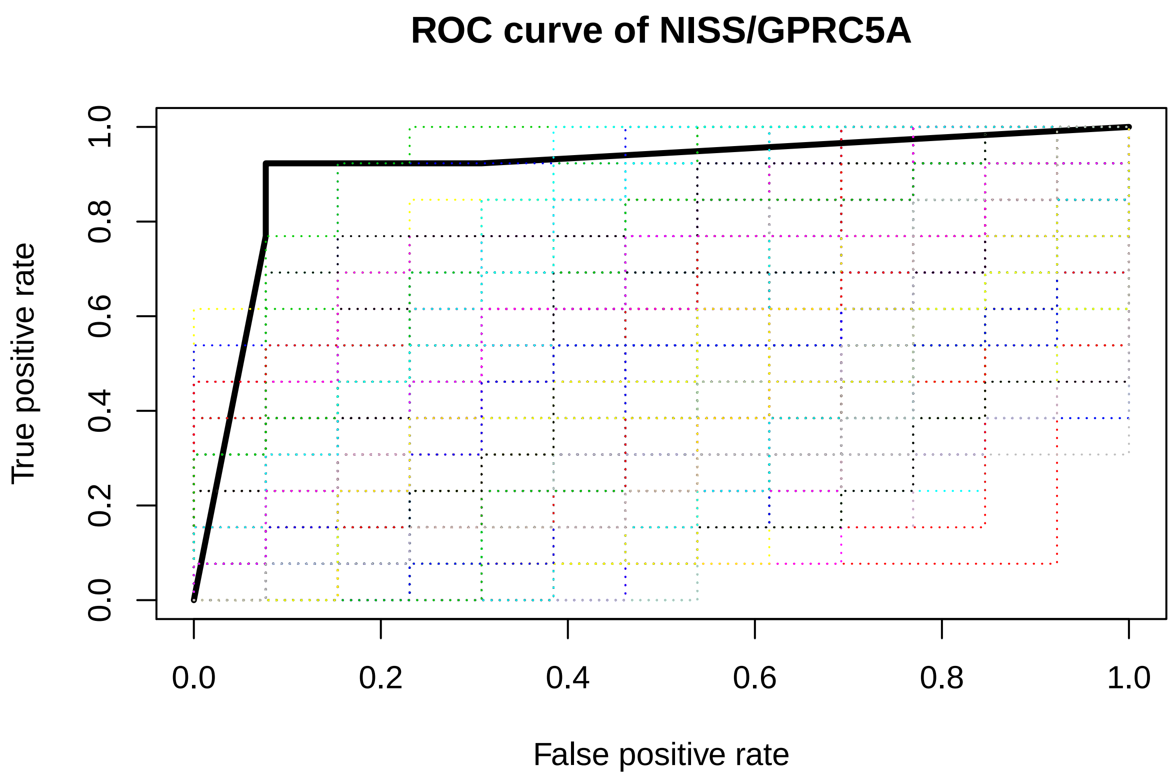


```r
AUCT<-as.data.frame(t(singleAUC[1:dim(singleAUC)[2]-1]))
AUC2T<-as.data.frame(t(singleAUCR[1:dim(singleAUCR)[2]-1]))
one<-as.data.frame(singleAUC$Means)
two<-as.data.frame(singleAUCR$Means)
Mean<-data.frame(one,two)
Mean<-as.matrix(Mean)
#
nm<-names(AUCT)
for (j in 1:(NumVar-1)){

  Mono<-data.frame(Mono=AUCT[,j],Label=as.factor(c(rep("model",dim(AUCT)[1]))))
  Mono1<-data.frame(Mono=AUC2T[,j],Label=as.factor(c(rep("permuted data",dim(AUC2T)[1]))))
  Mono<-rbind(Mono,Mono1)
  print(ggdensity(Mono, x = "Mono", fill = "Label", palette = "jco")+geom_vline(xintercept =Mean[j,],li
}
```

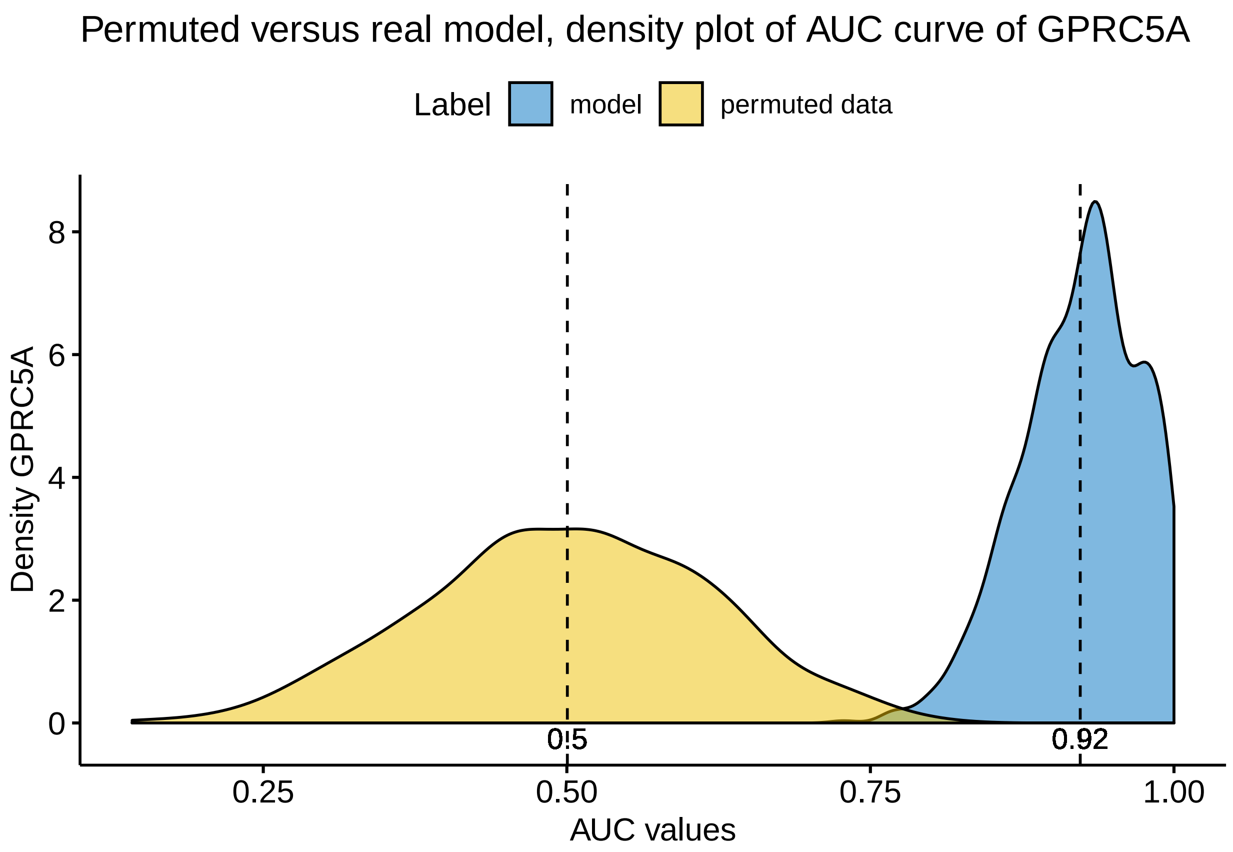
Permuted versus real model, density plot of AUC curve of GPRC5A
Label
model
permuted data
Density GPRC5A
0
2
4
6
8
0.5
0.92
0.25
0.50
0.75
1.00
AUC values

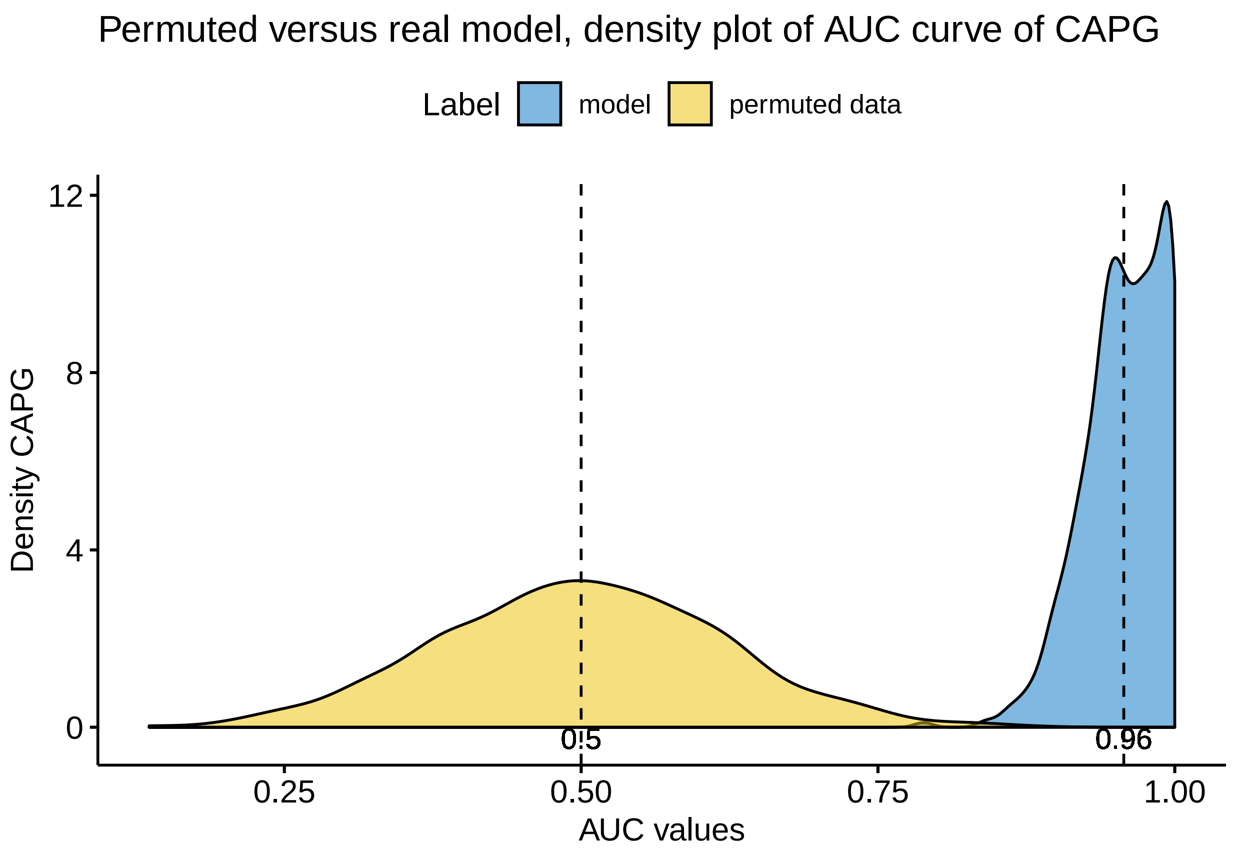
Permuted versus real model, density plot of AUC curve of CAPG
Label
model
permuted data
Density CAPG
0
4
8
12
0.5
0.96
0.25
0.50
0.75
1.00
AUC values

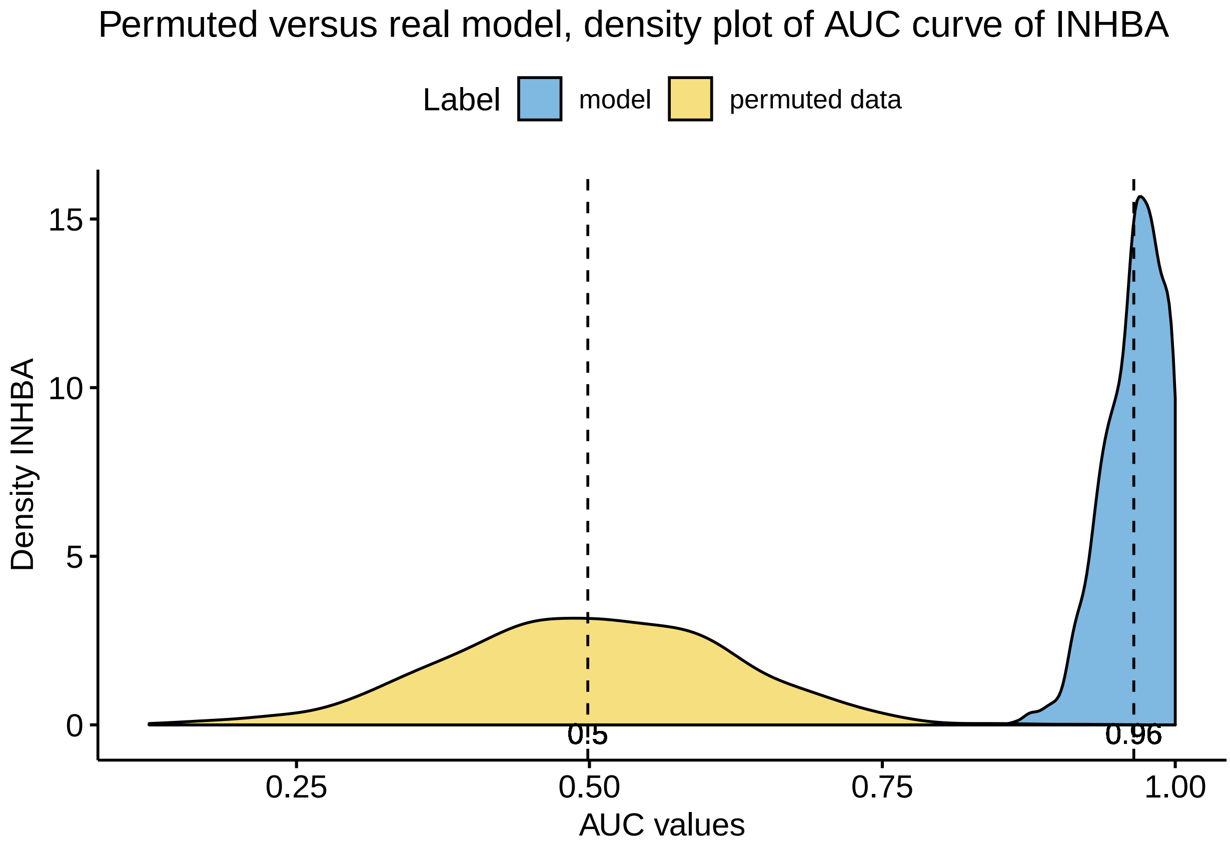
Permuted versus real model, density plot of AUC curve of INHBA
Label
model
permuted data
Density INHBA
15
10
5
0
0.5
0.96
0.25
0.50
0.75
1.00
AUC values

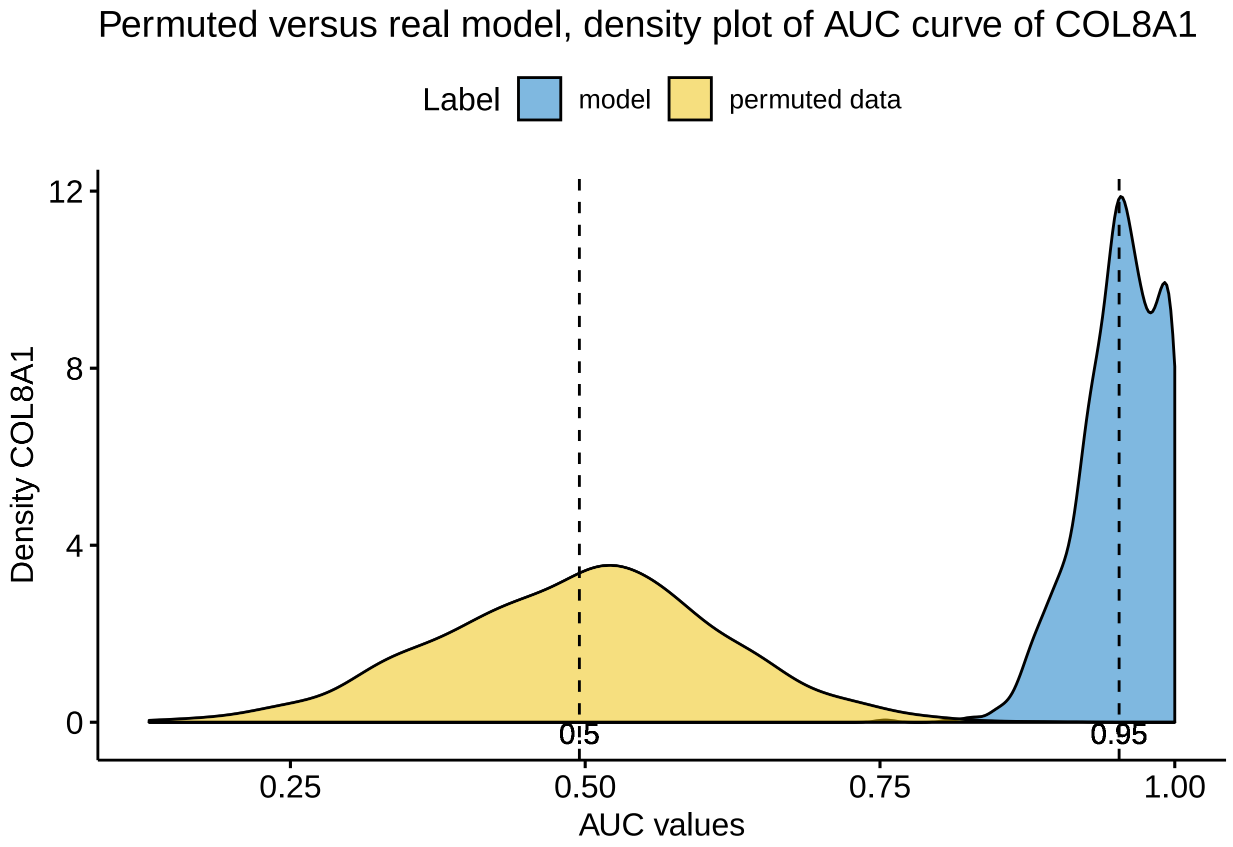

Permuted versus real model, density plot of AUC curve of COL8A1
Label
model
permuted data
Density COL8A1
12
8
4
0
0.5
0.95
0.25
0.50
0.75
1.00
AUC values

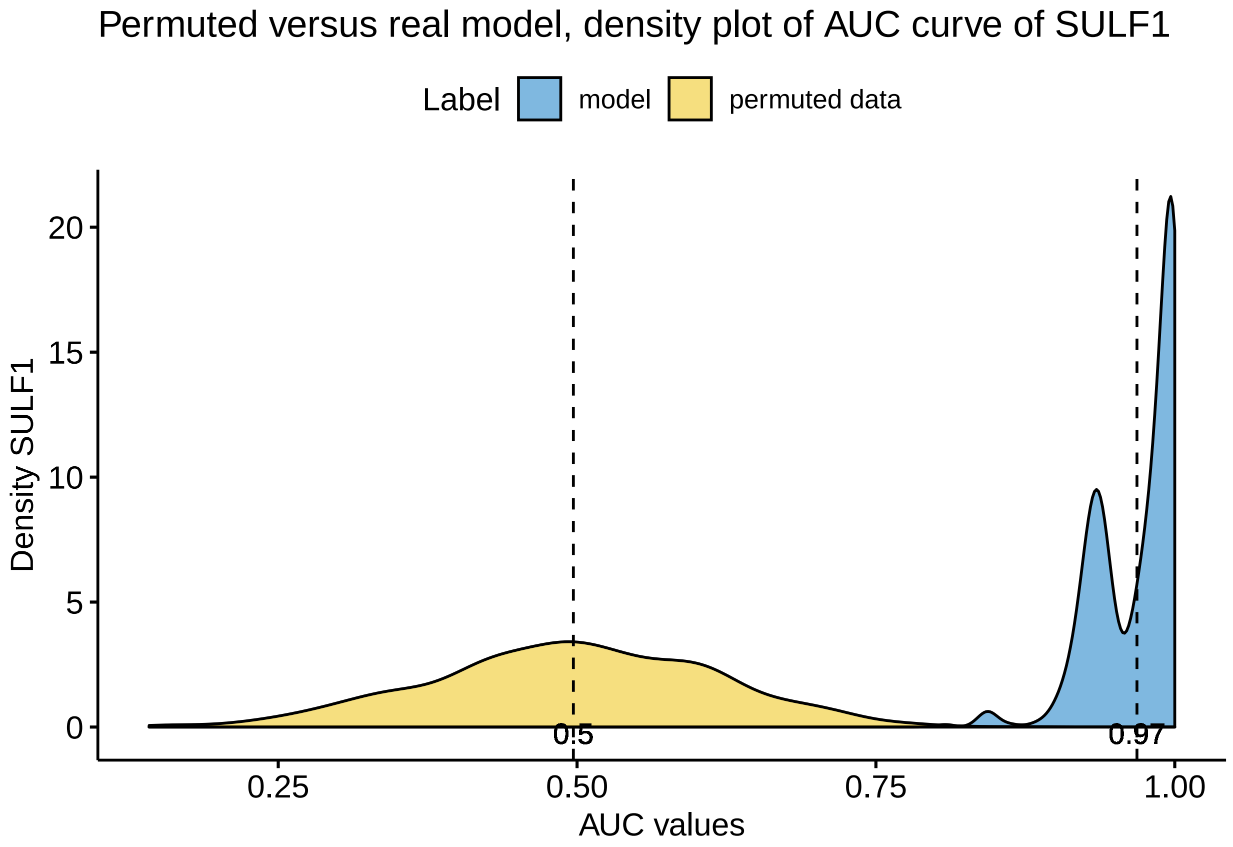

Permuted versus real model, density plot of AUC curve of SULF1
Label
model
permuted data
Density SULF1
20
15
10
5
0
0.5
0.97
0.25
0.50
0.75
1.00
AUC values

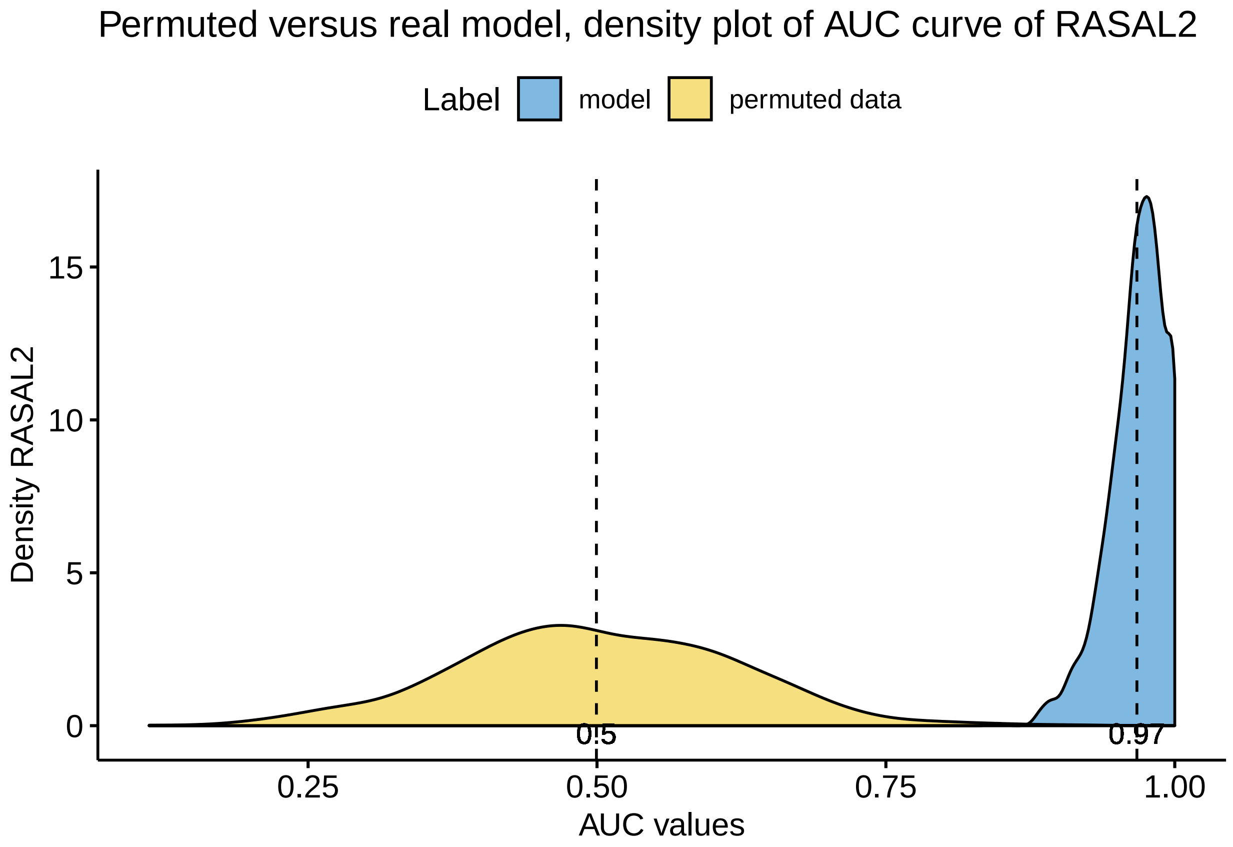

Permuted versus real model, density plot of AUC curve of RASAL2
Label
model
permuted data
Density RASAL2
15
10
5
0
0.5
0.97
0.25
0.50
0.75
1.00
AUC values

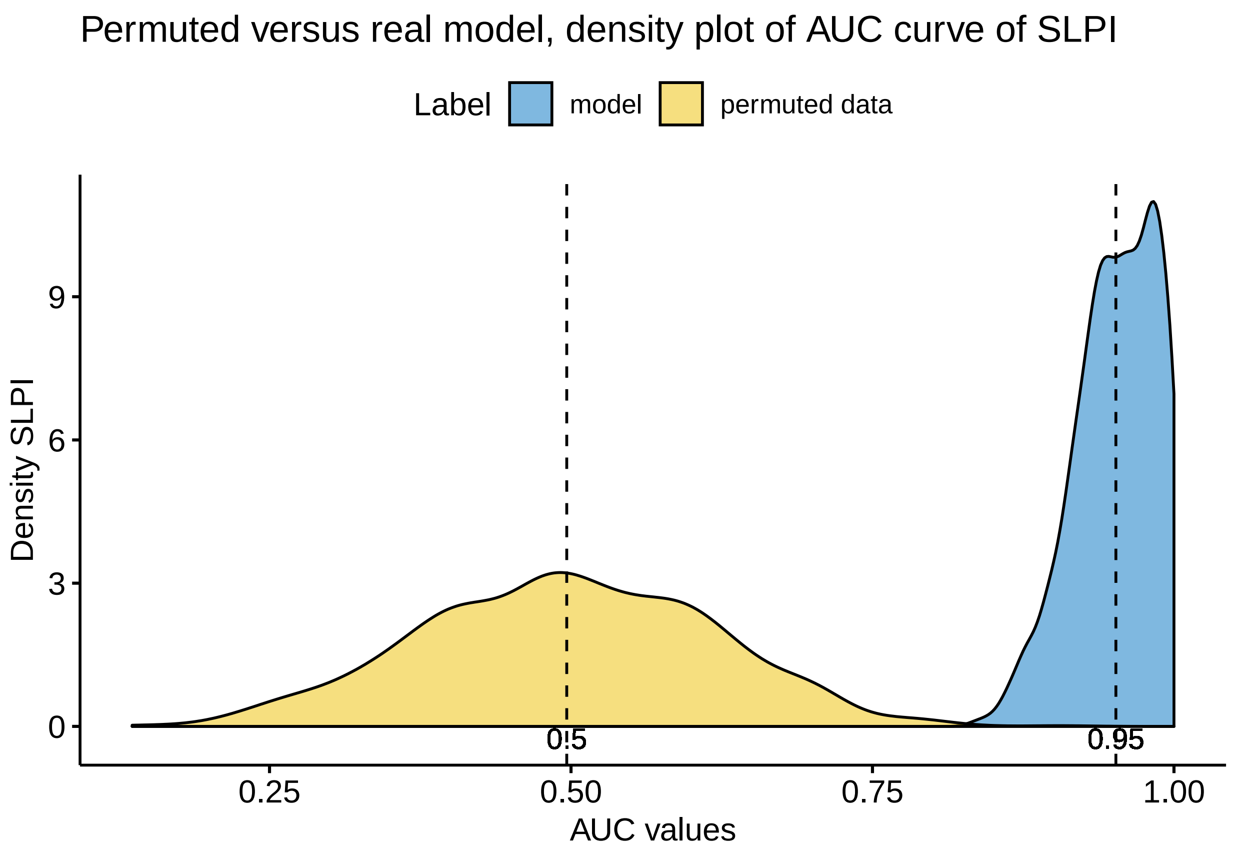

Permuted versus real model, density plot of AUC curve of SLPI
Label
model
permuted data
Density SLPI
9
6
3
0
0.5
0.95
0.25
0.50
0.75
1.00
AUC values

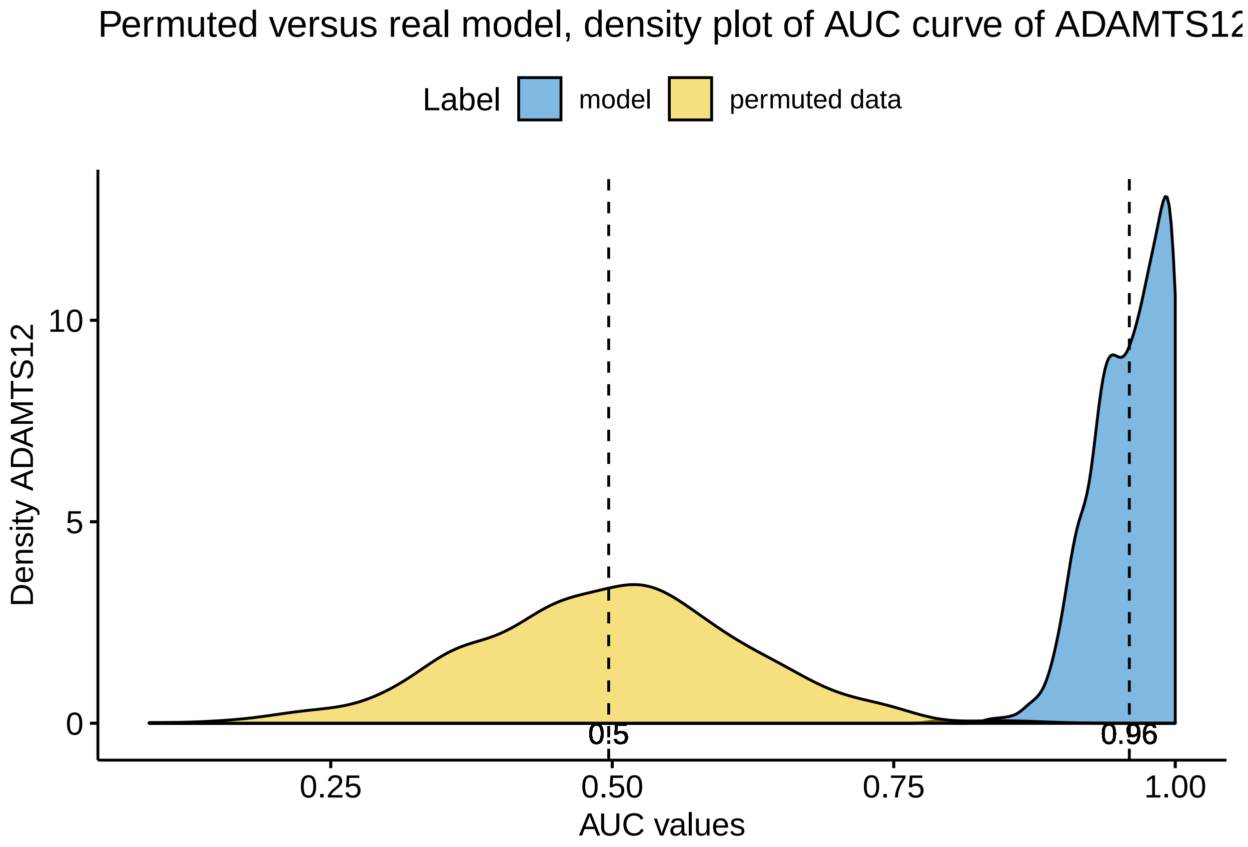
Permuted versus real model, density plot of AUC curve of ADAMTS12
Label
model
permuted data
Density ADAMTS12
10
5
0
0.5
0.96
0.25
0.50
0.75
1.00
AUC values

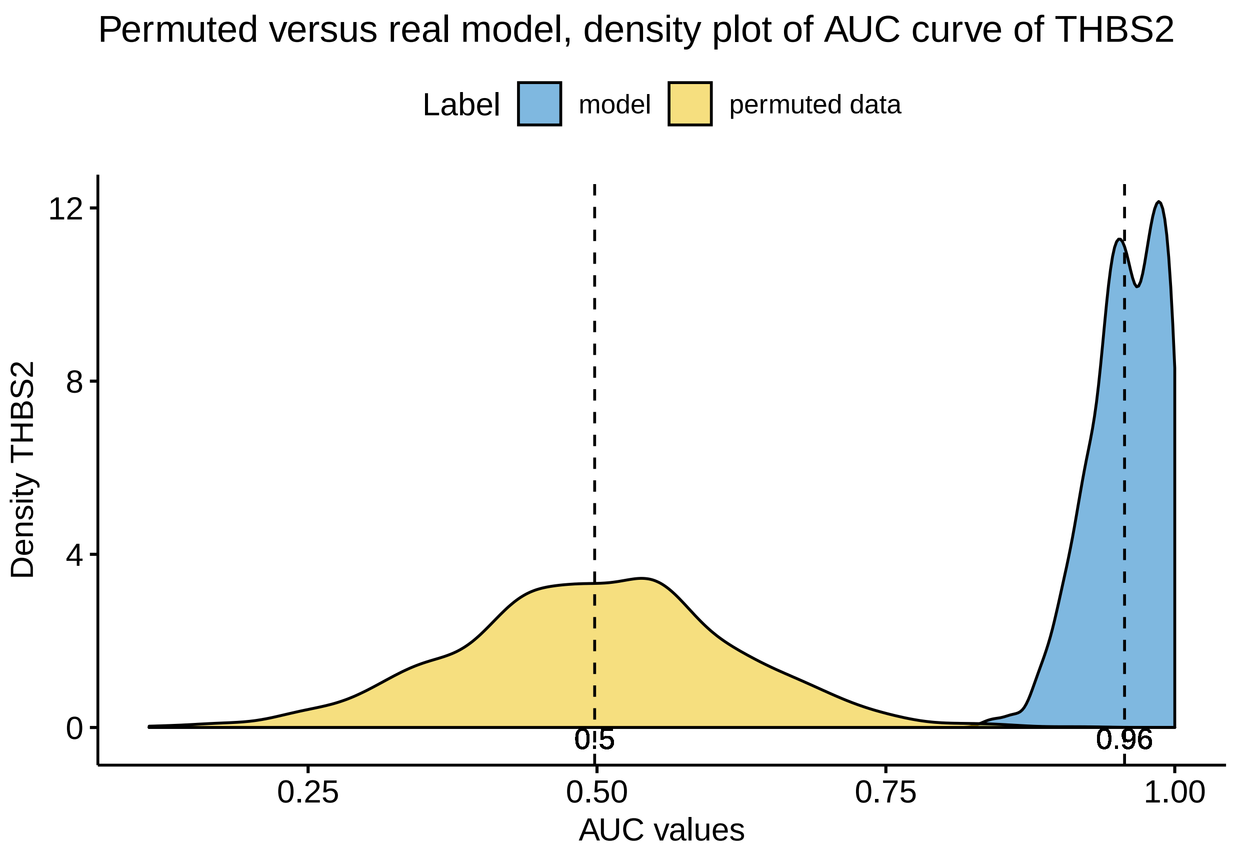
Permuted versus real model, density plot of AUC curve of THBS2
Label
model
permuted data
Density THBS2
12
8
4
0
0.5
0.96
0.25
0.50
0.75
1.00
AUC values

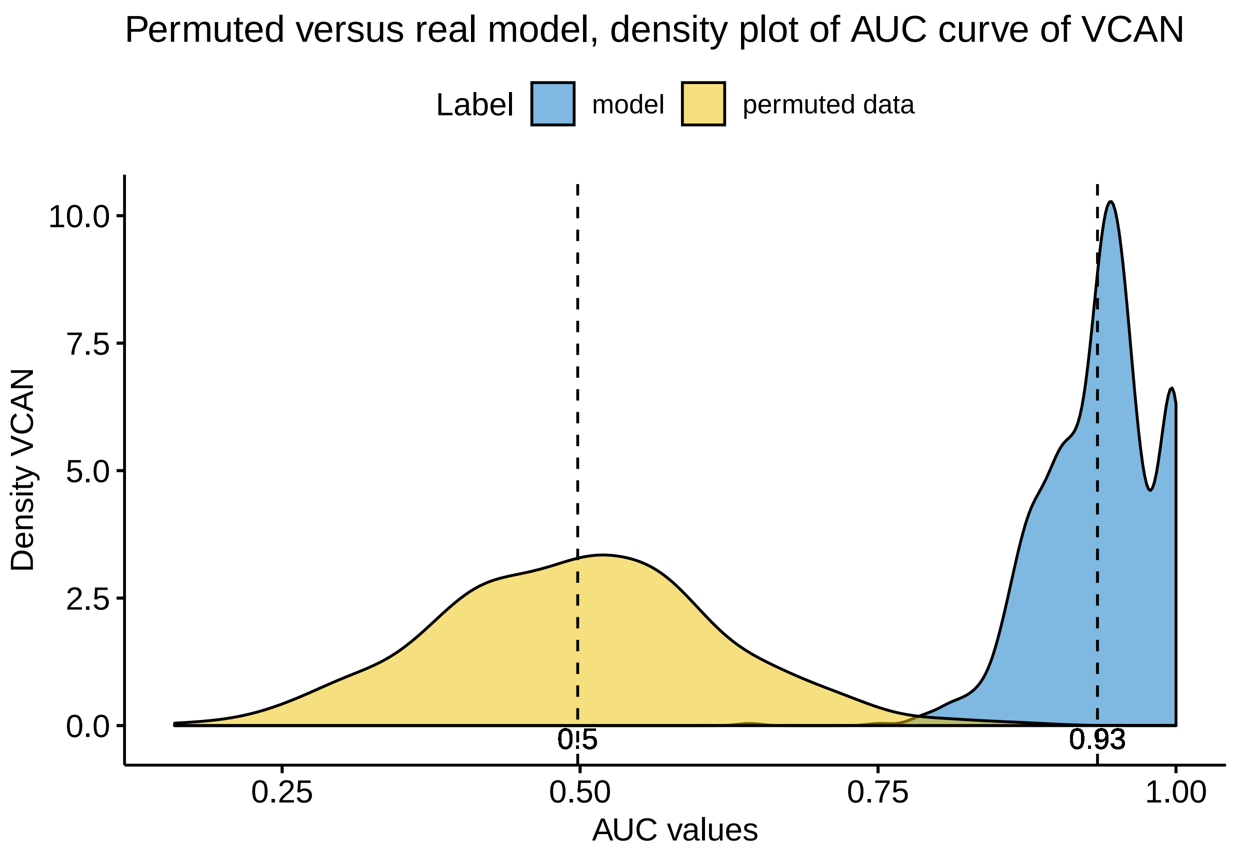
Permuted versus real model, density plot of AUC curve of VCAN
Label
model
permuted data
Density VCAN
10.0
7.5
5.0
2.5
0.0
0.5
0.93
0.25
0.50
0.75
1.00
AUC values

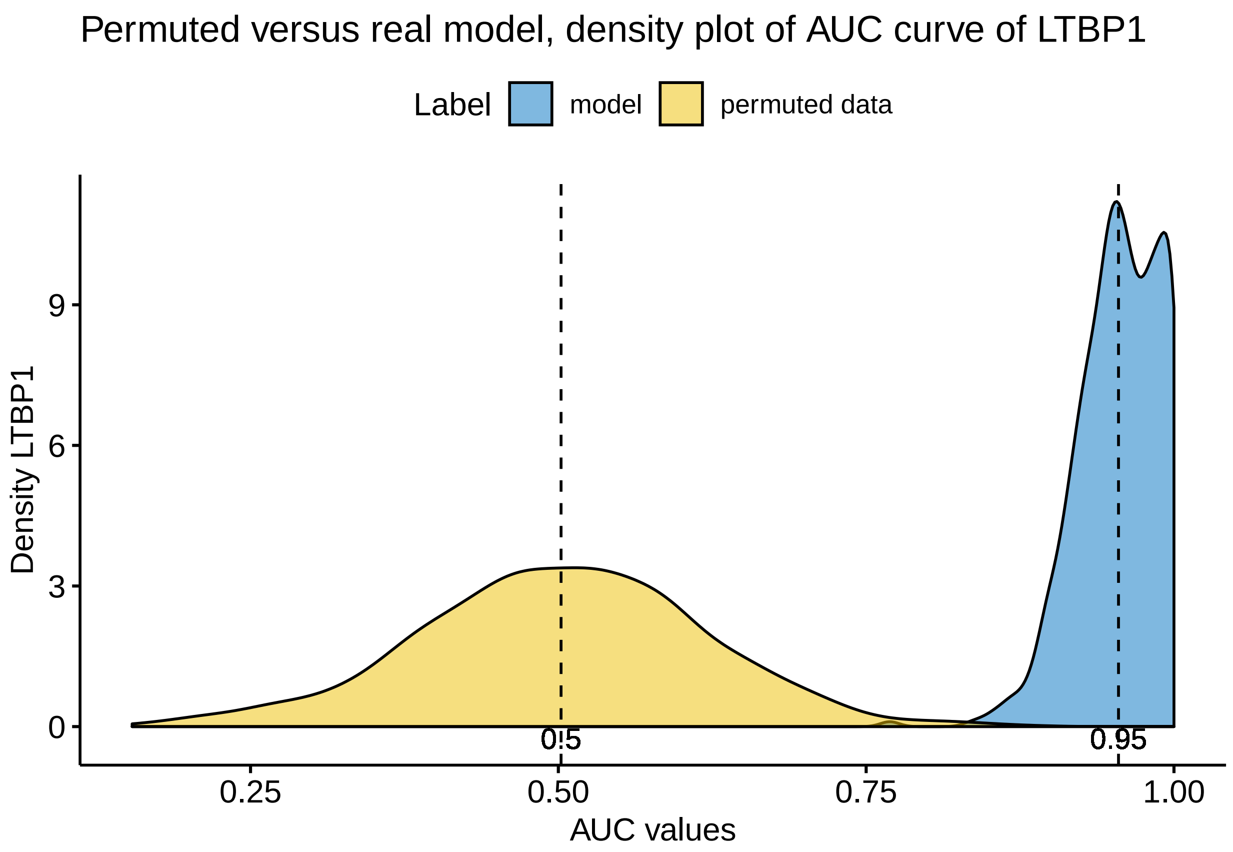

Permuted versus real model, density plot of AUC curve of LTBP1
Label
model
permuted data
Density LTBP1
9
6
3
0
0.5
0.95
0.25
0.50
0.75
1.00
AUC values

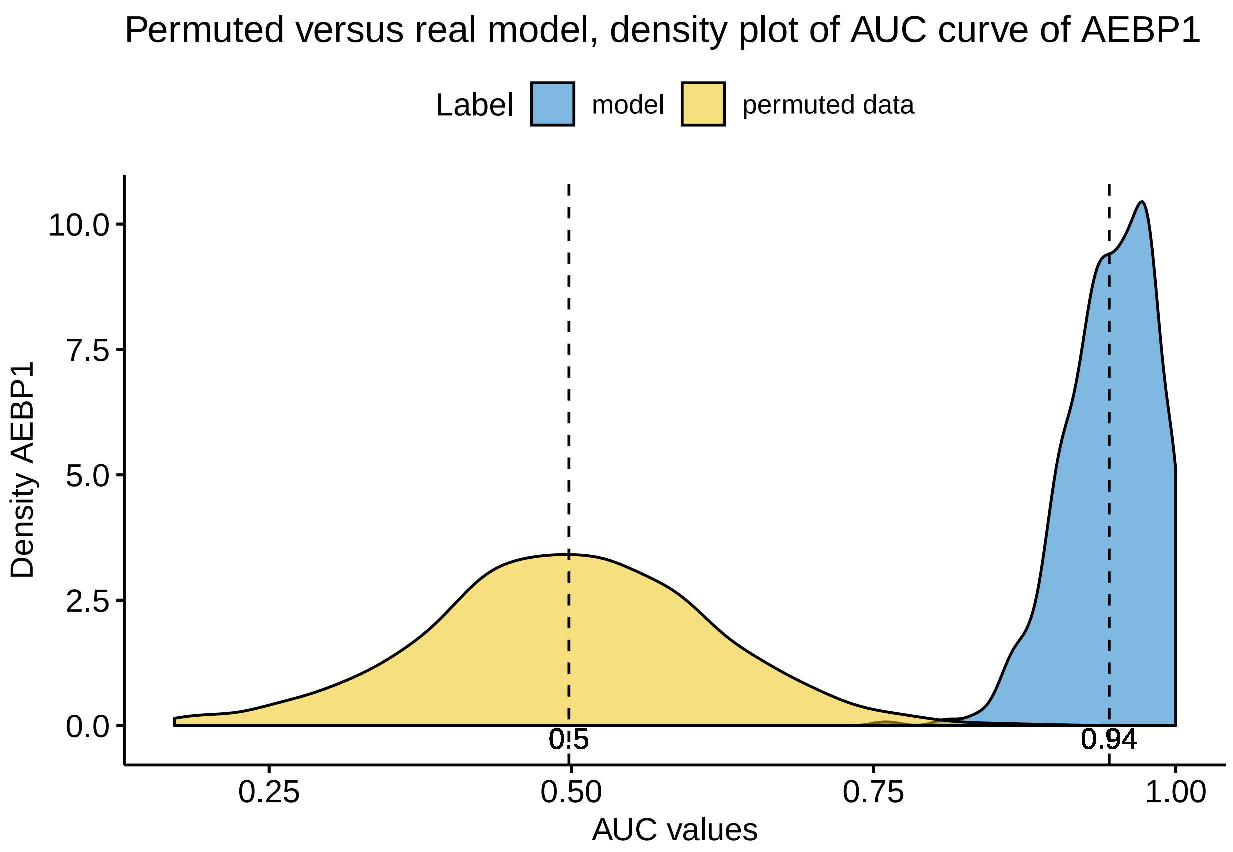

Permuted versus real model, density plot of AUC curve of AEBP1
Label
model
permuted data
Density AEBP1
10.0
7.5
5.0
2.5
0.0
0.5
0.94
0.25
0.50
0.75
1.00
AUC values

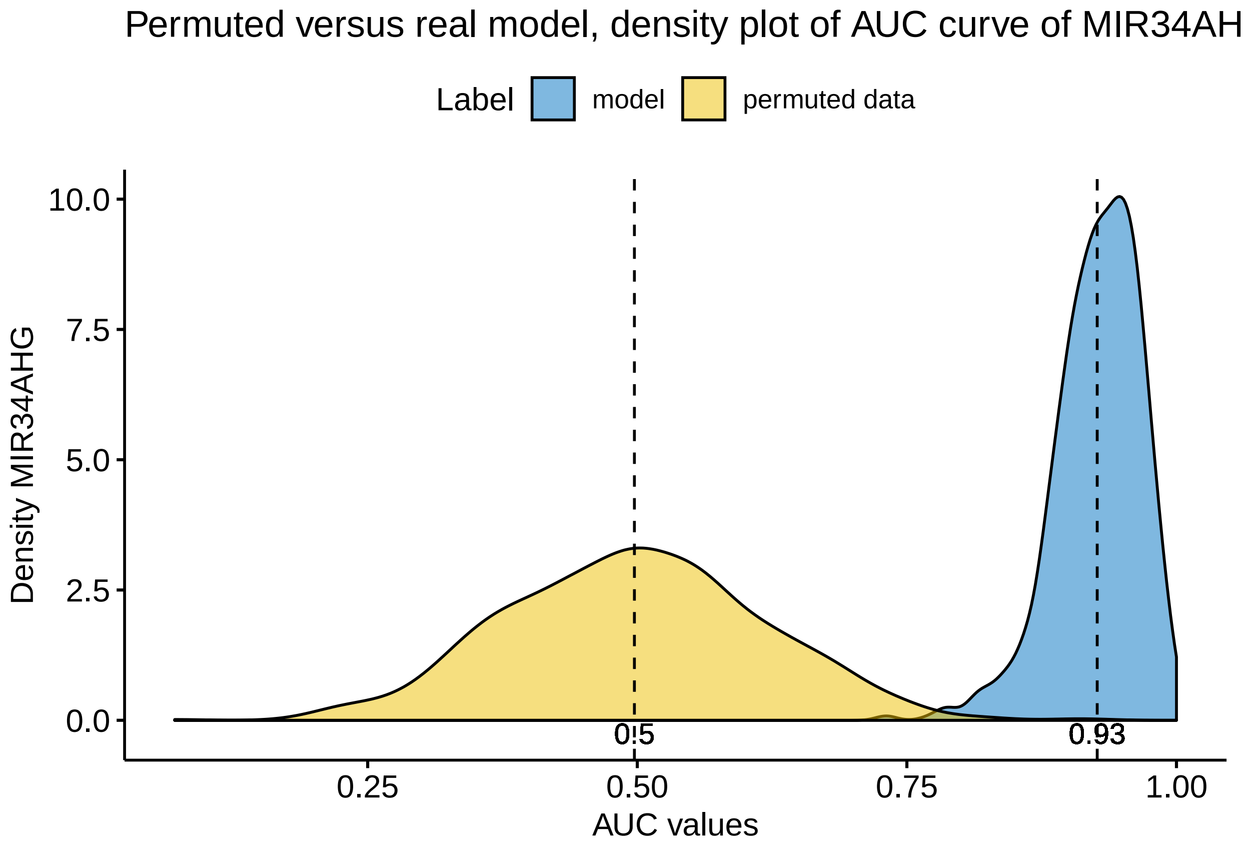
Permuted versus real model, density plot of AUC curve of MIR34AH
Label
model
permuted data
10.0
7.5
5.0
2.5
0.0
Density MIR34AHG
0.5
0.93
0.25
0.50
0.75
1.00
AUC values

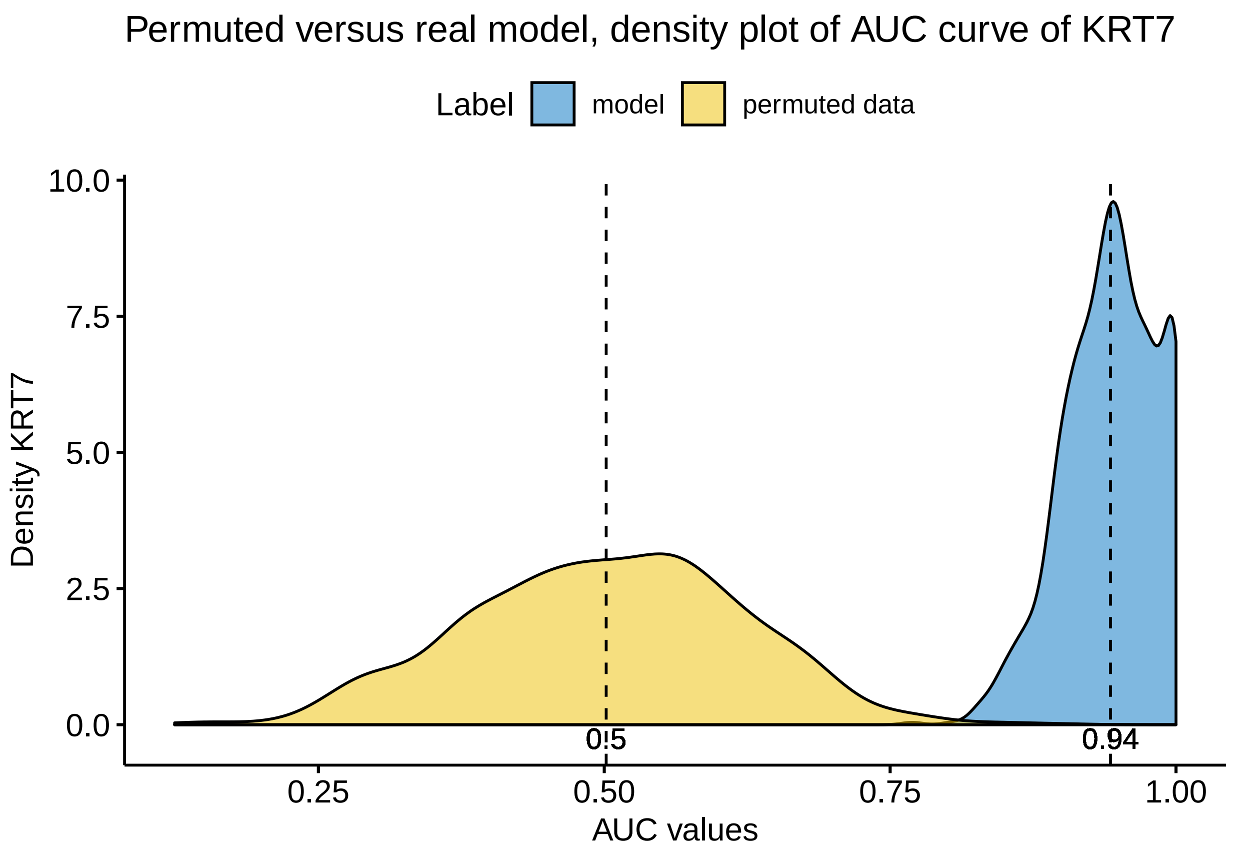


```
n <- length(plot)

# ###Accuracy, Precision etc
#
plot3<-list()
plot4<-list()
Data<-c("Accuracy","Sensitivity","Specificity","Precision")
##### End Block 6
```

```
##### Begin Block 7


#
#Accuracy
Mono<-NULL
MonoR<-NULL
for (s in 1:(NumVar-1)){
  for (d in 1:4){
    for (j in 1:N){
      Mono1<-data.frame(Mono=MatsinglePlus[s,j][[1]][[d]])
      Mono<-rbind(Mono,Mono1)
      Mono1R<-data.frame(MonoR=MatsinglePlusR[s,j][[1]][[d]])
      MonoR<-rbind(MonoR,Mono1R)
    }
    MonoLab<-data.frame(Mono=Mono,Label=as.factor(c(rep("model",dim(Mono)[1]))))
    MonoLabR<-data.frame(Mono=MonoR,Label=as.factor(c(rep("permuted data",dim(MonoR)[1]))))
    colnames(MonoLabR)[1]<-c("Mono")
```

```
    Means<-data.frame(Mean=mean(MonoLab$Mono),MeanR=mean(MonoLabR$Mono))
    Mono<-rbind(MonoLab,MonoLabR)
    plot3[[d]]<-ggdensity(Mono, x = "Mono", fill = "Label", palette = "jco")+labs(title= paste("Value :
    Mono<-NULL
    MonoR<-NULL

  }
  print(grid.arrange(plot3[[2]], plot3[[3]],plot3[[4]],plot3[[1]], ncol=2))

}
```

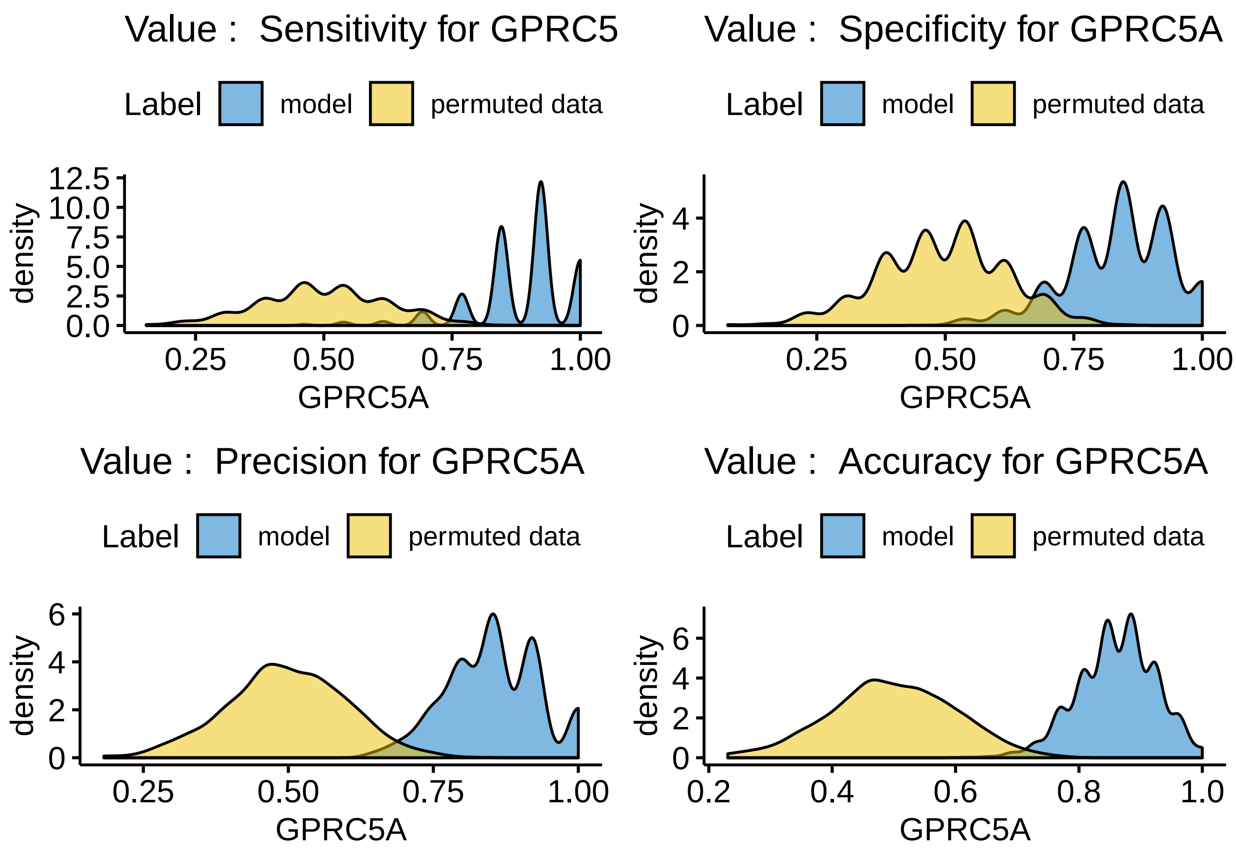


```
## TableGrob (2 x 2) "arrange": 4 grobs
##   z     cells    name           grob
## 1 1 (1-1,1-1) arrange gtable[layout]
## 2 2 (1-1,2-2) arrange gtable[layout]
## 3 3 (2-2,1-1) arrange gtable[layout]
## 4 4 (2-2,2-2) arrange gtable[layout]
```

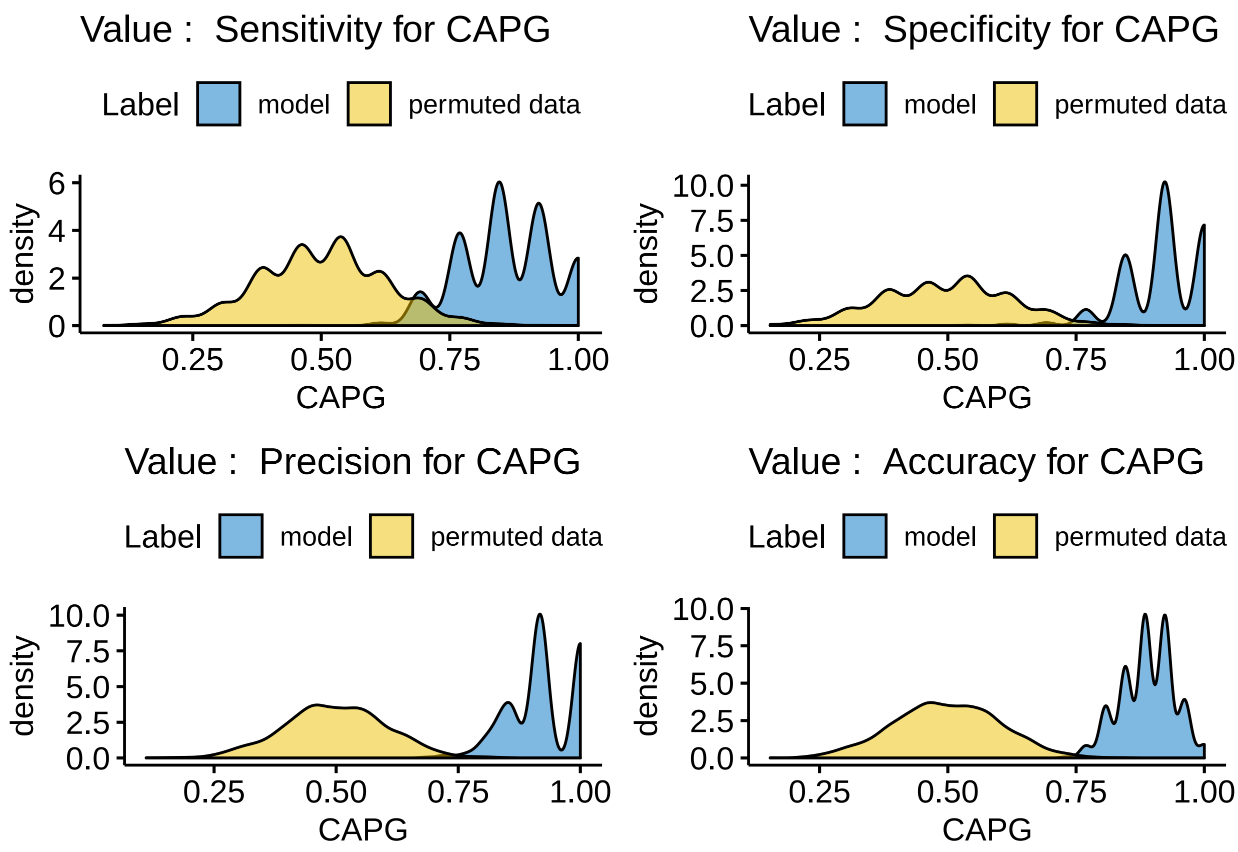


```
## TableGrob (2 x 2) "arrange": 4 grobs
##   z     cells    name           grob
## 1 1 (1-1,1-1) arrange gtable[layout]
## 2 2 (1-1,2-2) arrange gtable[layout]
## 3 3 (2-2,1-1) arrange gtable[layout]
## 4 4 (2-2,2-2) arrange gtable[layout]
```

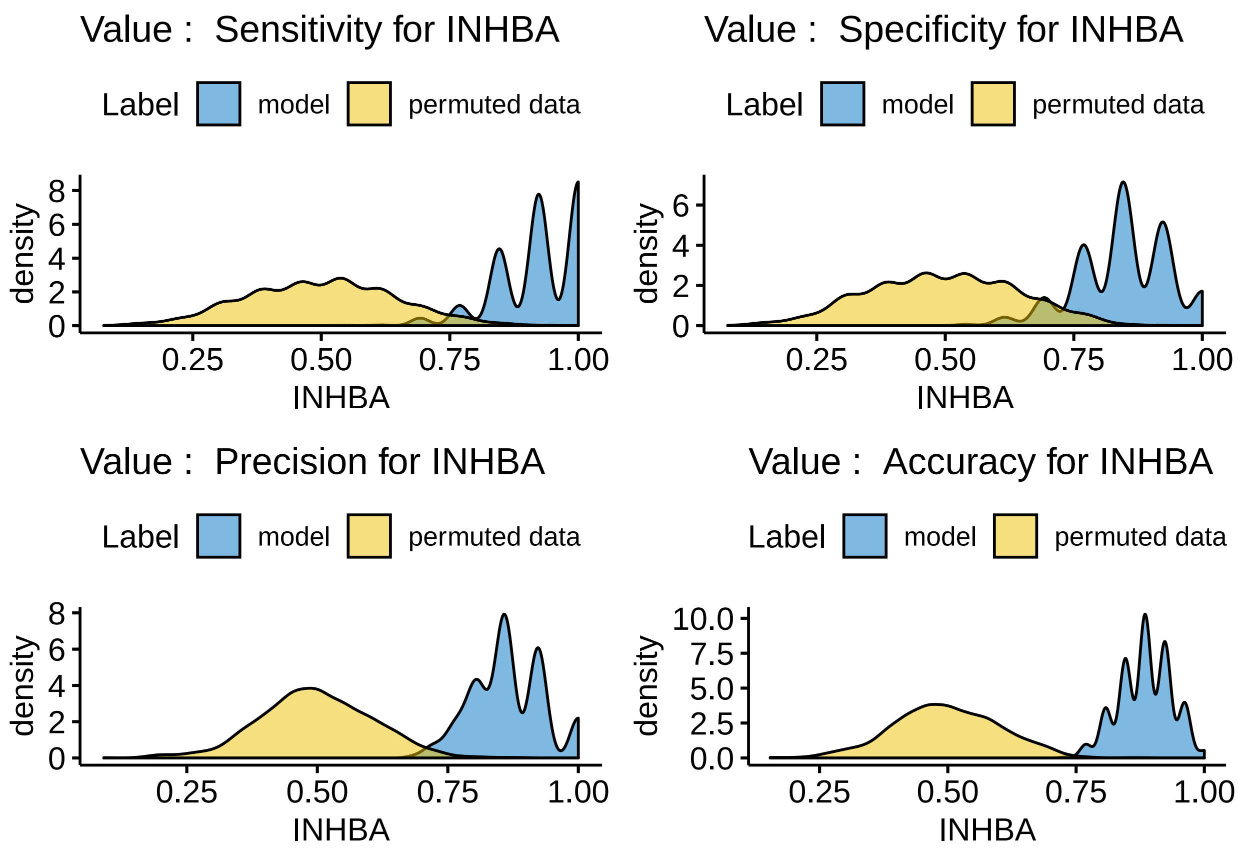


```
## TableGrob (2 x 2) "arrange": 4 grobs
##   z     cells    name           grob
## 1 1 (1-1,1-1) arrange gtable[layout]
## 2 2 (1-1,2-2) arrange gtable[layout]
## 3 3 (2-2,1-1) arrange gtable[layout]
## 4 4 (2-2,2-2) arrange gtable[layout]
```

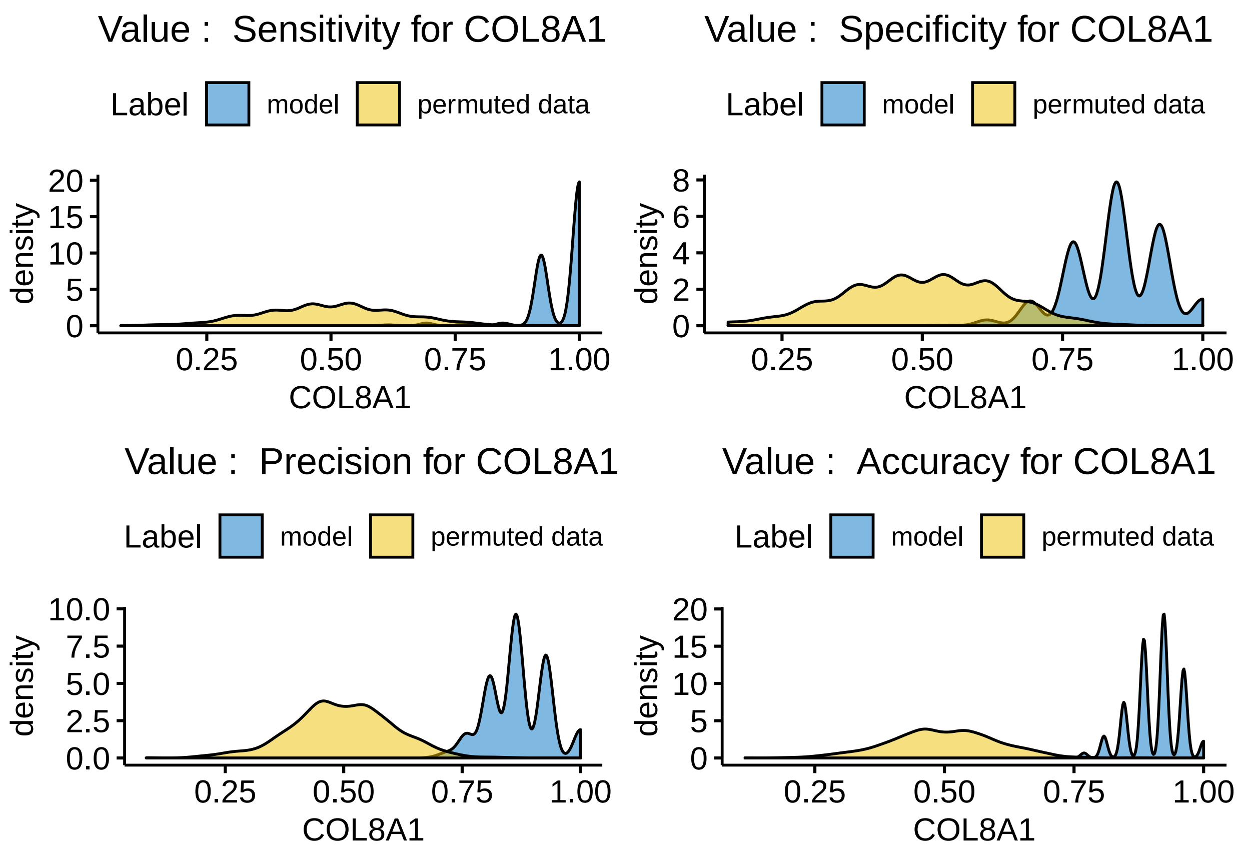


```
## TableGrob (2 x 2) "arrange": 4 grobs
##   z     cells    name           grob
## 1 1 (1-1,1-1) arrange gtable[layout]
## 2 2 (1-1,2-2) arrange gtable[layout]
## 3 3 (2-2,1-1) arrange gtable[layout]
## 4 4 (2-2,2-2) arrange gtable[layout]
```

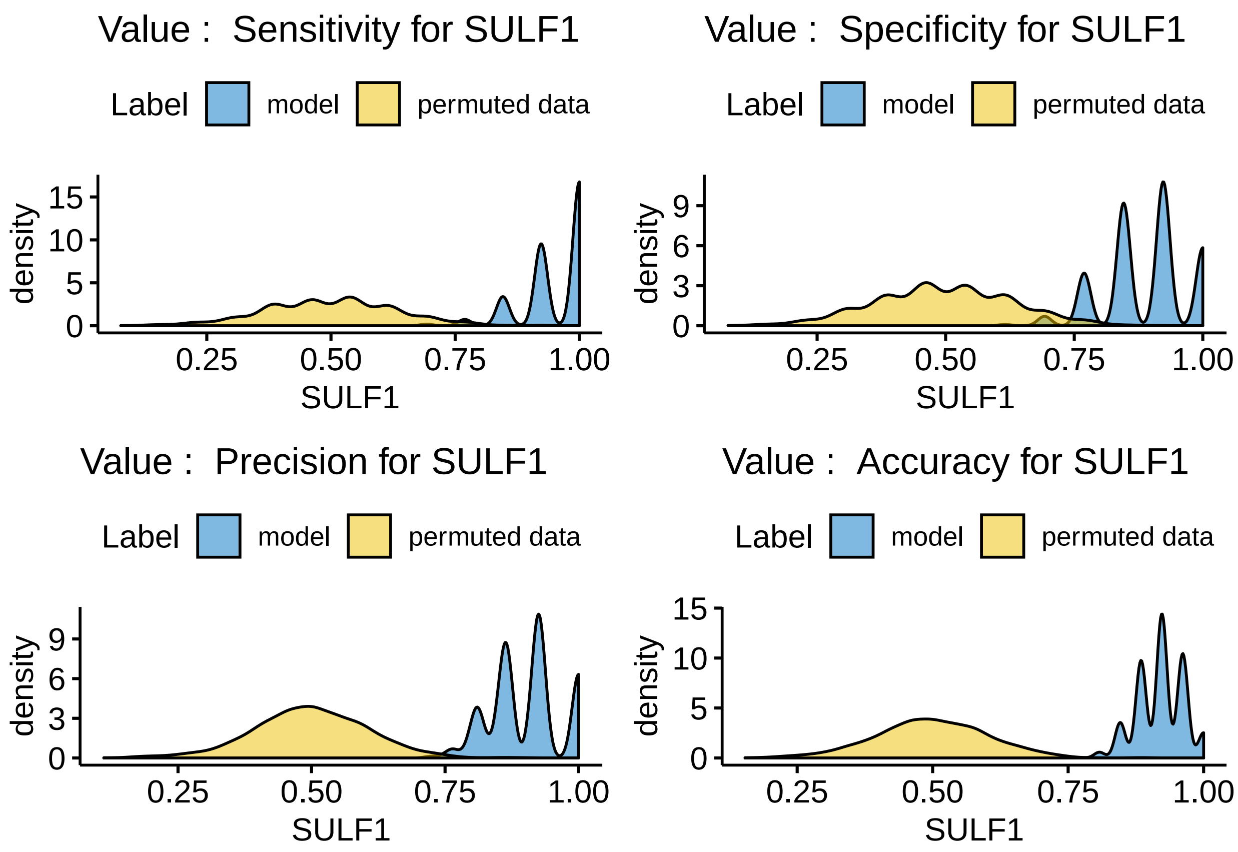


```
## TableGrob (2 x 2) "arrange": 4 grobs
##   z     cells    name           grob
## 1 1 (1-1,1-1) arrange gtable[layout]
## 2 2 (1-1,2-2) arrange gtable[layout]
## 3 3 (2-2,1-1) arrange gtable[layout]
## 4 4 (2-2,2-2) arrange gtable[layout]
```

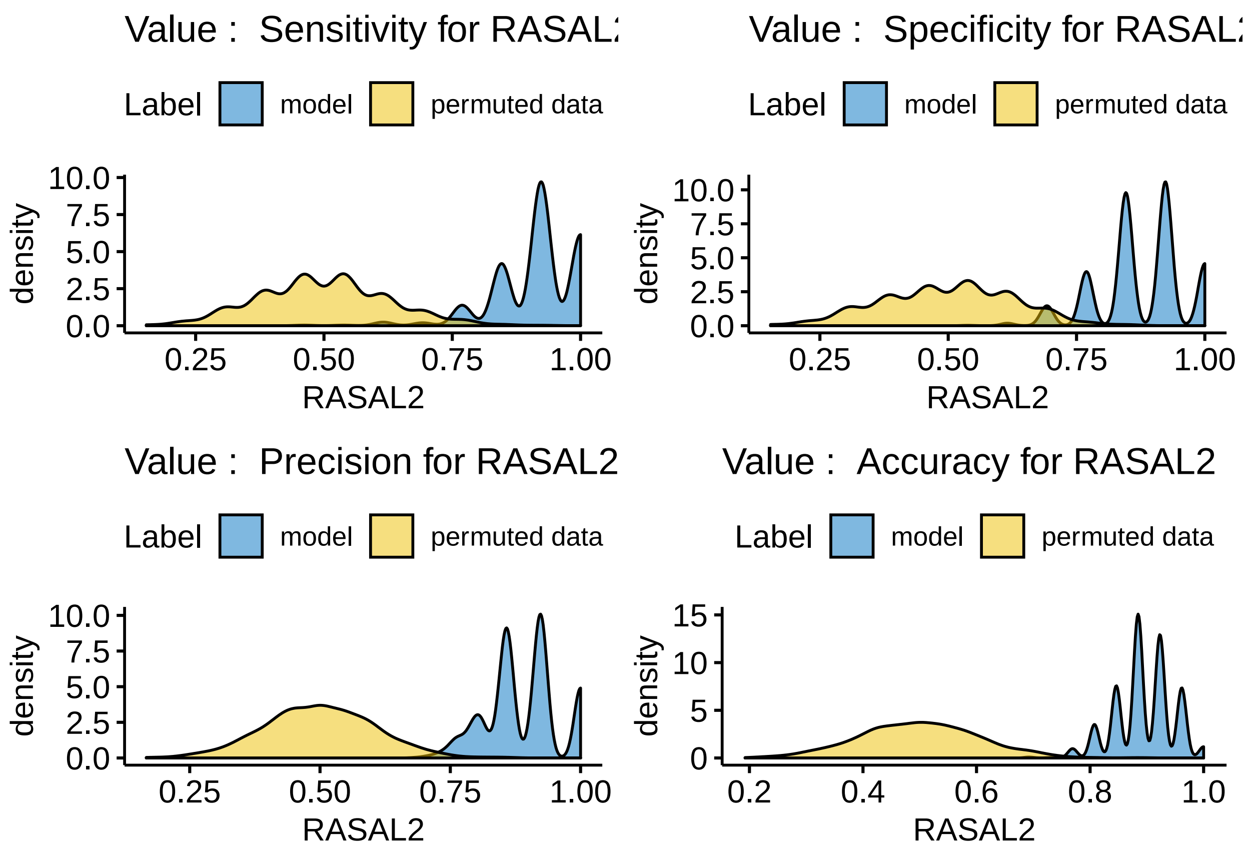


```
## TableGrob (2 x 2) "arrange": 4 grobs
##   z     cells    name           grob
## 1 1 (1-1,1-1) arrange gtable[layout]
## 2 2 (1-1,2-2) arrange gtable[layout]
## 3 3 (2-2,1-1) arrange gtable[layout]
## 4 4 (2-2,2-2) arrange gtable[layout]
```

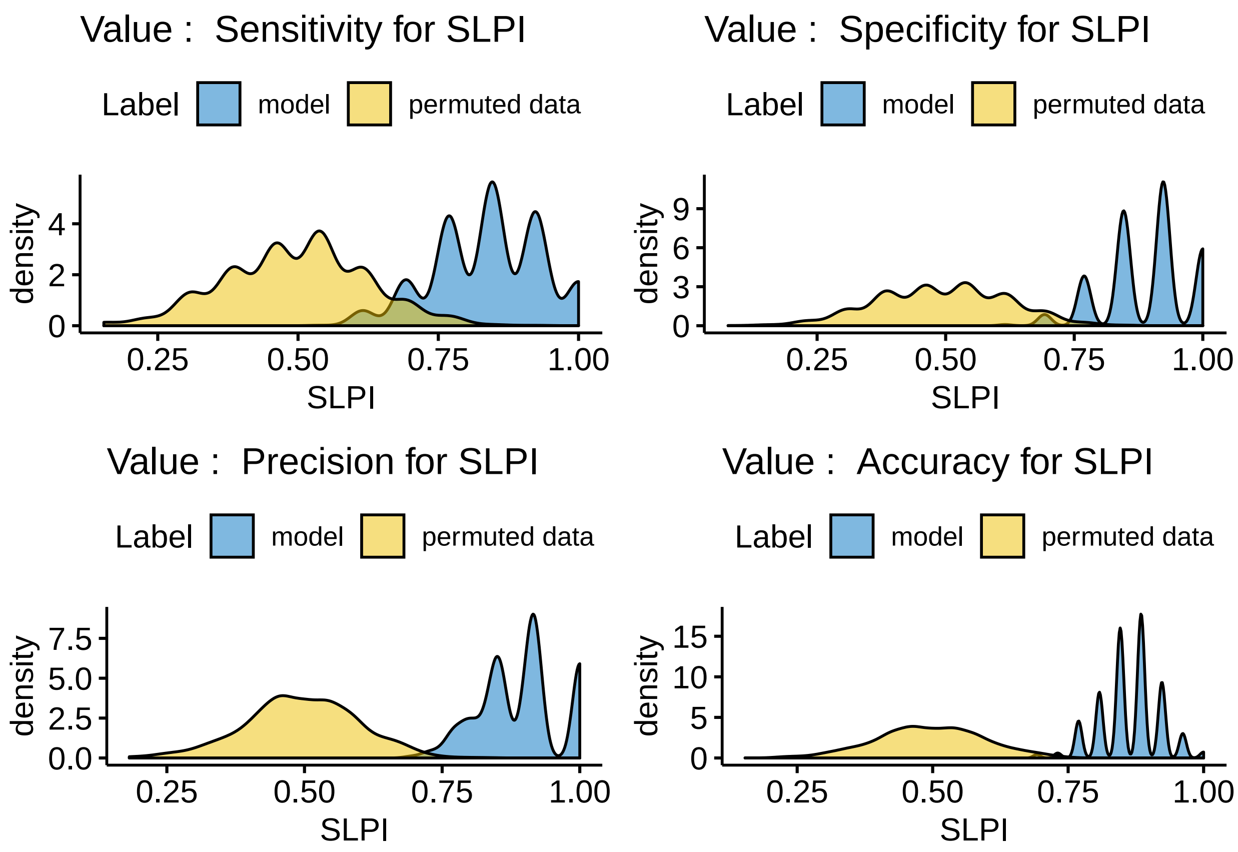


```
## TableGrob (2 x 2) "arrange": 4 grobs
##   z     cells    name           grob
## 1 1 (1-1,1-1) arrange gtable[layout]
## 2 2 (1-1,2-2) arrange gtable[layout]
## 3 3 (2-2,1-1) arrange gtable[layout]
## 4 4 (2-2,2-2) arrange gtable[layout]
```

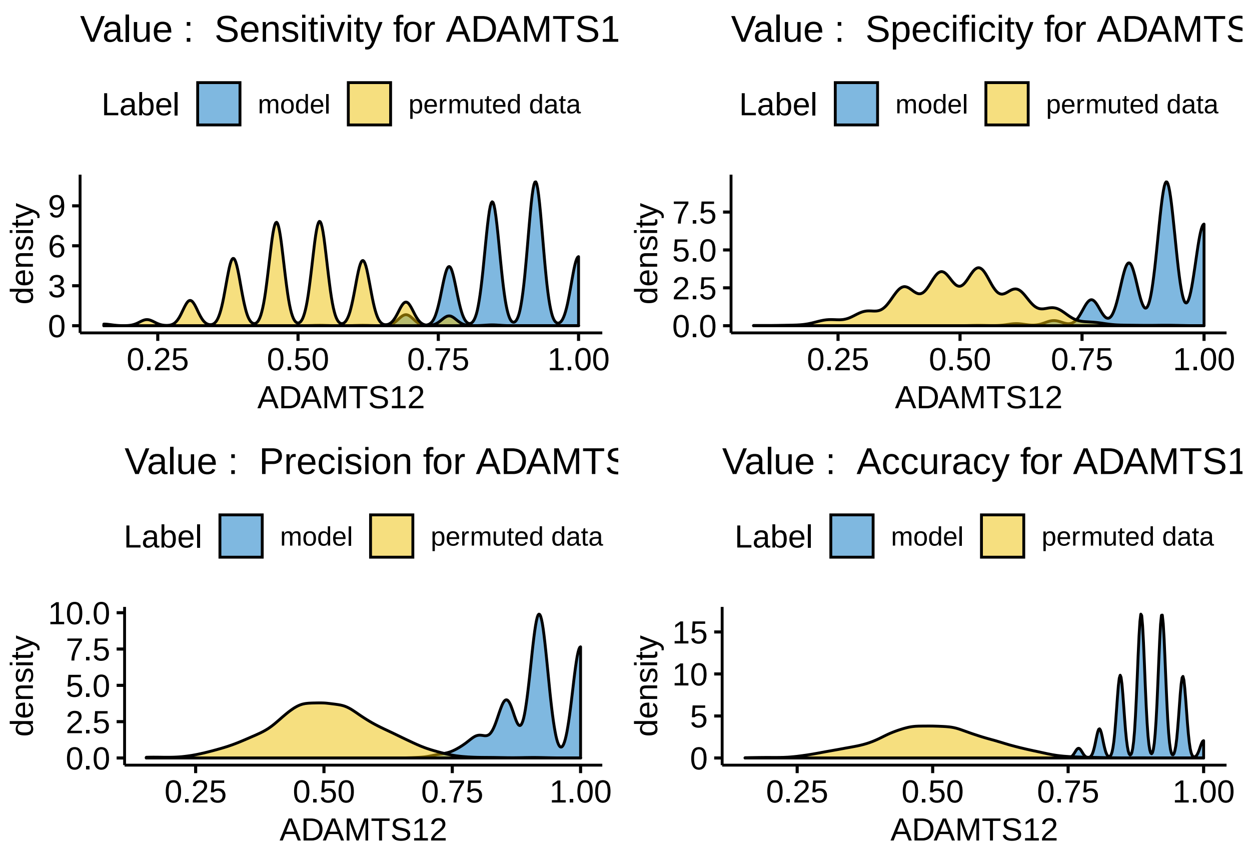


```
## TableGrob (2 x 2) "arrange": 4 grobs
##   z     cells    name           grob
## 1 1 (1-1,1-1) arrange gtable[layout]
## 2 2 (1-1,2-2) arrange gtable[layout]
## 3 3 (2-2,1-1) arrange gtable[layout]
## 4 4 (2-2,2-2) arrange gtable[layout]
```

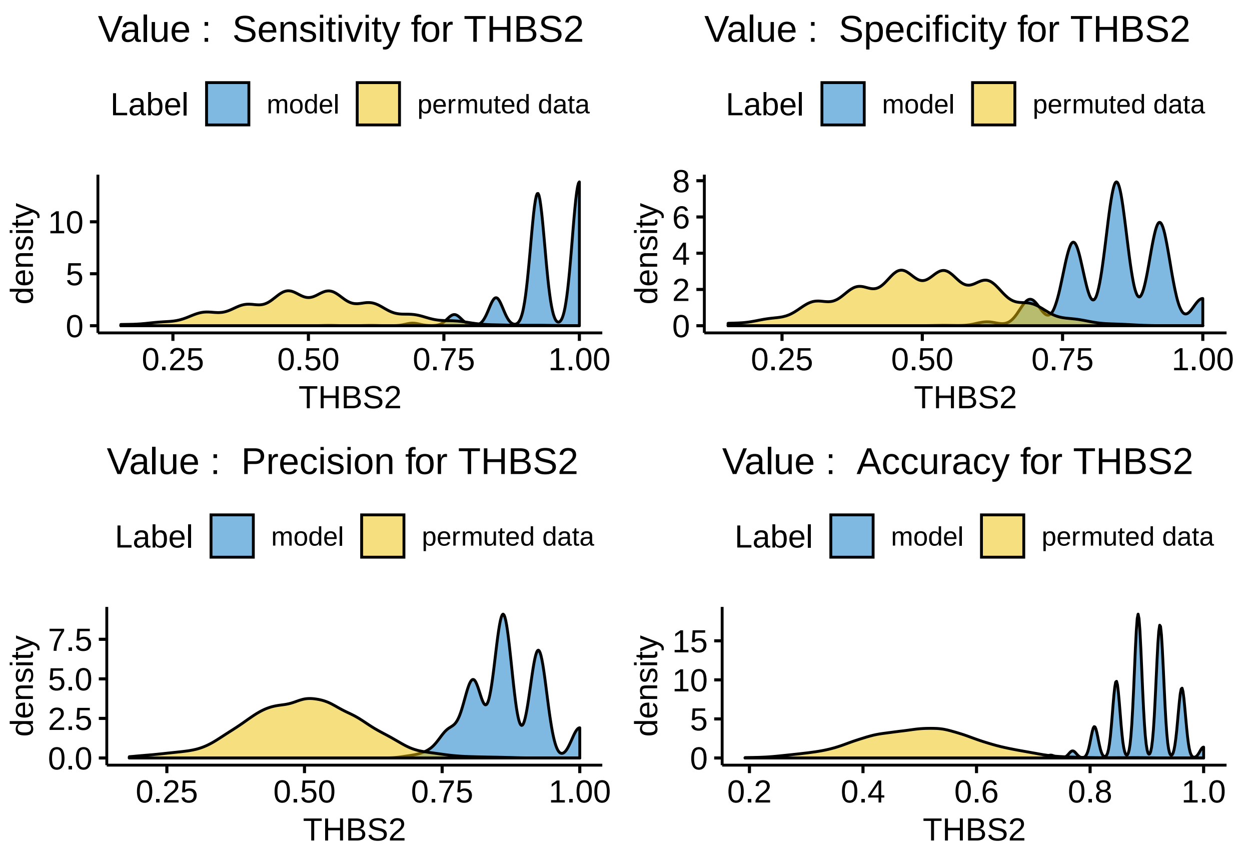


```
## TableGrob (2 x 2) "arrange": 4 grobs
##   z     cells    name           grob
## 1 1 (1-1,1-1) arrange gtable[layout]
## 2 2 (1-1,2-2) arrange gtable[layout]
## 3 3 (2-2,1-1) arrange gtable[layout]
## 4 4 (2-2,2-2) arrange gtable[layout]
```

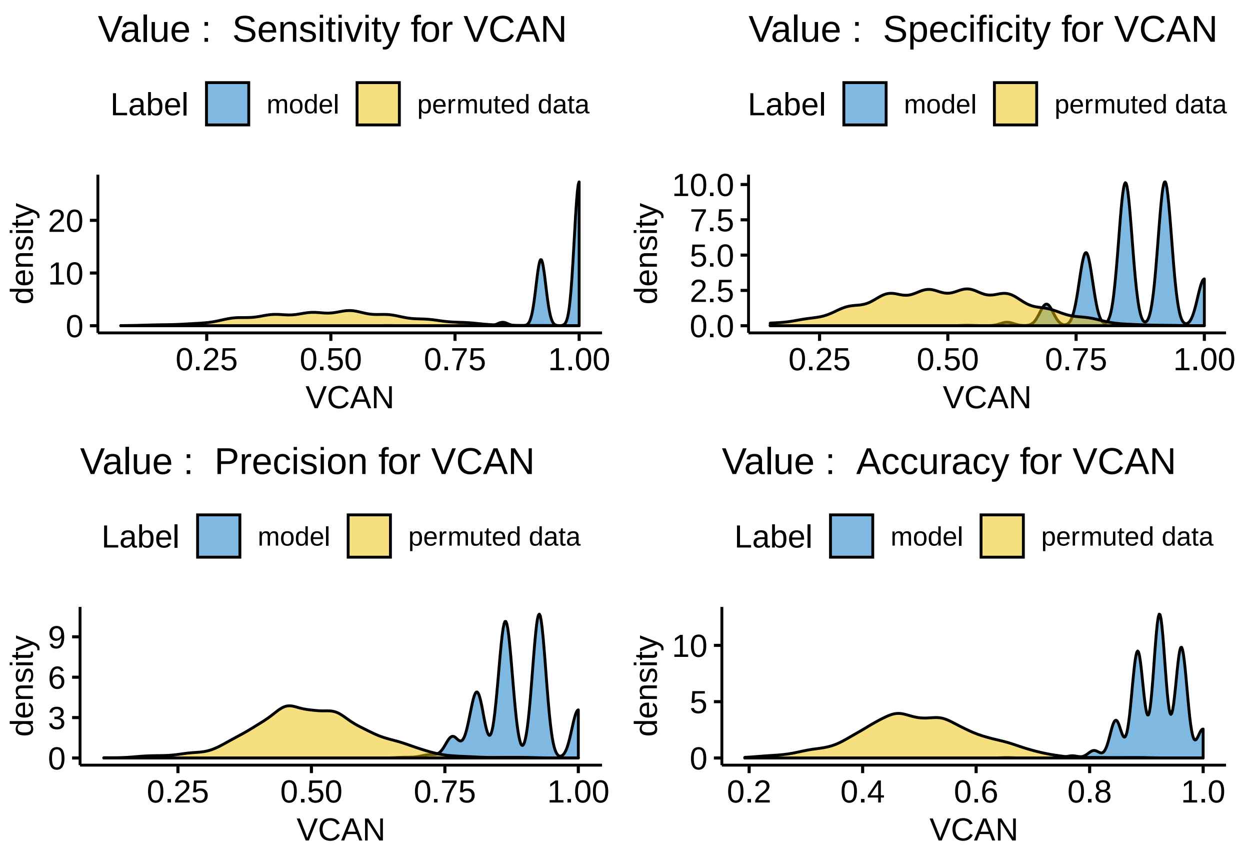


```
## TableGrob (2 x 2) "arrange": 4 grobs
##   z     cells    name           grob
## 1 1 (1-1,1-1) arrange gtable[layout]
## 2 2 (1-1,2-2) arrange gtable[layout]
## 3 3 (2-2,1-1) arrange gtable[layout]
## 4 4 (2-2,2-2) arrange gtable[layout]
```

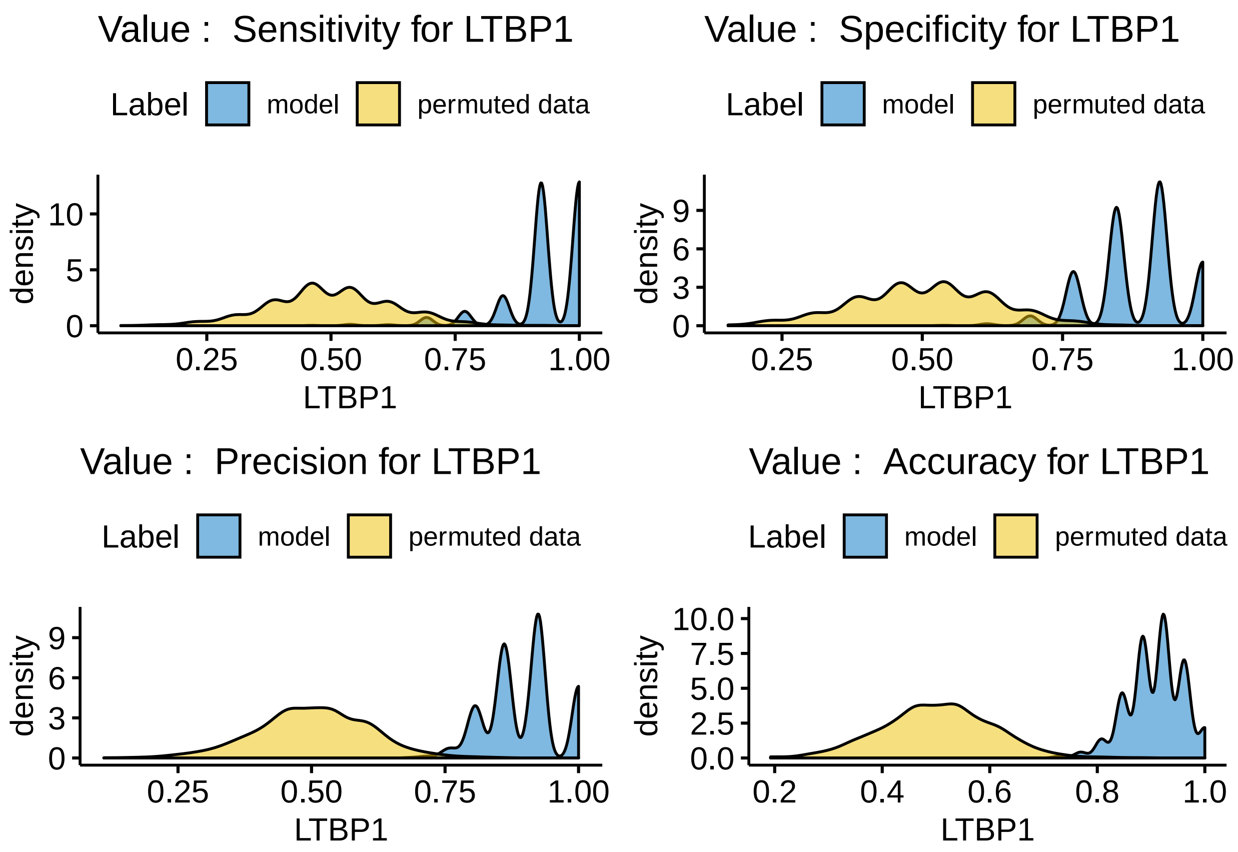


```
## TableGrob (2 x 2) "arrange": 4 grobs
##   z     cells    name           grob
## 1 1 (1-1,1-1) arrange gtable[layout]
## 2 2 (1-1,2-2) arrange gtable[layout]
## 3 3 (2-2,1-1) arrange gtable[layout]
## 4 4 (2-2,2-2) arrange gtable[layout]
```

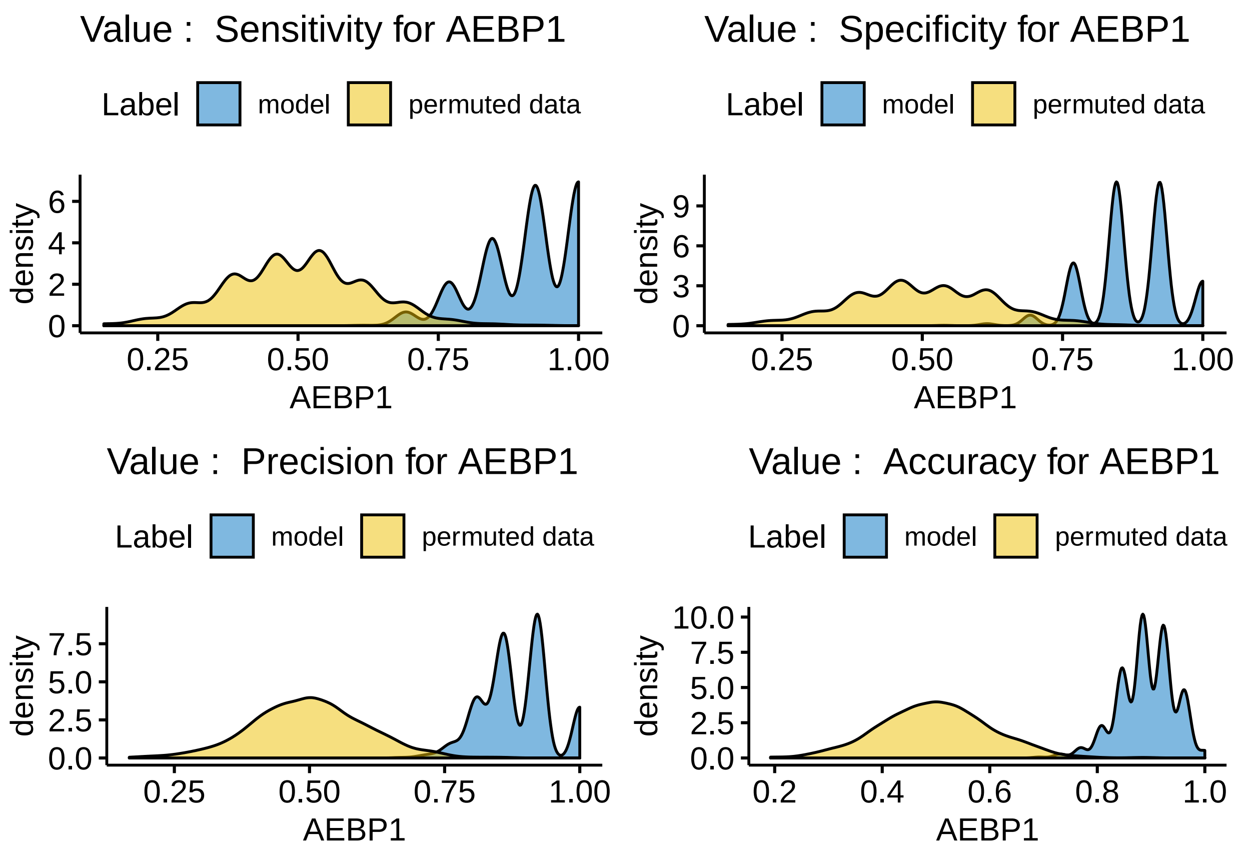


```
## TableGrob (2 x 2) "arrange": 4 grobs
##   z     cells    name           grob
## 1 1 (1-1,1-1) arrange gtable[layout]
## 2 2 (1-1,2-2) arrange gtable[layout]
## 3 3 (2-2,1-1) arrange gtable[layout]
## 4 4 (2-2,2-2) arrange gtable[layout]
```

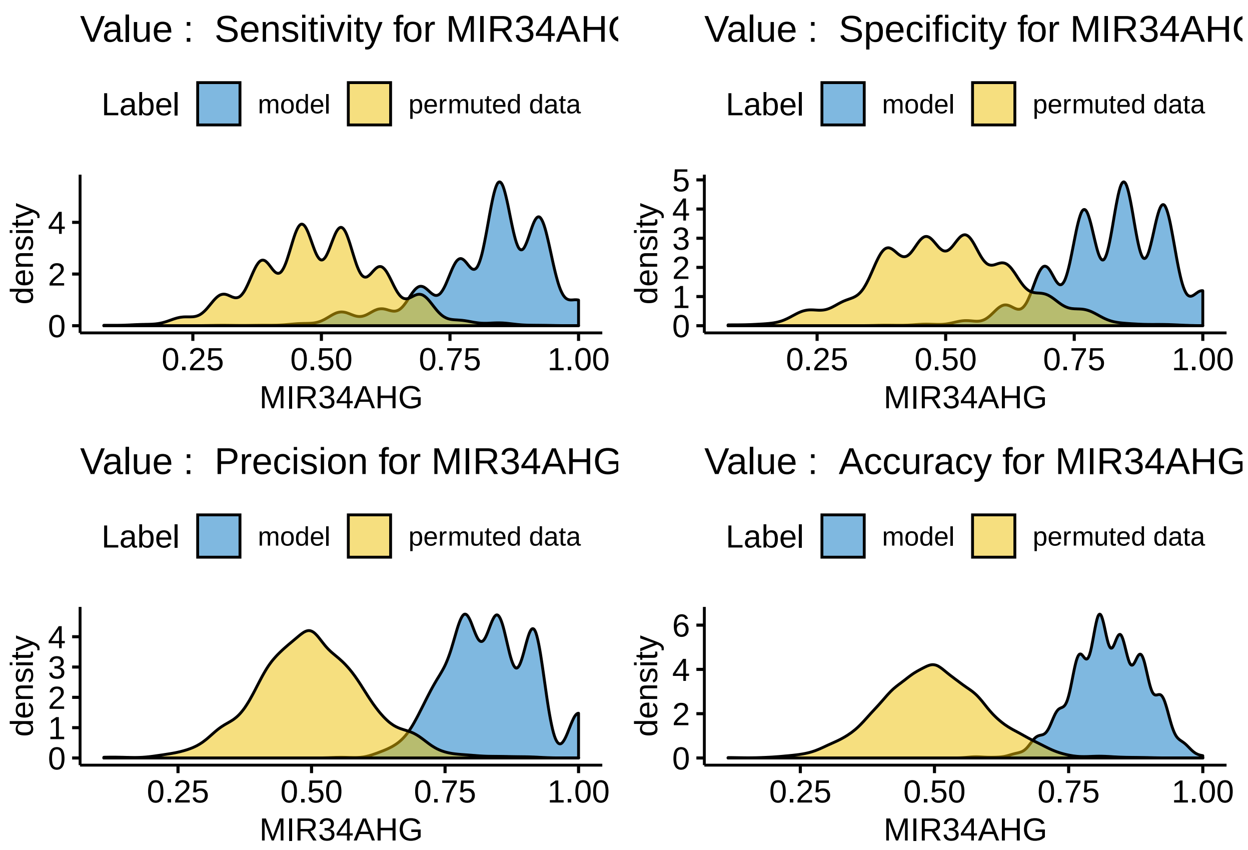


```
## TableGrob (2 x 2) "arrange": 4 grobs
##   z     cells    name           grob
## 1 1 (1-1,1-1) arrange gtable[layout]
## 2 2 (1-1,2-2) arrange gtable[layout]
## 3 3 (2-2,1-1) arrange gtable[layout]
## 4 4 (2-2,2-2) arrange gtable[layout]
```

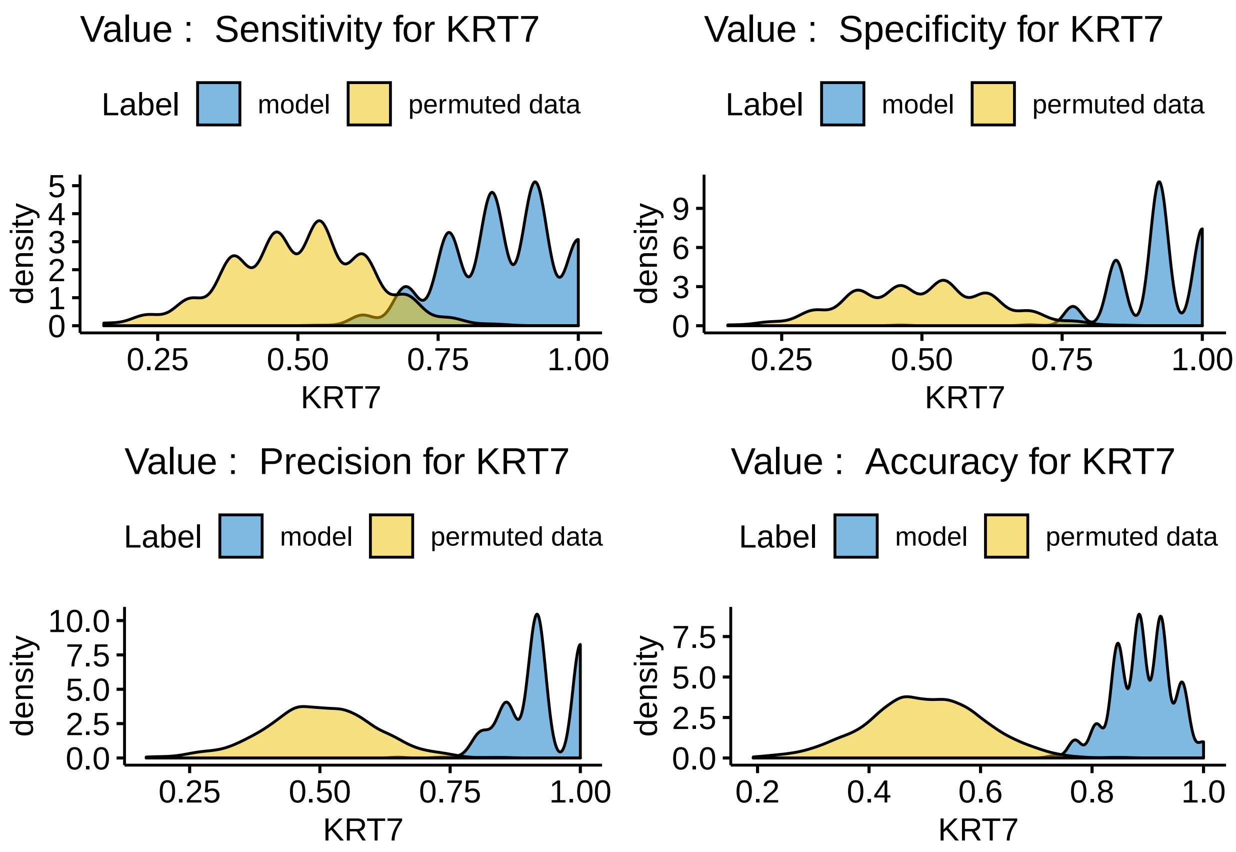


```
## TableGrob (2 x 2) "arrange": 4 grobs
##   z     cells    name           grob
## 1 1 (1-1,1-1) arrange gtable[layout]
## 2 2 (1-1,2-2) arrange gtable[layout]
## 3 3 (2-2,1-1) arrange gtable[layout]
## 4 4 (2-2,2-2) arrange gtable[layout]
```

```
Meanq<-data.frame(multipleAUC$Means,multipleAUCR$Means)
MA<-data.frame(Mono=t(multipleAUC[1:dim(multipleAUC)[2]-1]))
MA["Label"]<-as.factor(c(rep("model",dim(MA)[1])))
MAR<-data.frame(Mono=t(multipleAUCR[1:dim(multipleAUCR)[2]-1]))
MAR["Label"]<-as.factor(c(rep("permuted data",dim(MAR)[1])))
Mono<-rbind(MA,MAR)
pp<-ggdensity(Mono, x = "Mono", fill = "Label", palette = "jco")+geom_vline(xintercept=Meanq[1,1],linet
print(pp+geom_vline(xintercept=Meanq[1,2],linetype = 2,color="black",show.legend = TRUE))
```

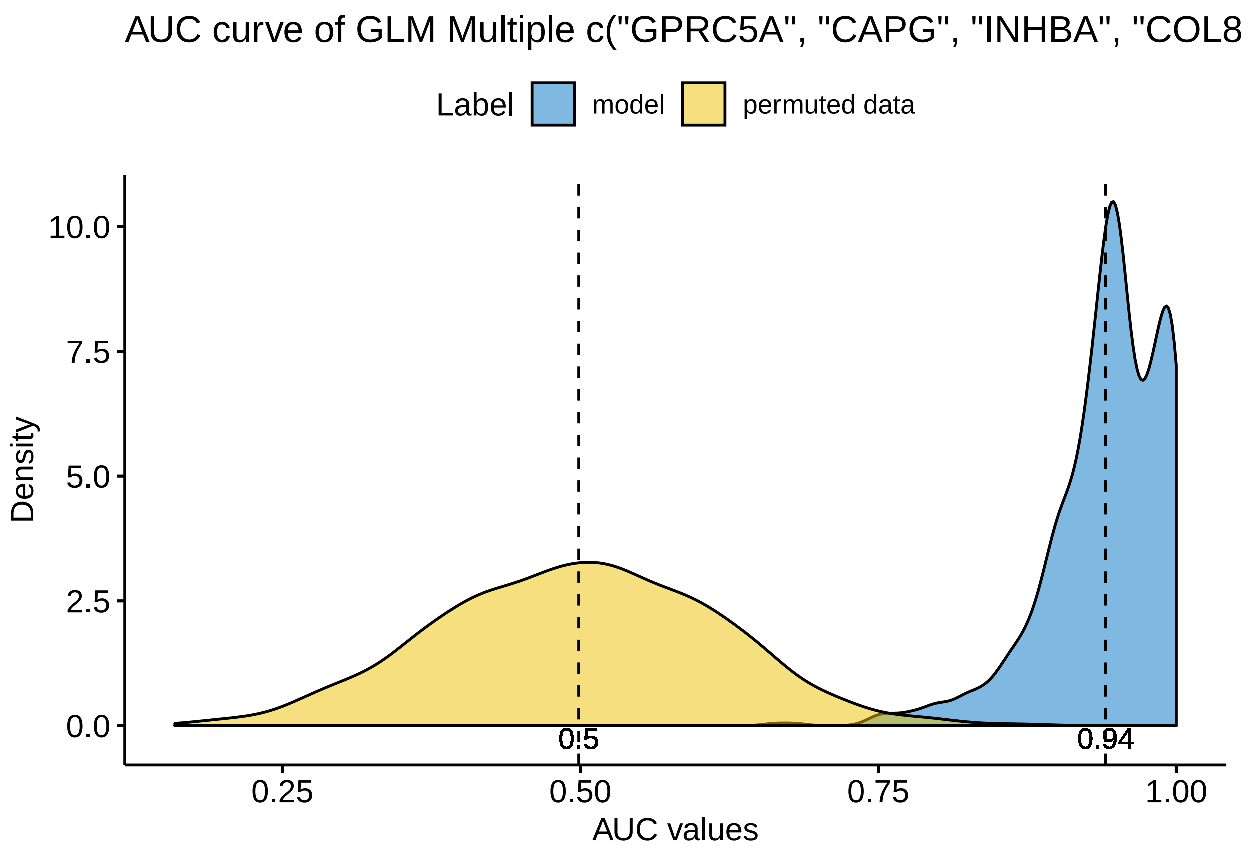


```
Meanq<-data.frame(multipleAUCNB$Means,multipleAUCNBR$Means)
MA<-data.frame(Mono=t(multipleAUCNB[1:dim(multipleAUCNB)[2]-1]))
MA["Label"]<-as.factor(c(rep("model",dim(MA)[1])))
MAR<-data.frame(Mono=t(multipleAUCNBR[1:dim(multipleAUCNBR)[2]-1]))
MAR["Label"]<-as.factor(c(rep("permuted data",dim(MAR)[1])))
Mono<-rbind(MA,MAR)
pp<-ggdensity(Mono, x = "Mono", fill = "Label", palette = "jco")+geom_vline(xintercept=Meanq[1,1],linet
print(pp+geom_vline(xintercept=Meanq[1,2],linetype = 2,color="black",show.legend = TRUE))
```

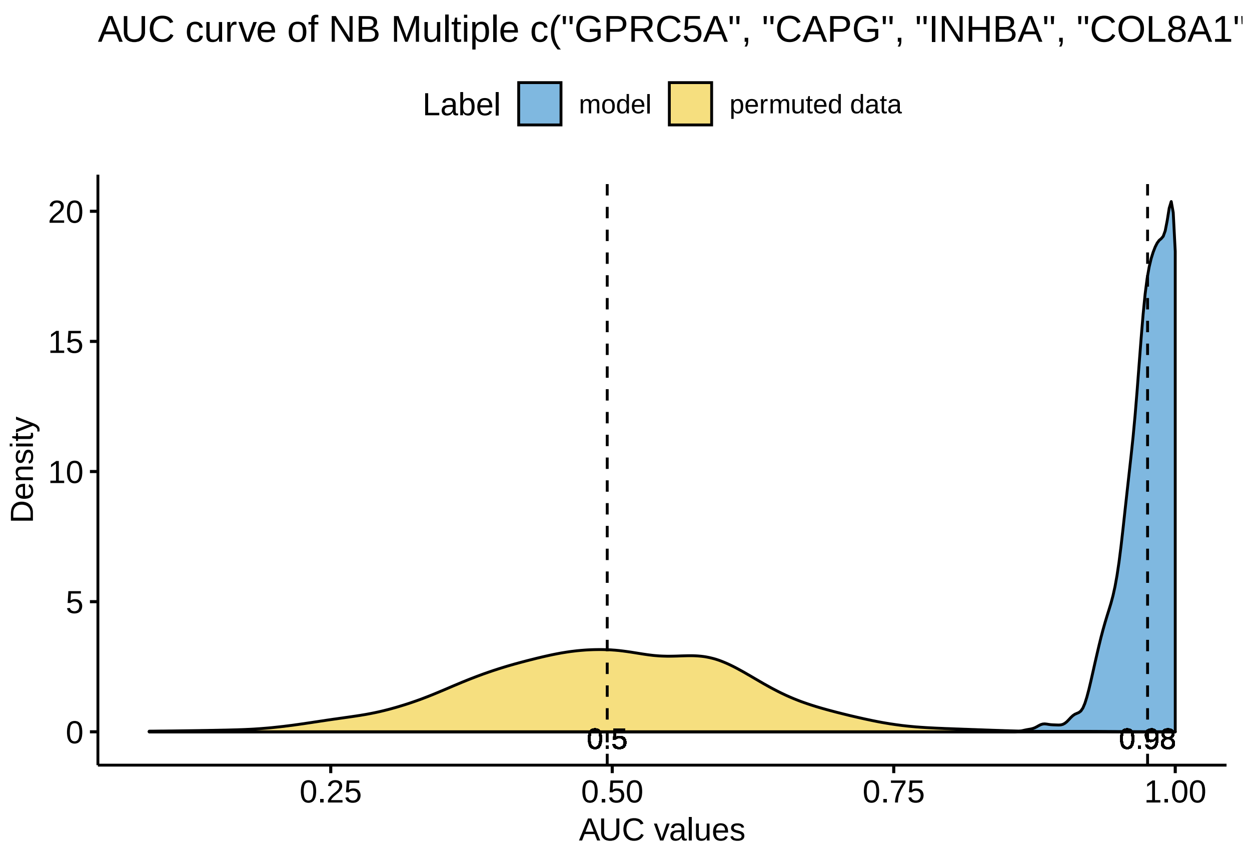


```
ee<-regex(names,".")

Final<-data.frame(MultiNB=t(multipleAUCNB),MultiNBRand=t(multipleAUCNBR),Multi=t(multipleAUC),MultiRand=
FinalMeans<-data.frame(MultiNB=multipleAUCNB$Means,MultiNBRand=multipleAUCNBR$Means,Multi=multipleAUC$Me

##### End Block 7


genes_to_network <- colnames(Betas_select)[-length(colnames(Betas_select))]
genes_to_networkStrict <- colnames(Betas_select2)[-length(colnames(Betas_select2))]


sink("genes_to_network.txt")
cat("Beta_Select_Intial")
```

```
## Beta_Select_Intial
```

```
cat("\n")
```

```
cat(genes_to_network, sep = "\n")
```

```
## SULF1
## INHBA
## COL10A1
## COL8A1
## FN1
## NTM
```

```
## NOX4
## THBS2
## ADAMTS12
## RASAL2
## CAPG
## FAP
## CTHRC1
## VCAN
## WISP1
## TIMP1
## LTBP1
## SLPI
## GPRC5A
## MIR34AHG
## AEBP1
## KRT7
```

```r
cat("Beta_Select_Threshold")
```

```
## Beta_Select_Threshold
```

```r
cat("\n")
```

```r
cat(genes_to_networkStrict, sep = "\n")
```

```
## GPRC5A
## CAPG
## INHBA
## COL8A1
## SULF1
## RASAL2
## SLPI
## ADAMTS12
## THBS2
## VCAN
## LTBP1
## AEBP1
## MIR34AHG
## KRT7
```

```r
sink()
```
