# Supplementary material for: -Omics biomarker identification pipeline for translational medicine
Source: J Transl Med. 2019 May 14;17:155. doi: 10.1186/s12967-019-1912-5 (PMC6518609; doi:10.1186/s12967-019-1912-5)
Supplement: Supplementary file 2 — Additional file 2. Lipids identified in three cohorts are listed with different category. Category A: HM vs. Mixed (FM and HM combined) feeding; Category B: FM vs. Mixed (FM and HM combined ) feeding; Category C: HM vs. FM. [file 12967_2019_1912_MOESM2_ESM.pptx]

## Slide 1
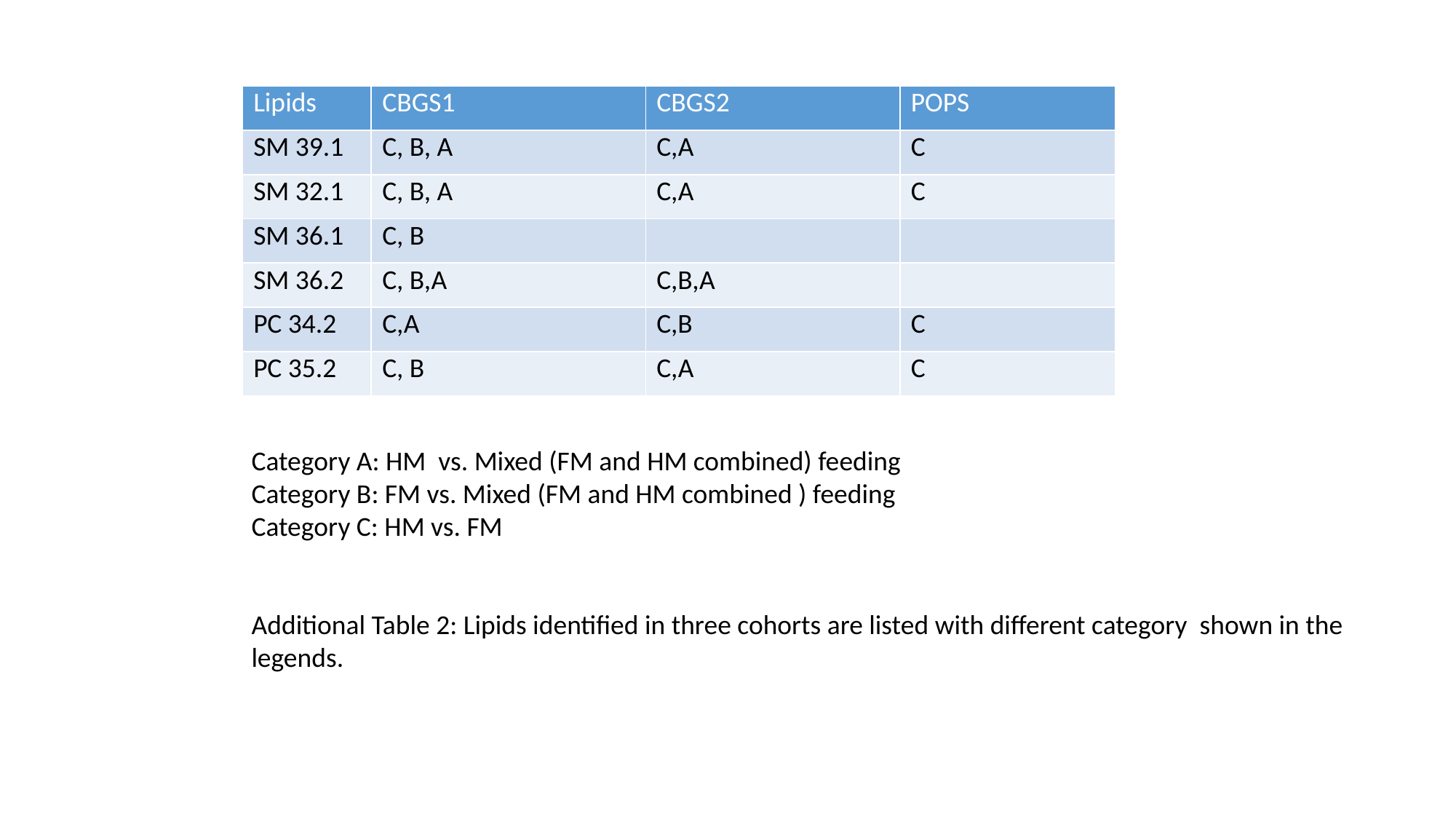

| Lipids | CBGS1 | CBGS2 | POPS |
| --- | --- | --- | --- |
| SM 39.1 | C, B, A | C,A | C |
| SM 32.1 | C, B, A | C,A | C |
| SM 36.1 | C, B | | |
| SM 36.2 | C, B,A | C,B,A | |
| PC 34.2 | C,A | C,B | C |
| PC 35.2 | C, B | C,A | C |
Category A: HM vs. Mixed (FM and HM combined) feeding
Category B: FM vs. Mixed (FM and HM combined ) feeding
Category C: HM vs. FM
Additional Table 2: Lipids identified in three cohorts are listed with different category shown in the legends.
