# Supplementary material for: -Omics biomarker identification pipeline for translational medicine
Source: J Transl Med. 2019 May 14;17:155. doi: 10.1186/s12967-019-1912-5 (PMC6518609; doi:10.1186/s12967-019-1912-5)
Supplement: Supplementary file 3 — Additional file 3. R Markdown analysis results from the workflow developed on GSE15061. [file 12967_2019_1912_MOESM3_ESM.pdf]

AML Cancer Analysis

Bravo, Williams, and Acharjee

19 Sept 2018

```
#Libraries



##### Begin Block 1

setwd("~/AnimeshReview/LauraPaper/Journal of Translational Medicine /LauraFinal/")
library(readxl)
library(data.table)
library(tidyverse)
library(dplyr)
library(devtools)
library(ggcorrplot)
library(car)
library(ggpubr)
library(glmnet)
library(summarytools)
library(knitr)
library(htmltools)
library(corrplot)
library(caret)
library(factoextra)
library(Metrics)
library(readr)
library(gplots)
library(dplyr)
library(stringr)
library(readxl)
library(plotly)
library(e1071)
library(ggplot2)
library(reshape2)
library(multtest)
library(ROCR)
library(gridExtra)
library(MLmetrics)


#### Begin Load Laura's Functions
doubleAUCfun <- function(xtrain, ytrain,xtest,ytest,s,k) {

    yy3<-data.frame(Outcome=ytrain, Predictor=xtrain[,s],Predictor2=xtrain[,k])
    model3<-glm(Outcome~Predictor+Predictor2,data=yy3, family=binomial(link='logit'))
    new3<-data.frame(Predictor=xtest[,s],Predictor2=xtest[,k])
    p3<-predict(model3,new3,type="response")
    new5<-data.frame(Outcome=ytest)
    pr3 <- prediction(p3, new5)
```

```r
    prf3 <- performance(pr3, measure = "tpr", x.measure = "fpr")
    auc3 <- performance(pr3, measure = "auc")
    auc3 <- auc3@y.values[[1]]
    result2<-auc3
}

doubleAUCfunNB <- function(xtrain, ytrain,xtest,ytest,s,k) {

    yy3<-data.frame(Outcome=ytrain, Predictor=xtrain[,s],Predictor2=xtrain[,k])
    model4<-naiveBayes(Outcome~Predictor+Predictor2,data=yy3)
    new4<-data.frame(Predictor=xtest[,s],Predictor2=xtest[,k])
    p4<-predict(model4,new4,type="raw")
    #without raw write confusion matrix directly
    prob2<-NULL
    for (i in 1:dim(p4)[1]){
        prob2[i]<-p4[i,2]/p4[i,1]
    }
    new5<-data.frame(Outcome=ytest)
    pr3 <- prediction(prob2, new5)
    prf3 <- performance(pr3, measure = "tpr", x.measure = "fpr")
    auc3 <- performance(pr3, measure = "auc")
    auc3 <- auc3@y.values[[1]]
    result2<-auc3
}

doubleAUCfunRFCross <- function(xtrain, ytrain,xtest,ytest,s,k) {

    yy3<-data.frame(Outcome=ytrain, Predictor=xtrain[,s],Predictor2=xtrain[,k])
    control <- trainControl(method="repeatedcv", number=10, repeats=3) #Is this the only thing needed f
    seed <- 7
    metric <- "Accuracy"
    set.seed(seed)
    #mtry <- 2
    tunegrid <- expand.grid(.mtry=c(1:6))
    set.seed(seed)
    rf_default <- train(Outcome~Predictor+Predictor2,data=yy3, method="rf", metric=metric, tuneGrid=tun
    #print(rf_default)
    new4<-data.frame(Predictor=xtest[,s],Predictor2=xtest[,k])
    pred=predict(rf_default, new4,type="prob")
    pred2=predict(rf_default, new4)
    prob2<-NULL
    for (i in 1:dim(pred)[1]){
        prob2[i]<-pred[i,2]/pred[i,1]
    }
    new5<-data.frame(Outcome=ytest)
    pr3 <- prediction(prob2, new5)
    prf3 <- performance(pr3, measure = "tpr", x.measure = "fpr")
    auc3 <- performance(pr3, measure = "auc")
    auc3 <- auc3@y.values[[1]]
    result2<-auc3

}
```

```
doubleAUCfunSVM <- function(xtrain, ytrain,xtest,ytest,s,k) {

    yy3<-data.frame(Outcome=ytrain, Predictor=xtrain[,s],Predictor2=xtrain[,k])
    seed <- 7
    set.seed(seed)
    model3<-svm(Outcome~Predictor+Predictor2,data=yy3)
    new3<-data.frame(Predictor=xtest[,s],Predictor2=xtest[,k])
    p3<-predict(model3,new3,decision.values = TRUE)
    p4<-attr(p3,"decision.values")
    new5<-data.frame(Outcome=ytest)
    pr3<- prediction(p4, new5)
    #table(p3, new5)
    #confusionMatrix(p3, new5)
    prf3 <- performance(pr3, measure = "tpr", x.measure = "fpr")
    auc3 <- performance(pr3, measure = "auc")
    auc3 <- auc3@y.values[[1]]
    result2<-auc3
}

doubleAUCfunSVMCross <- function(xtrain, ytrain,xtest,ytest,s,k) {

    yy3<-data.frame(Outcome=ytrain, Predictor=xtrain[,s],Predictor2=xtrain[,k])
    seed <- 7
    set.seed(seed)
    model4<-svm(Outcome~Predictor+Predictor2,data=yy3,method="C-classification",
                kernel="radial", gamma = 0.01, cost = 100,cross=10, probability=TRUE)
    new4<-data.frame(Predictor=xtest[,s],Predictor2=xtest[,k])
    p4<-predict(model4,new4,decision.values = TRUE)
    p5<-attr(p4,"decision.values")
    new5<-data.frame(Outcome=ytest)
    pr3 <- prediction(p5, new5)
    prf3 <- performance(pr3, measure = "tpr", x.measure = "fpr")
    auc3 <- performance(pr3, measure = "auc")
    auc3 <- auc3@y.values[[1]]
    result2<-auc3
}

doublePlusfun <- function(xtrain, ytrain,xtest,ytest,s,k) {

    yy3<-data.frame(Outcome=ytrain, Predictor=xtrain[,s],Predictor2=xtrain[,k])
    model3<-glm(Outcome~Predictor+Predictor2,data=yy3, family=binomial(link='logit'))
    new3<-data.frame(Predictor=xtest[,s],Predictor2=xtest[,k])
    p3<-predict(model3,new3,type="response")
    new5<-data.frame(Outcome=ytest)
    pr <- prediction(p3, new5)
    Acc <- performance(pr, measure="acc")
    AccV<-Acc@y.values[[1]][max(which(Acc@x.values[[1]] >= 0.5))]
    Sens <- performance(pr, measure= "sens")
    SensV<-Sens@y.values[[1]][max(which(Sens@x.values[[1]] >= 0.5))]
    Spec <- performance(pr, measure= "spec")
    SpecV<-Spec@y.values[[1]][max(which(Spec@x.values[[1]] >= 0.5))]
    Prec <- performance(pr, measure= "prec")
    PrecV<-Prec@y.values[[1]][max(which(Prec@x.values[[1]] >= 0.5))]
```

```
    AllV<-data.frame(Vector=c(AccV, SensV, SpecV, PrecV))
    return(AllV)
}

doubleROCfun <- function(xtrain, ytrain,xtest,ytest,s,k) {

    yy3<-data.frame(Outcome=ytrain, Predictor=xtrain[,s],Predictor2=xtrain[,k])
    model3<-glm(Outcome~Predictor+Predictor2,data=yy3, family=binomial(link='logit'))
    new3<-data.frame(Predictor=xtest[,s],Predictor2=xtest[,k])
    p3<-predict(model3,new3,type="response")
    new5<-data.frame(Outcome=ytest)
    pr3 <- prediction(p3, new5)
    prf3 <- performance(pr3, measure = "tpr", x.measure = "fpr")
    result<-prf3
    auc3 <- performance(pr3, measure = "auc")
    auc3 <- auc3@y.values[[1]]
    return(result)

}

MeansNames <- function(doubleAUCR,trial){

    doubleAUCR<-as.data.frame(doubleAUCR)
    doubleAUCR<-mutate(doubleAUCR, Means=rowMeans(doubleAUCR))
    row.names(doubleAUCR)<-trial
    return(doubleAUCR)
}

multipleAUCfun <- function(xtrain,ytrain,xtest,ytest) {

    yy3<-data.frame(Outcome=ytrain,xtrain)
    model3<-glm(Outcome~.,data=yy3, family=binomial(link='logit'))
    new3<-data.frame(xtest)
    p3<-predict(model3,new3,type="response")
    new5<-data.frame(Outcome=ytest)
    pr3 <- prediction(p3, new5)
    prf3 <- performance(pr3, measure = "tpr", x.measure = "fpr")
    auc3 <- performance(pr3, measure = "auc")
    auc3 <- auc3@y.values[[1]]
    result2<-auc3
    return(result2)
}

multipleAUCfunNB <- function(xtrain, ytrain,xtest,ytest) {

    yy3<-data.frame(Outcome=ytrain,xtrain)
    model4<-naiveBayes(Outcome~.,data=yy3)
    new3<-data.frame(xtest)
    p4<-predict(model4,new3,type="raw")
    #without raw write confusion matrix directly
    prob2<-NULL
    for (i in 1:dim(p4)[1]){
        prob2[i]<-p4[i,2]/p4[i,1]
```

```
    }
    new5<-data.frame(Outcome=ytest)
    pr3 <- prediction(prob2, new5)
    prf3 <- performance(pr3, measure = "tpr", x.measure = "fpr")
    auc3 <- performance(pr3, measure = "auc")
    auc3 <- auc3@y.values[[1]]
    result2<-auc3
    return(result2)
}

multipleROCfun <- function(xtrain,ytrain,xtest,ytest) {

    yy3<-data.frame(Outcome=ytrain,xtrain)
    model3<-glm(Outcome~.,data=yy3, family=binomial(link='logit'))
    new3<-data.frame(xtest)
    p3<-predict(model3,new3,type="response")
    new5<-data.frame(Outcome=ytest)
    pr3 <- prediction(p3, new5)
    prf3 <- performance(pr3, measure = "tpr", x.measure = "fpr")
    auc3 <- performance(pr3, measure = "auc")
    auc3 <- auc3@y.values[[1]]
    result2<-auc3
    return(prf3)

}

multipleROCfunNB <- function(xtrain, ytrain,xtest,ytest) {

    yy3<-data.frame(Outcome=ytrain,xtrain)
    model4<-naiveBayes(Outcome~.,data=yy3)
    new3<-data.frame(xtest)
    p4<-predict(model4,new3,type="raw")
    #without raw write confusion matrix directly
    prob2<-NULL
    for (i in 1:dim(p4)[1]){
        prob2[i]<-p4[i,2]/p4[i,1]
    }
    new5<-data.frame(Outcome=ytest)
    pr3 <- prediction(prob2, new5)
    prf3 <- performance(pr3, measure = "tpr", x.measure = "fpr")
    auc3 <- performance(pr3, measure = "auc")
    auc3 <- auc3@y.values[[1]]
    result2<-auc3
    return(prf3)
}

singleAUCfun <- function(xtrain, ytrain,xtest,ytest,s) {

    yy2<-data.frame(Outcome=ytrain, Predictor=xtrain[,s])
    model2<-glm(Outcome~Predictor,data=yy2,family=binomial(link='logit'))
    new2<-data.frame(Predictor=xtest[,s])
    p<-predict(model2,new2,type = "response")
    new4<-data.frame(Outcome=ytest)
```

```
    pr <- prediction(p, new4)
    prf <- performance(pr, measure = "tpr", x.measure = "fpr")
    auc <- performance(pr, measure = "auc")
    auc <- auc@y.values[[1]]
    return(auc)
}

singlePlusfun <- function(xtrain, ytrain,xtest,ytest,s) {


    yy2<-data.frame(Outcome=ytrain, Predictor=xtrain[,s])
    model2<-glm(Outcome~Predictor,data=yy2,family=binomial(link='logit'))
    new2<-data.frame(Predictor=xtest[,s])
    p<-predict(model2,new2,type = "response")
    new4<-data.frame(Outcome=ytest)
    pr <- prediction(p, new4)
    Acc <- performance(pr, measure="acc")
    AccV<-Acc@y.values[[1]][max(which(Acc@x.values[[1]] >= 0.5))]
    Sens <- performance(pr, measure= "sens")
    SensV<-Sens@y.values[[1]][max(which(Sens@x.values[[1]] >= 0.5))]
    Spec <- performance(pr, measure= "spec")
    SpecV<-Spec@y.values[[1]][max(which(Spec@x.values[[1]] >= 0.5))]
    Prec <- performance(pr, measure= "prec")
    PrecV<-Prec@y.values[[1]][max(which(Prec@x.values[[1]] >= 0.5))]
    AllV<-data.frame(Vector=c(AccV, SensV, SpecV, PrecV))
    return(AllV)
}

singleROCfun <- function(xtrain, ytrain,xtest,ytest,s) {

    yy2<-data.frame(Outcome=ytrain, Predictor=xtrain[,s])
    model2<-glm(Outcome~Predictor,data=yy2,family=binomial(link='logit'))
    new2<-data.frame(Predictor=xtest[,s])
    p<-predict(model2,new2,type = "response")
    new4<-data.frame(Outcome=ytest)
    pr <- prediction(p, new4)
    prf <- performance(pr, measure = "tpr", x.measure = "fpr")
    resultU2<-prf
    tomeany<-as.data.frame(prf@y.values)
    tomeanx<-as.data.frame(prf@x.values)
    auc <- performance(pr, measure = "auc")
    auc <- auc@y.values[[1]]
    return(resultU2)

}





set.seed(132)
```

```
### Load AML Data
setwd("/Users/j.williams/AnimeshReview/LauraPaper/Journal of Translational Medicine /LauraFinal/Sept18_
All2 <- readRDS("All2.AML.rds"); savee <- readRDS("savee.AML.rds")




#TRAIN

### set n to 10 for now to make quicker
n<-100 #Number of LASSO and EN loops. Per loop a model with features selected. The 80% most popular fea

#N and nn control the number of models created for combinatorial analysis to help in further feature se
N <-10
nn <-25

ErrorsFinEN<-vector(mode="double", length=n)
BetasFinEN<-vector(mode="character", length=n)
LambdaFinEN<-vector(mode="double", length=n)
BNumFinEN<-vector(mode="double", length=n)
see2EN<-data.frame(All="All")
LauCoef1<-data.frame(Coeff="See",stringsAsFactors=FALSE)
BetasTodo<-data.frame(Features="Name",Coefficients=1)

ListError<-vector(mode="double", length=n)
BetasFin<-vector(mode="character", length=n)
LambdaFin<-vector(mode="double", length=n)
BNumFin<-vector(mode="double", length=n)
see2<-data.frame(All="All")
LauCoef1L<-data.frame(Coeff="See",stringsAsFactors=FALSE)
BetasTodoL<-data.frame(Features="Name",Coefficients=1)

##### End Block 1
```

```
##### Begin Block 2


for (i in 1:n){

  smp_size = floor(0.75 * nrow(All2))

  train_ind <- caret::createDataPartition(All2$Label, p = 0.75, list = FALSE, times = 1)

  train = All2[train_ind, ]

  #Test set
  test = All2[-train_ind, ]

  #Creates matrices for independent and dependent variables.
  xtrain <- train[ , !(names(train) %in% "Label")] %>% as.matrix()
  ytrain = train$Label
  xtest <- test[ , !(names(test) %in% "Label")] %>% as.matrix()
```

```
ytest = test$Label


#Choose lambda value that minimize missclassification error.
#0.5 as elastic nets, all variables with EN are based on ElasticNets analysis. 100 lambdas sampled wi
CVEN=cv.glmnet(xtrain,ytrain,family="binomial",type.measure="class",alpha=0.5,nlambda=100)
attach(CVEN)
Lambda.BestEN<-CVEN$lambda.min #can be either minimum or 1 standard deviation
print(Lambda.BestEN)

CVFinEN=glmnet(xtrain,ytrain,family="binomial",alpha=0.5,lambda=Lambda.BestEN)
CoefEN<-coef(CVFinEN) #Beta coefficients obtained from here
InterceptEN<-CoefEN@x[1]
BetasEN<-CVFinEN$beta
Betas2EN<-data.frame(Features=BetasEN@Dimnames[[1]][BetasEN@i+1], Coefficients=BetasEN@x) #Beta coeff
CVPred1EN = predict(CVFinEN, family="binomial", s=Lambda.BestEN, newx = xtest,type="class") #predict

#Calculate error for categorical values
ytest2<-as.factor(ytest)
ResultsEN<-table(CVPred1EN,ytest)
confusionMatrix(as.factor(CVPred1EN),ytest)
AccuracyEN<-(ResultsEN[1]+ResultsEN[4])/sum(ResultsEN[1:4])
ErrorEN<-1-AccuracyEN

LauCoef<-Betas2EN$Coefficients
LauCoefEN<-data.frame(Coeff=LauCoef,stringsAsFactors=FALSE)
LauCoef1<-rbind(LauCoef1,LauCoefEN)
BetasTodo<-rbind(BetasTodo,Betas2EN) #store coefficients and store betas

seeEN<-Betas2EN$Features
seeEN1<-data.frame(All=seeEN)
see2EN<-rbind(see2EN,seeEN1)   #all beta names stored


mEN<-count(see2EN, All) #frequency of the betas stored counted
see3EN<-toString(seeEN)
ErrorsFinEN[i]<-ErrorEN #error of the model stored
BetasFinEN[i]<-see3EN #name of features the model used
BNumFinEN[i]<-length(seeEN) #number of features the model used
LambdaFinEN[i]<-Lambda.BestEN #lambda chosen for model
detach(CVEN)

#Change between Lasso and EN, alpha=1 (*)
CV=cv.glmnet(xtrain,ytrain,family="binomial",type.measure="class",alpha=1,nlambda=100)

attach(CV)

Lambda.Best<-CV$lambda.min
CVFin=glmnet(xtrain,ytrain,family="binomial",alpha=1,lambda=Lambda.Best)
Coef<-coef(CVFin)
Intercept<-Coef@x[1]
Betas<-CVFin$beta
Betas2<-data.frame(Features=Betas@Dimnames[[1]][Betas@i+1], Coefficients=Betas@x)
```

```r
  CVPred1 = predict(CVFin, family="binomial", s=Lambda.Best, newx = xtest,type="class")


  #Calculate error for categorical values
  ytest2<-as.factor(ytest)
  confusionMatrix(as.factor(CVPred1),ytest)
  Results<-table(CVPred1,ytest)
  Accuracy<-(Results[1]+Results[4])/sum(Results[1:4])
  Error<-1-Accuracy

  #visual display of for

  BetasTodoL<-rbind(BetasTodoL,Betas2)
  see<-Betas2$Features
  see1<-data.frame(All=see)
  see2<-rbind(see2,see1)
  m<-count(see2, All)

  see3<-toString(see)
  ListError[i]<-Error
  BetasFin[i]<-see3
  BNumFin[i]<-length(see)
  LambdaFin[i]<-Lambda.Best
  detach(CV)

}
```

```
## [1] 0.01410672
## [1] 0.06136322
## [1] 0.0572892
## [1] 0.02276051
## [1] 0.0768437
## [1] 0.01208532
## [1] 0.09287787
## [1] 0.08556725
## [1] 0.06002755
## [1] 0.0477804
## [1] 0.04394421
## [1] 0.09268814
## [1] 0.06570016
## [1] 0.03319311
## [1] 0.05911919
## [1] 0.02889832
## [1] 0.08646156
## [1] 0.008838517
## [1] 0.05092736
## [1] 0.01965162
## [1] 0.0620856
## [1] 0.09770617
## [1] 0.09408764
## [1] 0.07763139
## [1] 0.08480714
## [1] 0.0689899
## [1] 0.07218094
```

```
## [1] 0.07332494
## [1] 0.0751107
## [1] 0.05546047
## [1] 0.03706898
## [1] 0.01768615
## [1] 0.07290411
## [1] 0.02436325
## [1] 0.1010492
## [1] 0.07947772
## [1] 0.08638957
## [1] 0.06704386
## [1] 0.005414587
## [1] 0.07849055
## [1] 0.06146099
## [1] 0.03060099
## [1] 0.01808845
## [1] 0.09695372
## [1] 0.08502742
## [1] 0.06793977
## [1] 0.08769333
## [1] 0.06770055
## [1] 0.07696627
## [1] 0.02821053
## [1] 0.01849147
## [1] 0.08380528
## [1] 0.07297679
## [1] 0.006236947
## [1] 0.03218741
## [1] 0.100343
## [1] 0.05928835
## [1] 0.0692829
## [1] 0.06440835
## [1] 0.05934364
## [1] 0.08362592
## [1] 0.0537196
## [1] 0.009768704
## [1] 0.08008044
## [1] 0.02443207
## [1] 0.07740715
## [1] 0.01072579
## [1] 0.1275876
## [1] 0.0206432
## [1] 0.1033139
## [1] 0.005817232
## [1] 0.09127219
## [1] 0.0771582
## [1] 0.07655367
## [1] 0.009287085
## [1] 0.07404753
## [1] 0.037219
## [1] 0.01361882
## [1] 0.06059189
## [1] 0.01232926
## [1] 0.01386188
```

```
## [1] 0.09655957
## [1] 0.05262116
## [1] 0.00697718
## [1] 0.05080445
## [1] 0.05765488
## [1] 0.0942935
## [1] 0.02991813
## [1] 0.08112793
## [1] 0.0526637
## [1] 0.07080803
## [1] 0.0766287
## [1] 0.06940108
## [1] 0.08790559
## [1] 0.07147071
## [1] 0.009480638
## [1] 0.1038332
## [1] 0.01677133
## [1] 0.1024412
## [1] 0.02911092
```

```
#Visualizing data from LASSO and EN ####


#obtain in a data frame all error, betas names, number and lamda for the N models for each lasso and EN
All_info<-data.frame(Error=ListError, BetasNames=BetasFin, BetasNum=BNumFin, Lambda=LambdaFin)
All_infoEN<-data.frame(Error=ErrorsFinEN, BetasNames=BetasFinEN, BetasNum=BNumFinEN, Lambda=LambdaFinEN

m<-m[-1,]
mEN<-mEN[-1,]

Final_LASSO<-m[order(-m$n),] #order highest frequencies above and filter with those that appear more th
Final_LASSO1<-filter(Final_LASSO,n>40) #threshold selected - 80%



Final_EN<-mEN[order(-mEN$n),]
Final_EN1<-filter(Final_EN,n>40)
Final_Plot_Names<-filter(Final_EN,n>40)

outputVenn2<-venn(list(EN= Final_EN$All, LASSO = Final_LASSO$All))
```

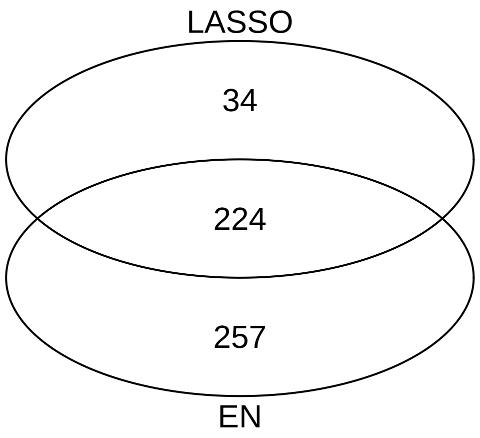


```
outputVenn<-venn(list(EN= Final_EN1$All, LASSO = Final_LASSO1$All))
```

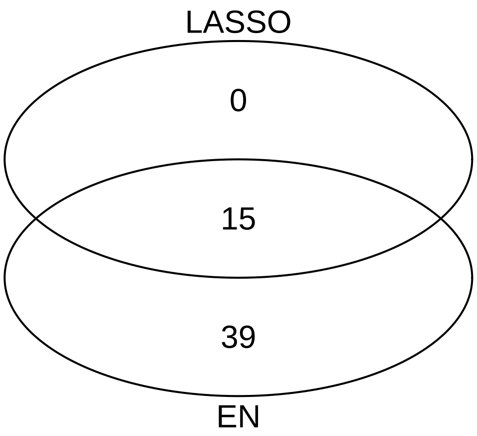


```
Freqs<-m[order(-m$n),]
num<-length(Freqs$All)

Freqs$All <- factor(Freqs$All, levels = Freqs$All[order(-Freqs$n)]) #plot in a bar graph the frequencie
ggplot(Freqs, aes(All, n))+geom_bar(stat="identity")+theme(axis.text.x = element_text(size=8, angle=90))
```

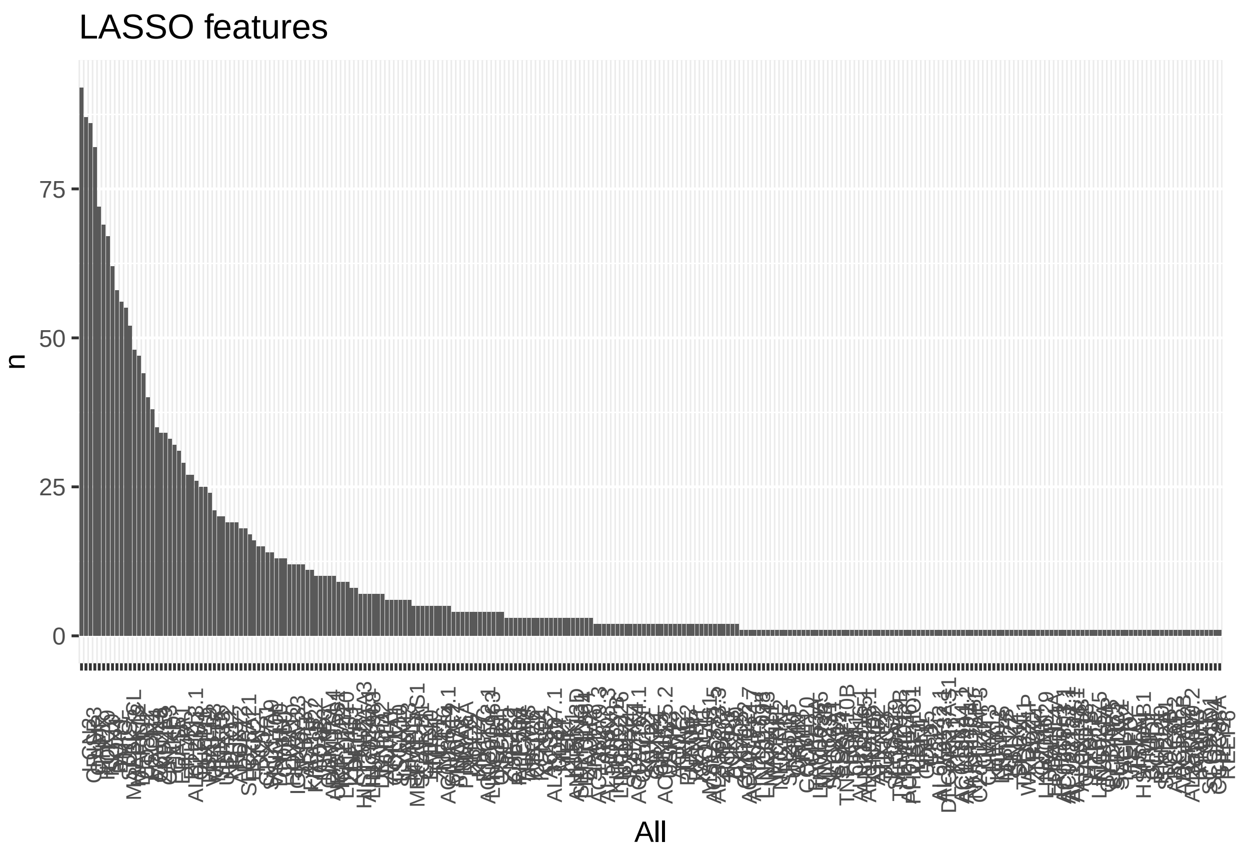


```
FreqsEN<-mEN[order(-mEN$n),]
numEN<-length(FreqsEN$All)

FreqsEN$All <- factor(FreqsEN$All, levels = FreqsEN$All[order(-FreqsEN$n)])
ggplot(FreqsEN, aes(All, n))+geom_bar(stat="identity")+theme(axis.text.x = element_text(size=8, angle=9
```

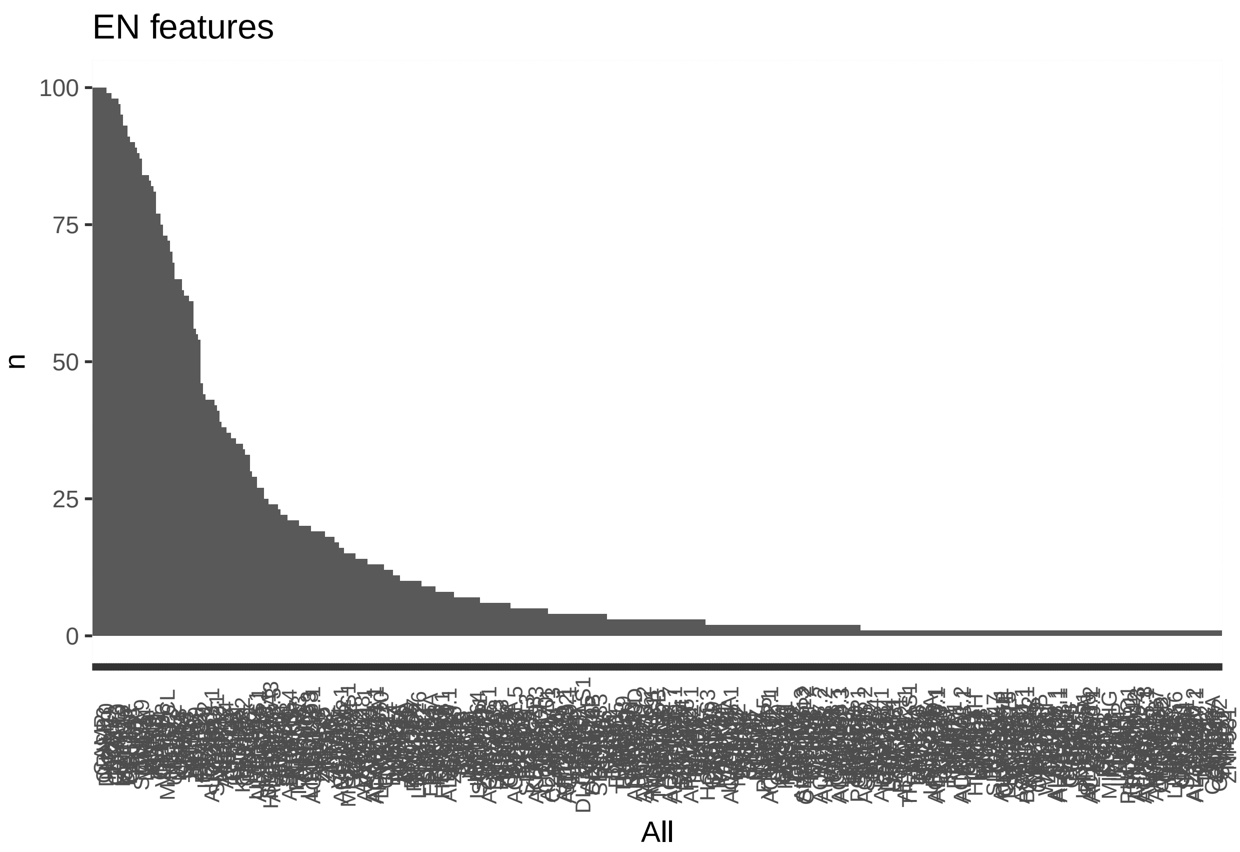


```
#plot of how many times each feature appears. Most important will appear in all models = N.


#Boxplot with Betas and its coefficients

Boxplot1<-BetasTodo[BetasTodo$Features %in% Final_EN1$All,] #see which features appear in the filtered
ggplot(Boxplot1,aes(Boxplot1$Features,Boxplot1$Coefficients))+geom_boxplot()+geom_jitter()
```

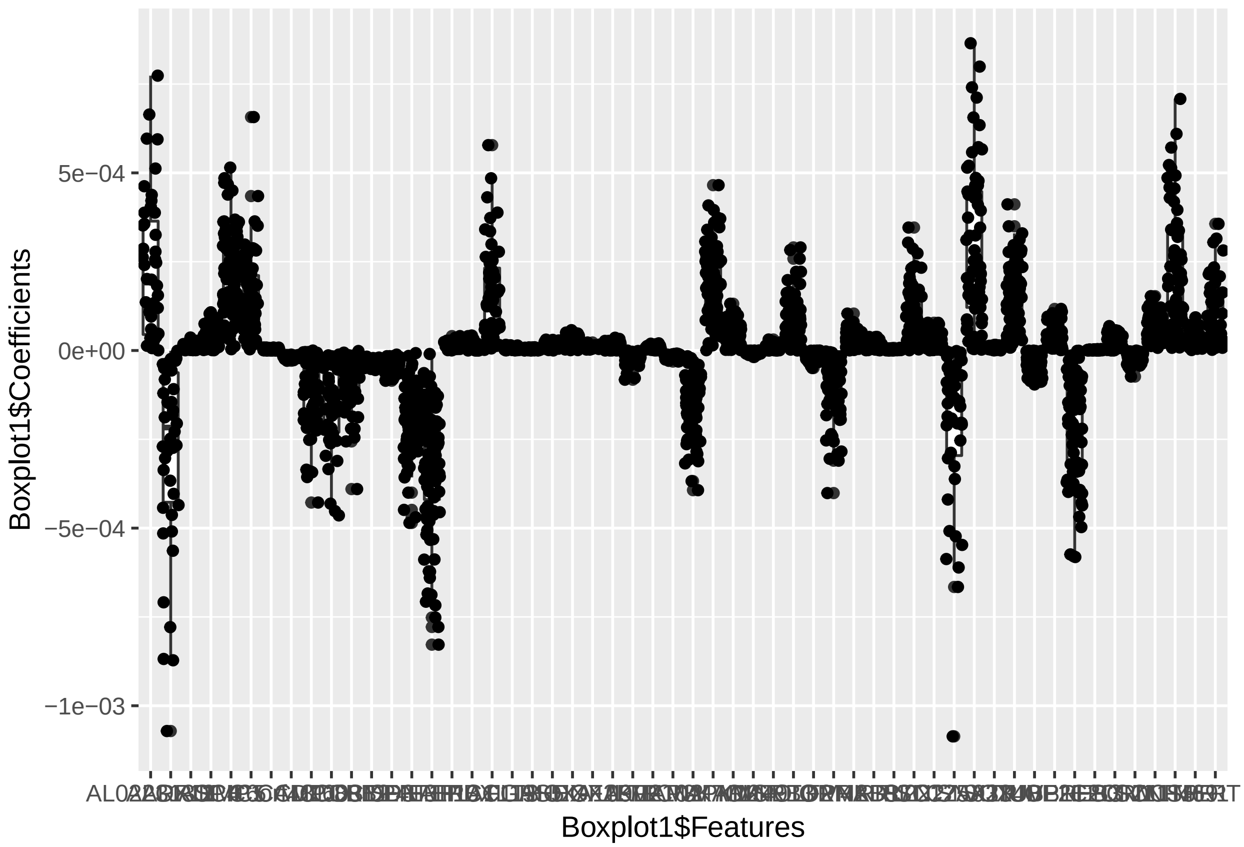


```
Boxplot1["Method"]<-as.factor("EN")

Boxplot2<-BetasTodoL[BetasTodoL$Features %in% Final_LASSO1$All,]
ggplot(Boxplot2,aes(Boxplot2$Features,Boxplot2$Coefficients))+geom_boxplot()+geom_jitter()
```

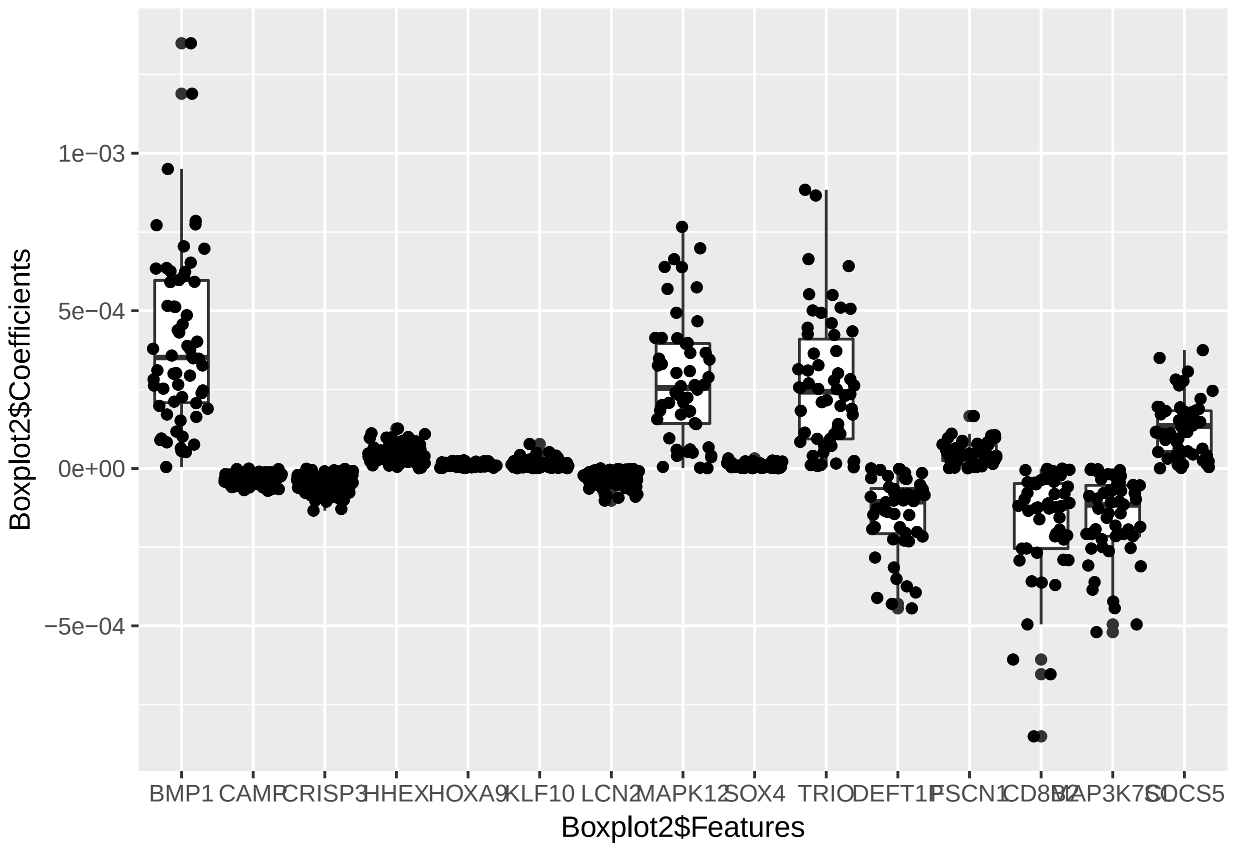


```
Boxplot2["Method"]<-as.factor("LASSO")

Fin_Boxplot<-rbind(Boxplot1,Boxplot2) #Unite both boxplots LASSO and EN
ggplot(Fin_Boxplot,aes(Fin_Boxplot$Features,Fin_Boxplot$Coefficients))+geom_boxplot(aes(color=Method))+g
```

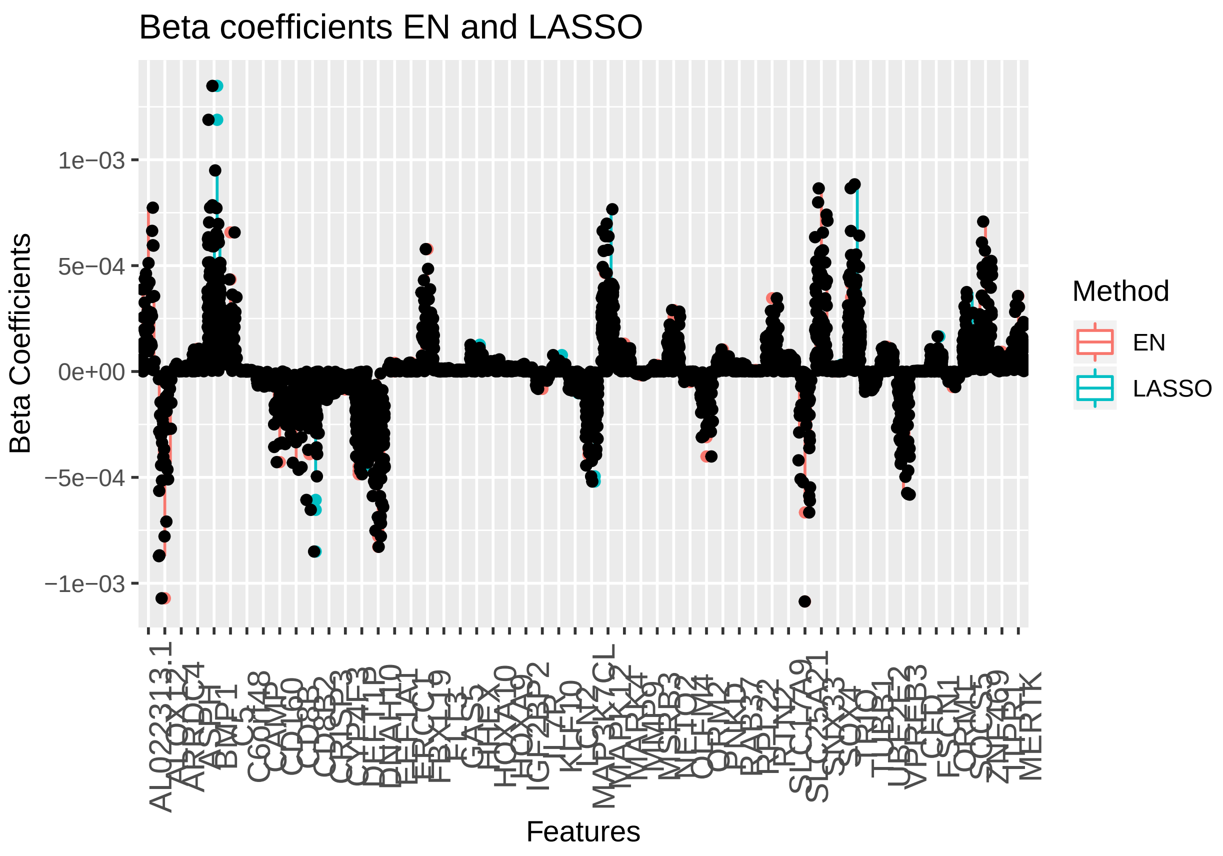


```
All_Feat<-rbind(Final_LASSO1,Final_EN1)
All_Feat2<-unique(All_Feat$All) #select the filtered betas in both.
#All_Feat2<-data.frame(All_Feat2)
Betas_select<-All2[,colnames(All2[,intersect(gsub("`", "", All_Feat2), colnames(All2))])]
Betas_select["Label"]<-All2$Label
Betas_select<-data.frame(Betas_select)

#Beta_select are the final features selected.

##### End Block 2
```

```
##### Begin Block 3

###Combinatorial analysis ####
NumVar<-length(Betas_select)
maxCombF<-data.frame(Name=toString("Trial"),AUC=0)
auc3max<-0
cont<-0
cont2<-0


for (p in 1:N){ #N different measurements of AUC values, mean done at the end.
  smp_size<-floor(0.65 * nrow(Betas_select))
  train_ind <- caret::createDataPartition(All2$Label, p = 0.65, list = FALSE, times = 1)

  # Training set
  train<-Betas_select[train_ind, ]
```

```r
# Test set
test<-Betas_select[-train_ind, ]

xtrain <- train[ , !(names(train) %in% "Label")] %>% as.matrix()

xtest <- test[ , !(names(test) %in% "Label")] %>% as.matrix()

ytrain<-train$Label
ytest<-test$Label

xtest<-data.frame(xtest)
xtrain<-data.frame(xtrain)

y<-Betas_select[,NumVar]
X<-Betas_select[,1:(NumVar-1)]


levels(ytrain)[1]<-"0"
levels(ytrain)[2]<-"1"
levels(ytest)[1]<-"0"
levels(ytest)[2]<-"1"


for (k in 1:nn){
  columns<-c(1:dim(xtrain)[2])
  columns<-sample(columns)
  d<-xtrain[,columns]
  for (i in 1:dim(xtrain)[2]){
    for (j in 1:dim(xtrain)[2]){
      yy3<-data.frame(Outcome=ytrain,d[i:j])
      model3<-glm(Outcome~.,data=yy3, family=binomial(link='logit'))
      dd<-xtest[,columns]
      new3<-data.frame(dd[i:j])
      p3<-predict(model3,new3,type="response")
      new5<-data.frame(Outcome=ytest)
      pr3 <- prediction(p3, new5)
      prf3 <- performance(pr3, measure = "tpr", x.measure = "fpr")
      auc3 <- performance(pr3, measure = "auc")
      auc3 <- auc3@y.values[[1]]
      result2<-auc3
      if (auc3>auc3max){
        if (i>j){
          maxComb<-data.frame(Name=toString(names(d)[j:i]),AUC=auc3)
          auc3max<-auc3
          cont<-cont+1
        }
        else{

          maxComb<-data.frame(Name=toString(names(d)[i:j]),AUC=auc3)
          auc3max<-auc3
          cont<-cont+1
        }
      }
```

```
        else{
          cont2<-cont2+1
        }

      }
    }
  }
  maxCombF<-rbind(maxCombF,maxComb)
  auc3max<-0
}

maxCombF2<-maxCombF[order(maxCombF$AUC),]
names<-maxCombF2$Name[dim(maxCombF2)[1]]
names1<-as.character(names)
names1<-strsplit(names1,", ")
names<-as.data.frame(names1)

Betas_select2<-All2[,colnames(All2[,intersect(gsub("`", "", names[,1]), colnames(All2))])]
Betas_select2["Label"]<-savee
Betas_select2<-as.data.frame(Betas_select2)

##### End Block 3
```

```
##### Begin Block 4


#Random mix between variables and AUC value obtained through GLM model, rough approximation of future p
#Betas_select2, final features selected.


NumVar<-length(Betas_select2)


N<-1000 #number of models produced  (both permuted (random) and real) - for all univariate (Each featur
#Real program runs with 1000 - Takes 12 hours.

multipleAUC<-matrix(rnorm(2),1,N)
multipleAUCR<-matrix(rnorm(2),1,N)
multipleAUCNB<-matrix(rnorm(2),1,N)
multipleAUCNBR<-matrix(rnorm(2),1,N)

multipleROC<-matrix(as.list(rnorm(2)),1,N)
multipleROCR<-matrix(as.list(rnorm(2)),1,N)
multipleNBROC<-matrix(as.list(rnorm(2)),1,N)
multipleNBROCR<-matrix(as.list(rnorm(2)),1,N)

singleROC<-list()
doubleROC<-list()
singleROCR<-list()
doubleROCR<-list()

doublePlus<-list()
singlePlus<-list()
```

```
singleAUC<-matrix(rnorm(2),NumVar-1,N)
doubleAUC<-matrix(rnorm(2),(NumVar-1),N)
singleAUCR<-matrix(rnorm(2),NumVar-1,N)
doubleAUCR<-matrix(rnorm(2),(NumVar-1),N)

doubleAUCSVMR<-matrix(rnorm(2),(NumVar-1),N)
doubleAUCSVM<-matrix(rnorm(2),(NumVar-1),N)
doubleAUCSVMCrossR<-matrix(rnorm(2),(NumVar-1),N)
doubleAUCSVMCross<-matrix(rnorm(2),(NumVar-1),N)
doubleAUCRFCross<-matrix(rnorm(2),(NumVar-1),N)
doubleAUCRFCrossR<-matrix(rnorm(2),(NumVar-1),N)
doubleAUCNBR<-matrix(rnorm(2),(NumVar-1),N)
doubleAUCNB<-matrix(rnorm(2),(NumVar-1),N)

MatsingleROC<-matrix(as.list(rnorm(2)),NumVar-1,N)
MatsingleROCR<-matrix(as.list(rnorm(2)),NumVar-1,N)
MatdoubleROC<-matrix(as.list(rnorm(2)),(NumVar-1),N)
MatdoubleROCR<-matrix(as.list(rnorm(2)),(NumVar-1),N)

MatsinglePlus<-matrix(as.list(rnorm(2)),NumVar-1,N)
MatdoublePlus<-matrix(as.list(rnorm(2)),(NumVar-1),N)
MatsinglePlusR<-matrix(as.list(rnorm(2)),NumVar-1,N)
MatdoublePlusR<-matrix(as.list(rnorm(2)),(NumVar-1),N)
##### End Block 4
```

```
##### Begin Block 5



for (j in 1:N){ #N different measurements of AUC values, mean done at the end.
  smp_size<-floor(0.65 * nrow(Betas_select2))
  #set.seed(907)
  train_ind <- caret::createDataPartition(All2$Label, p = 0.65, list = FALSE, times = 1)

  # Training set
  train<-Betas_select2[train_ind, ]

  # Test set
  test<-Betas_select2[-train_ind, ]

  xtrain <- train[ , !(names(train) %in% "Label")] %>% as.matrix()

  xtest <- test[ , !(names(test) %in% "Label")] %>% as.matrix()

  ytrain<-train$Label
  ytest<-test$Label

  xtest<-data.frame(xtest)
  xtrain<-data.frame(xtrain)

  y<-Betas_select2[,NumVar]
  X<-Betas_select2[,1:(NumVar-1)]
```

```r
levels(ytrain)[1]<-"0"
levels(ytrain)[2]<-"1"
levels(ytest)[1]<-"0"
levels(ytest)[2]<-"1"




## multiple, good
multipleAUCNB[1,j]<-multipleAUCfunNB(xtrain, ytrain,xtest,ytest)
multipleAUC[1,j]<-multipleAUCfun(xtrain, ytrain,xtest,ytest)

multipleNBROC[[j]]<-multipleROCfunNB(xtrain, ytrain,xtest,ytest)
multipleROC[[j]]<-multipleROCfun(xtrain, ytrain,xtest,ytest)


#separate


## single, should be good
for (s in (1:(NumVar-1))){
  s<-as.numeric(s)
  singleAUC[s,j]<-singleAUCfun(xtrain, ytrain,xtest,ytest,s)
  singleROC[[s]]<-singleROCfun(xtrain, ytrain,xtest,ytest,s)
  MatsinglePlus[s,j]<-singlePlusfun(xtrain, ytrain,xtest,ytest,s)
}
MatsingleROC[,j]<-matrix(singleROC)


#Null Hypothesis ####




# Training set

train$Label<-sample(train$Label)
test$Label<-sample(test$Label)
#Permuted data, will make sure that are models are really valid as randomizing the label should yield

# Test set

xtrain <- train[ , !(names(train) %in% "Label")] %>% as.matrix()

xtest <- test[ , !(names(test) %in% "Label")] %>% as.matrix()

ytrain<-train$Label
ytest<-test$Label

xtest<-data.frame(xtest)
xtrain<-data.frame(xtrain)
```

```
  y<-Betas_select2[,NumVar]
  X<-Betas_select2[,1:(NumVar-1)]


  levels(ytrain)[1]<-"0"
  levels(ytrain)[2]<-"1"
  levels(ytest)[1]<-"0"
  levels(ytest)[2]<-"1"





  multipleAUCNBR[1,j]<-multipleAUCfunNB(xtrain, ytrain,xtest,ytest)
  multipleAUCR[1,j]<-multipleAUCfun(xtrain, ytrain,xtest,ytest)

  multipleNBROCR[[j]]<-multipleROCfunNB(xtrain, ytrain,xtest,ytest)
  multipleROCR[[j]]<-multipleROCfun(xtrain, ytrain,xtest,ytest)


  for (s in (1:(NumVar-1))){
    s<-as.numeric(s)
    singleAUCR[s,j]<-singleAUCfun(xtrain, ytrain,xtest,ytest,s)
    singleROCR[[s]]<-singleROCfun(xtrain, ytrain,xtest,ytest,s)
    MatsinglePlusR[s,j]<-singlePlusfun(xtrain, ytrain,xtest,ytest,s)
  }
  MatsingleROCR[,j]<-matrix(singleROCR)


  #
}

##### End Block 5
```

```
##### Begin Block 6

  names2<-sapply(1:(NumVar-1), function(i){paste0("NISS/",names(xtrain)[i])})


trial<-NULL

h=1
for (b in (2:NumVar-1)) {
  print(b)
  trial[b]<-names2[h]
  h=h+1
}
```

```
## [1] 1
## [1] 2
## [1] 3
## [1] 4
## [1] 5
## [1] 6
## [1] 7
```

```
## [1] 8
```

```
singleAUC<-as.data.frame(singleAUC)
singleAUC<-mutate(singleAUC, Means=rowMeans(singleAUC))
row.names(singleAUC)<-names(xtrain)


singleAUCR<-as.data.frame(singleAUCR)
singleAUCR<-mutate(singleAUCR, Means=rowMeans(singleAUCR))
row.names(singleAUCR)<-names(xtrain)

multipleAUCNBR<-as.data.frame(multipleAUCNBR)
multipleAUCNBR["Means"]<-rowMeans(multipleAUCNBR)
multipleAUCR<-as.data.frame(multipleAUCR)
multipleAUCR["Means"]<-rowMeans(as.data.frame(multipleAUCR))

multipleAUCNB<-as.data.frame(multipleAUCNB)
multipleAUCNB["Means"]<-rowMeans(as.data.frame(multipleAUCNB))
multipleAUC<-as.data.frame(multipleAUC)
multipleAUC["Means"]<-rowMeans(as.data.frame(multipleAUC))

Final<-data.frame(MultiNB=t(multipleAUCNB),MultiNBRand=t(multipleAUCNBR),Multi=t(multipleAUC),MultiRand
FinalMeans<-data.frame(MultiNB=multipleAUCNB$Means,MultiNBRand=multipleAUCNBR$Means,Multi=multipleAUC$Me

#sacar ROC CURVES #####

print("here")
```

```
## [1] "here"
```

```
for (g in 1:(NumVar-1)){

  plot(MatsingleROC[[g,1]],lwd=3,main=paste("ROC curve of", names(xtrain)[g]))
  for (b in 1:N){

    plot(MatsingleROCR[[g,b]],col=b,lty=3,add=TRUE)
  }
}
```

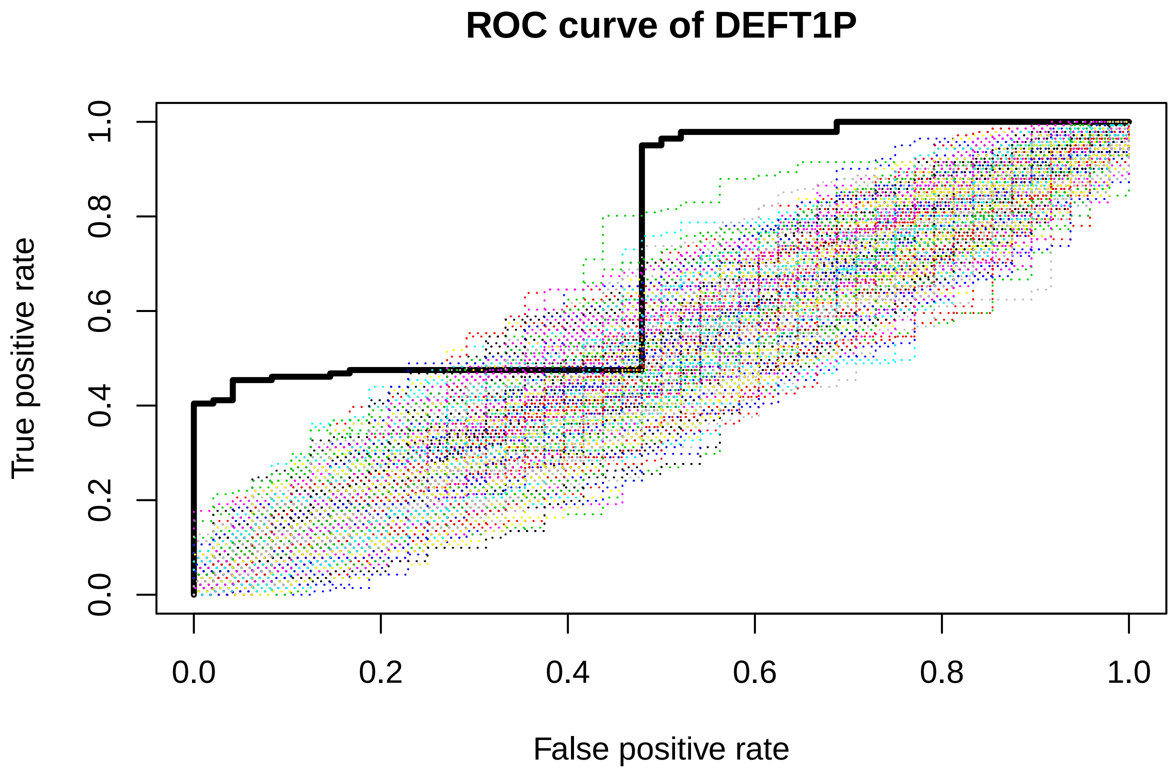
ROC curve of DEFT1P
True positive rate
0.0
0.2
0.4
0.6
0.8
1.0
False positive rate
0.0
0.2
0.4
0.6
0.8
1.0

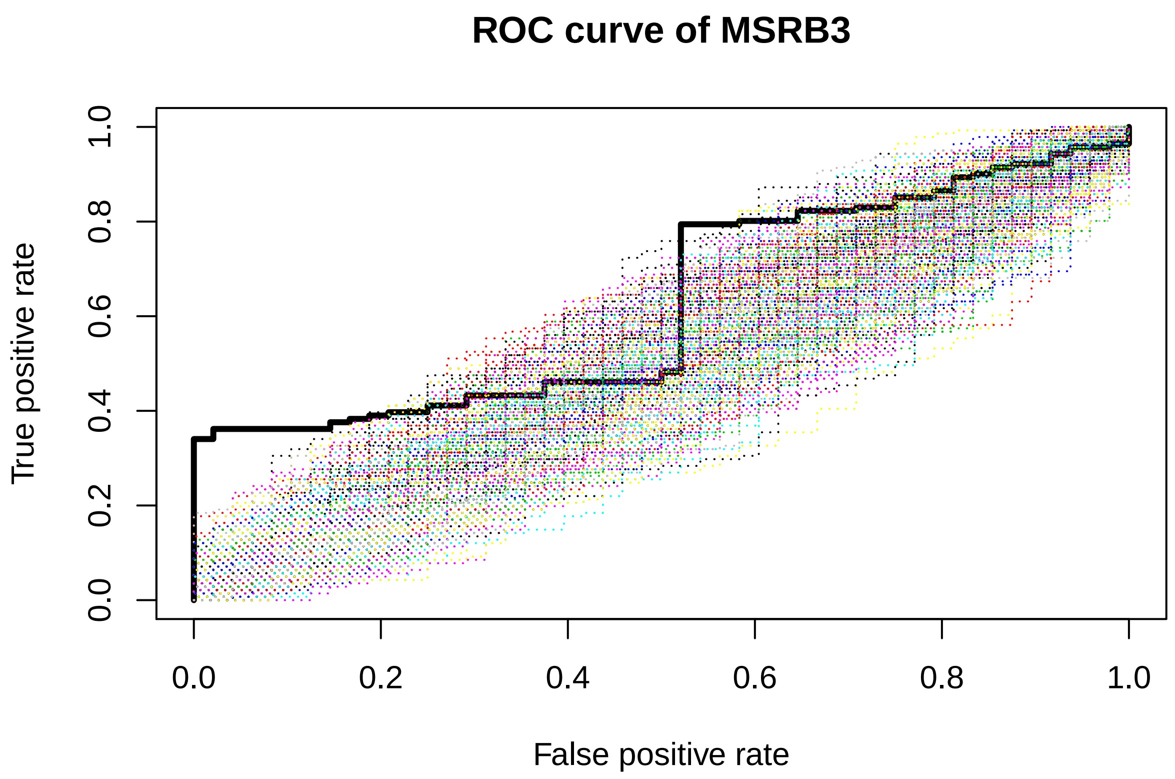
ROC curve of MSRB3
True positive rate
False positive rate
0.0
0.2
0.4
0.6
0.8
1.0
0.0
0.2
0.4
0.6
0.8
1.0

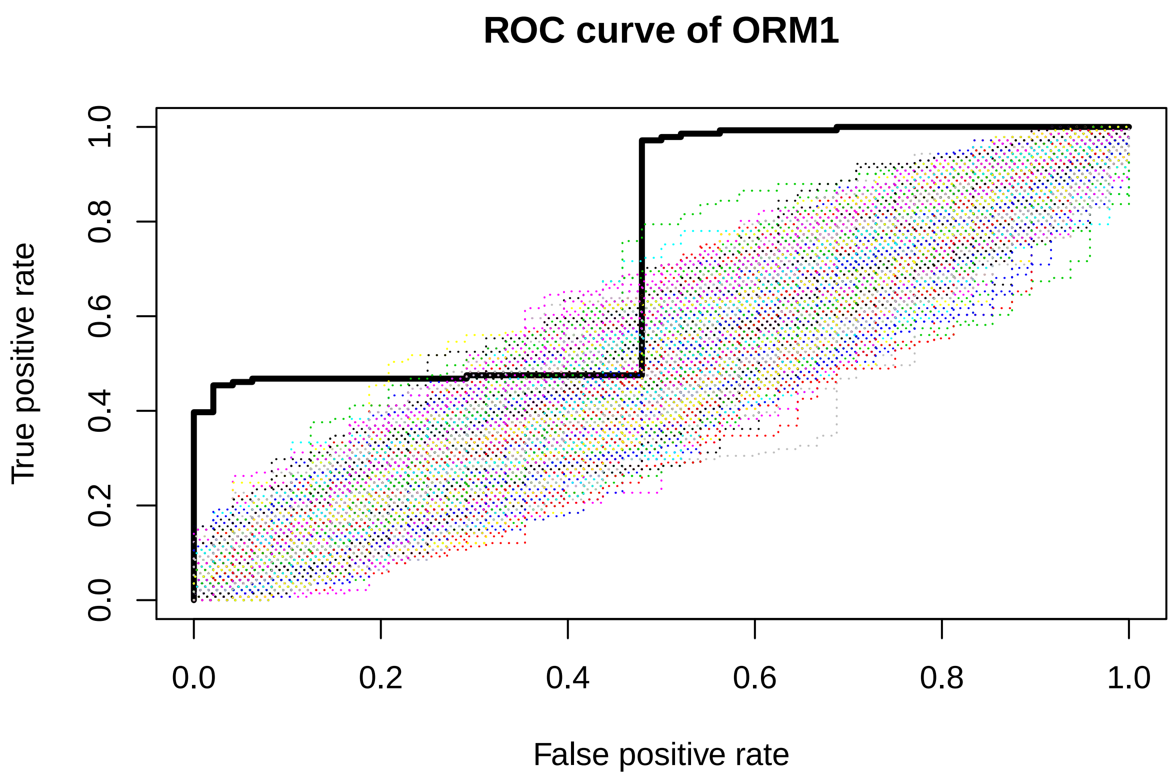
ROC curve of ORM1
True positive rate
0.0
0.2
0.4
0.6
0.8
1.0
False positive rate
0.0
0.2
0.4
0.6
0.8
1.0

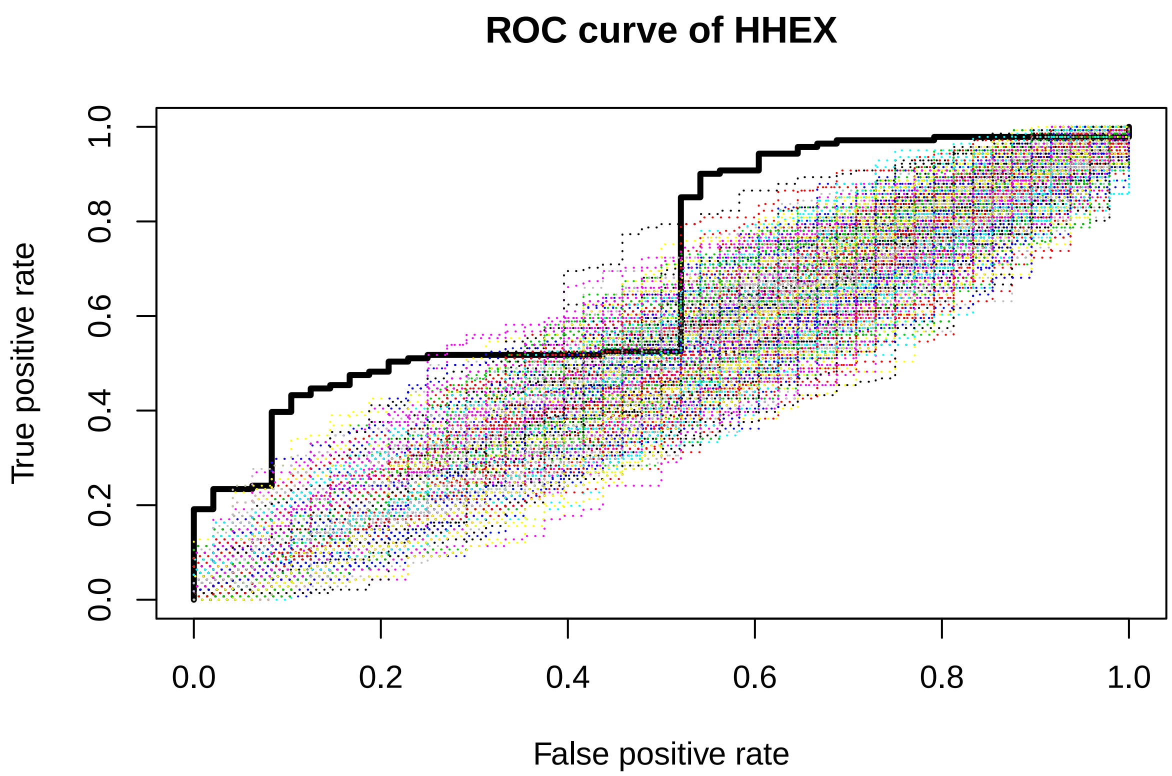
ROC curve of HHEX
True positive rate
0.0
0.2
0.4
0.6
0.8
1.0
False positive rate

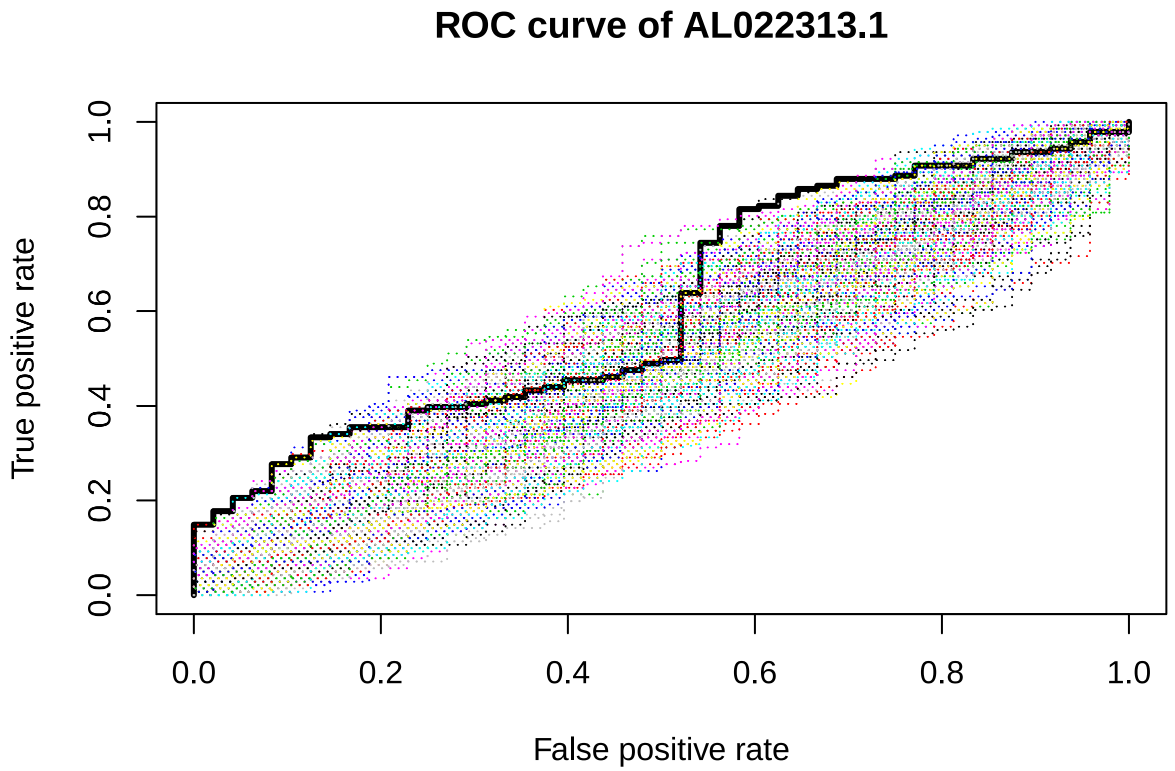

ROC curve of AL022313.1
True positive rate
False positive rate
0.0
0.2
0.4
0.6
0.8
1.0

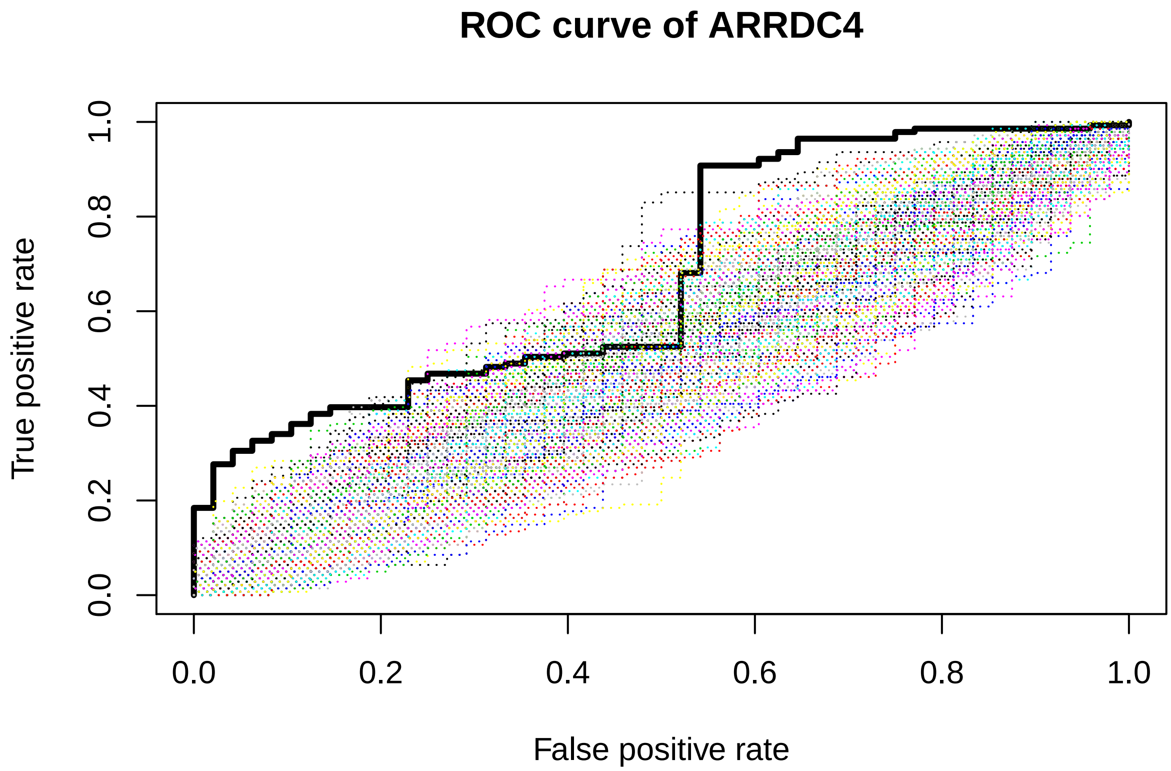
ROC curve of ARRDC4
True positive rate
0.0
0.2
0.4
0.6
0.8
1.0
False positive rate
0.0
0.2
0.4
0.6
0.8
1.0

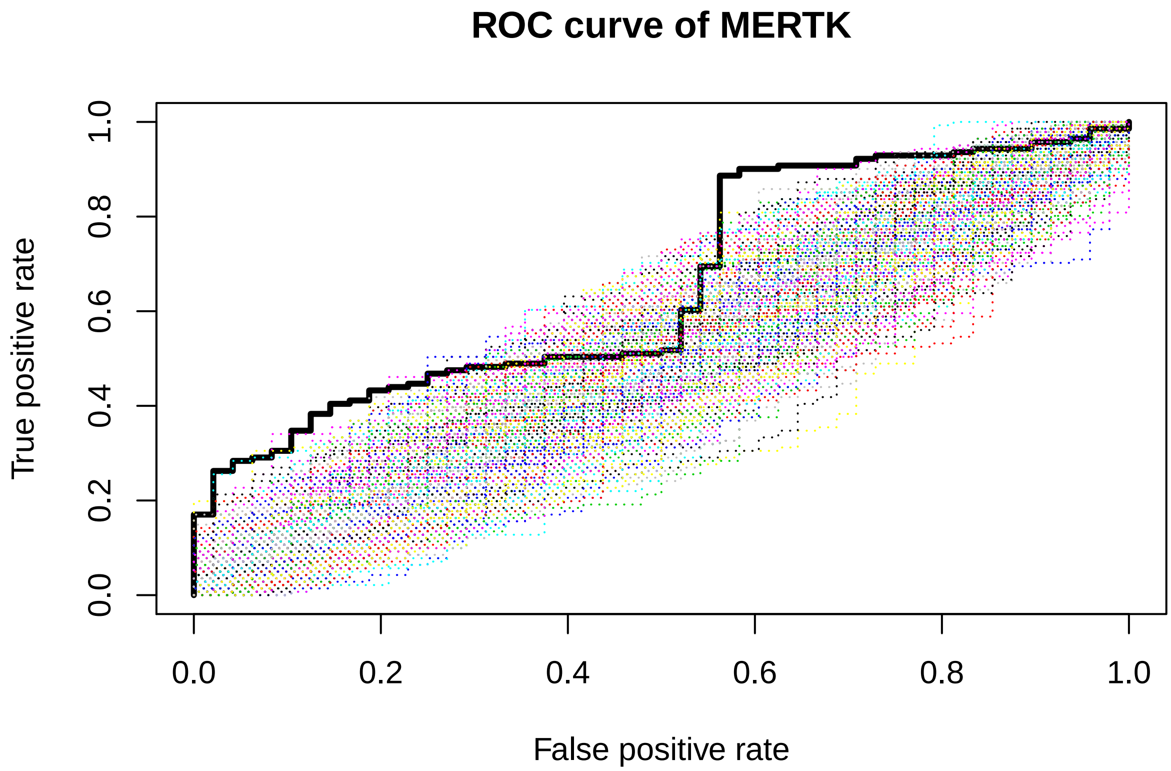
ROC curve of MERTK
True positive rate
False positive rate
0.0
0.2
0.4
0.6
0.8
1.0

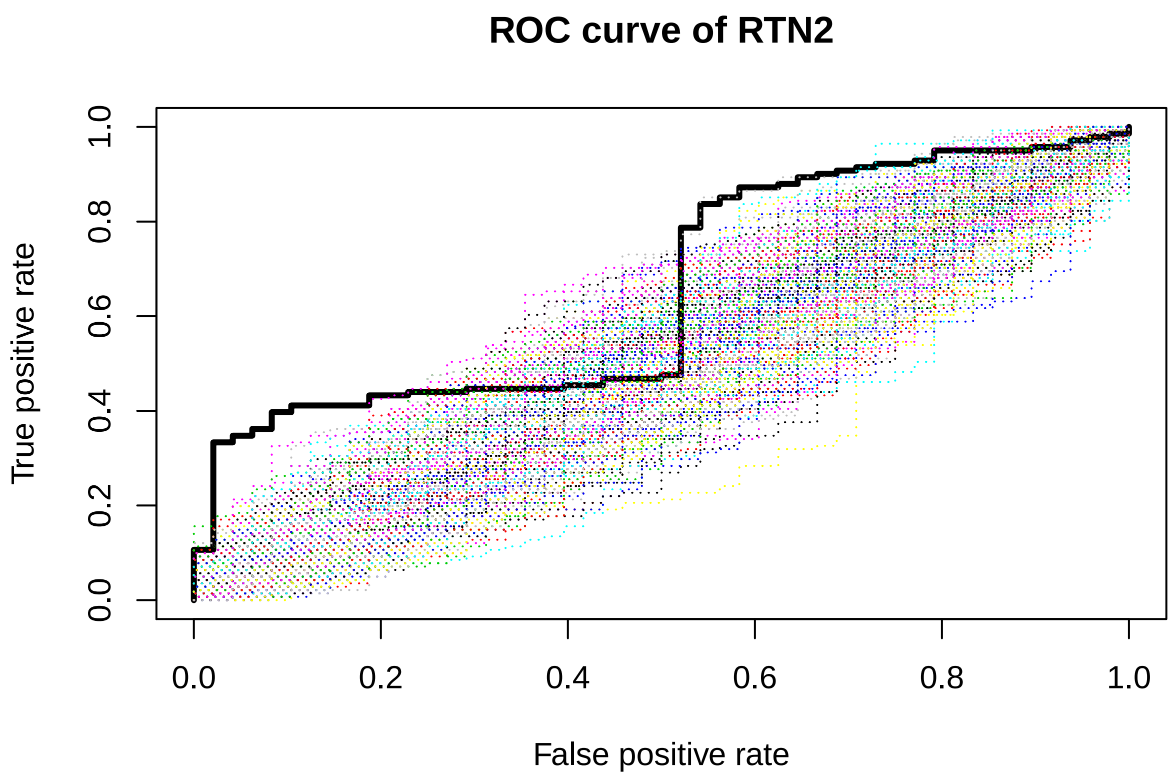


```
plot(multipleROC[[1]],lwd=3,main=paste("ROC curve of", trial[1]))
for (b in 1:N){
  plot(multipleNBROCR[[b]],col=b,lty=3,add=TRUE)
}
```

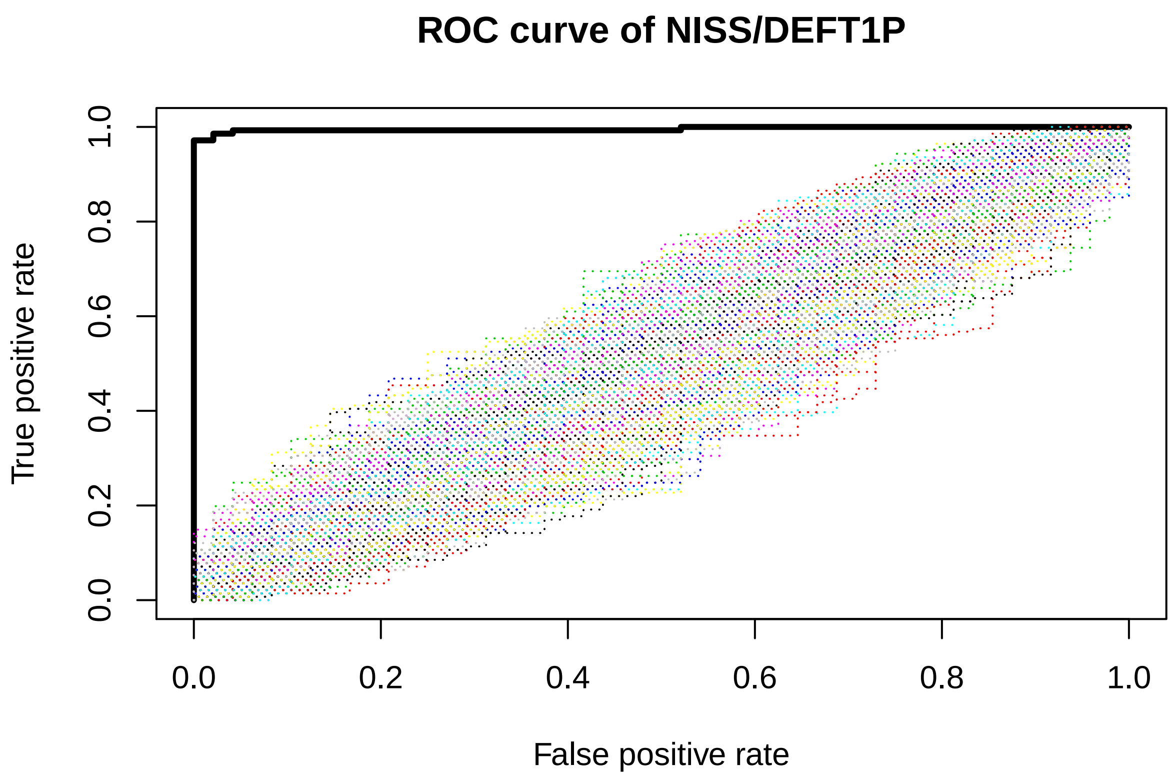


```
#
#
#

AUCT<-as.data.frame(t(singleAUC[1:dim(singleAUC)[2]-1]))
AUC2T<-as.data.frame(t(singleAUCR[1:dim(singleAUCR)[2]-1]))
one<-as.data.frame(singleAUC$Means)
two<-as.data.frame(singleAUCR$Means)
Mean<-data.frame(one,two)
Mean<-as.matrix(Mean)
#
nm<-names(AUCT)
for (j in 1:(NumVar-1)){

  Mono<-data.frame(Mono=AUCT[,j],Label=as.factor(c(rep("model",dim(AUCT)[1]))))
  Mono1<-data.frame(Mono=AUC2T[,j],Label=as.factor(c(rep("permuted data",dim(AUC2T)[1]))))
  Mono<-rbind(Mono,Mono1)
  print(ggdensity(Mono, x = "Mono", fill = "Label", palette = "jco")+geom_vline(xintercept =Mean[j,],li
}
```

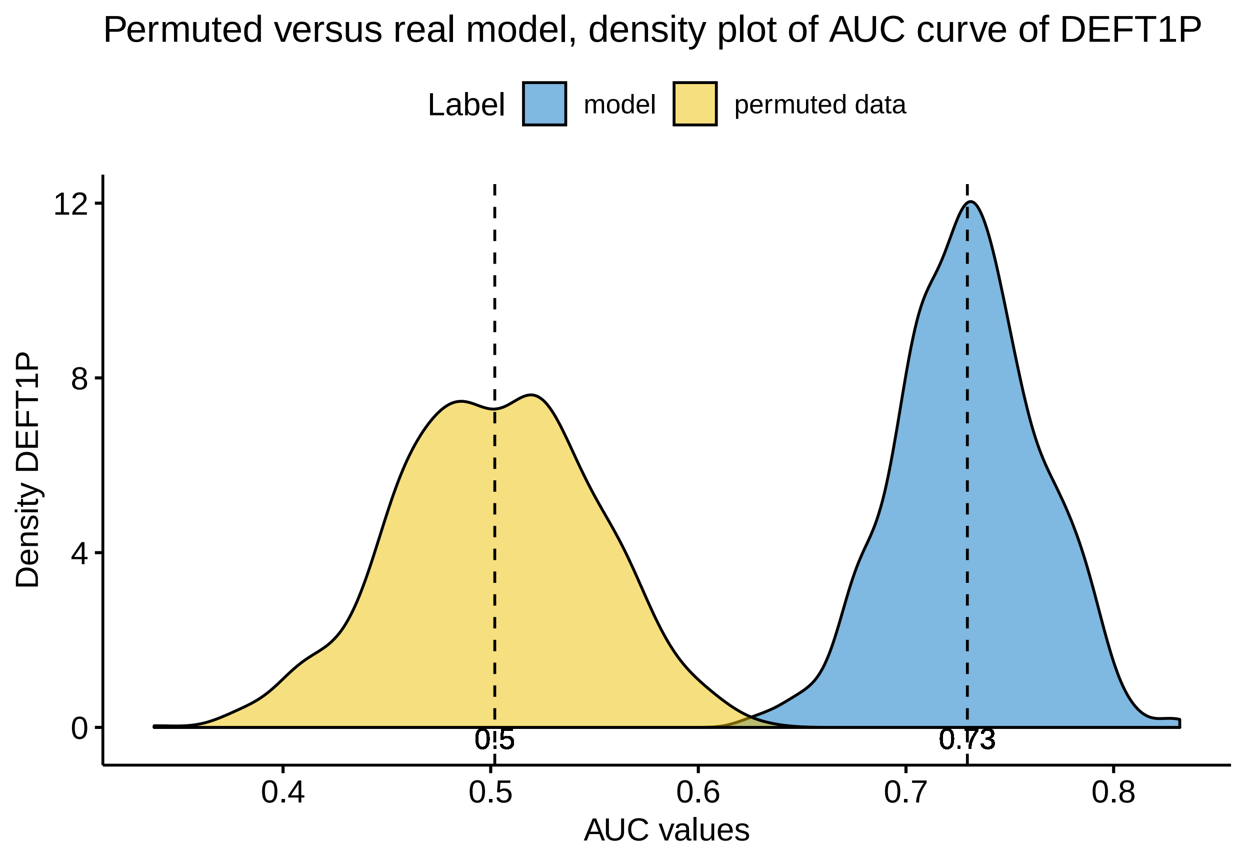

Permuted versus real model, density plot of AUC curve of DEFT1P
Label
model
permuted data
Density DEFT1P
12
8
4
0
0.5
0.73
0.4
0.5
0.6
0.7
0.8
AUC values

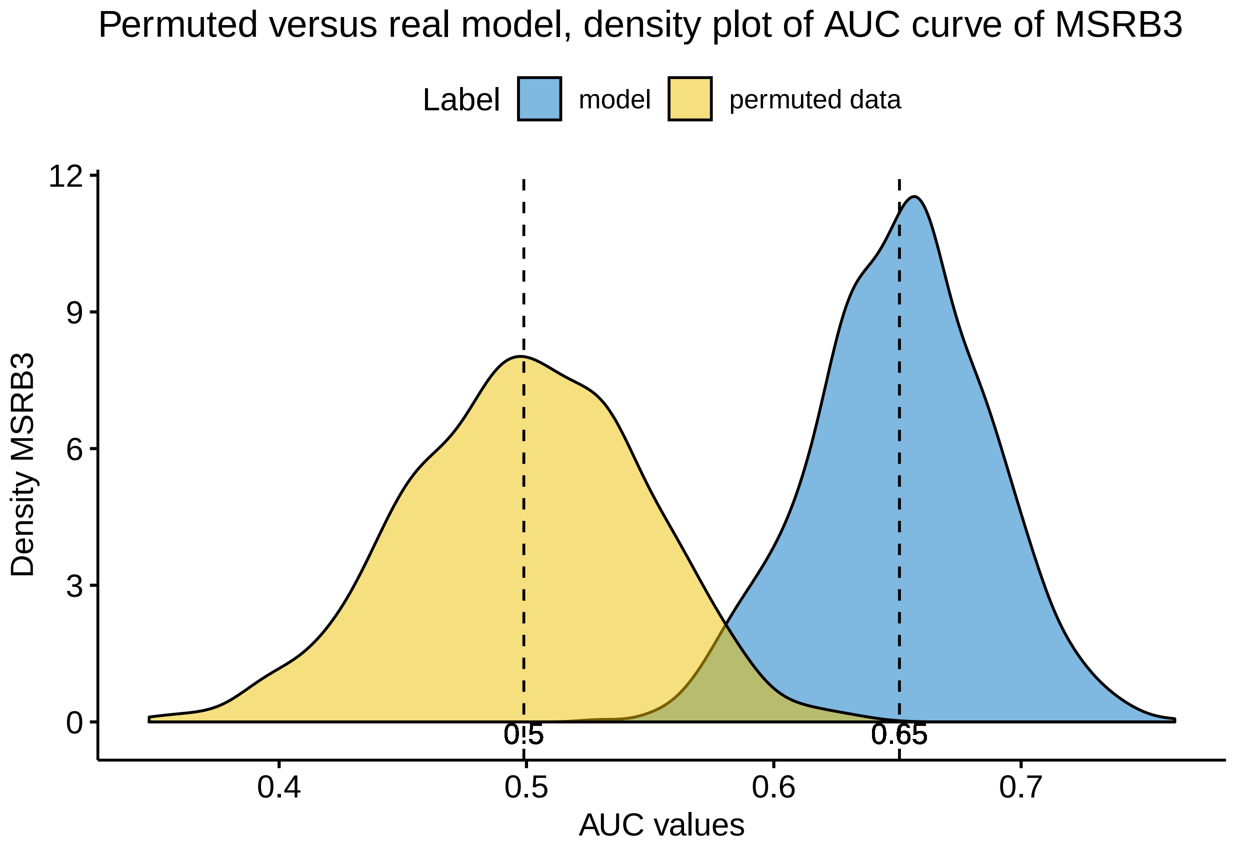

Permuted versus real model, density plot of AUC curve of MSRB3
Label
model
permuted data
Density MSRB3
12
9
6
3
0
0.5
0.65
0.4
0.5
0.6
0.7
AUC values

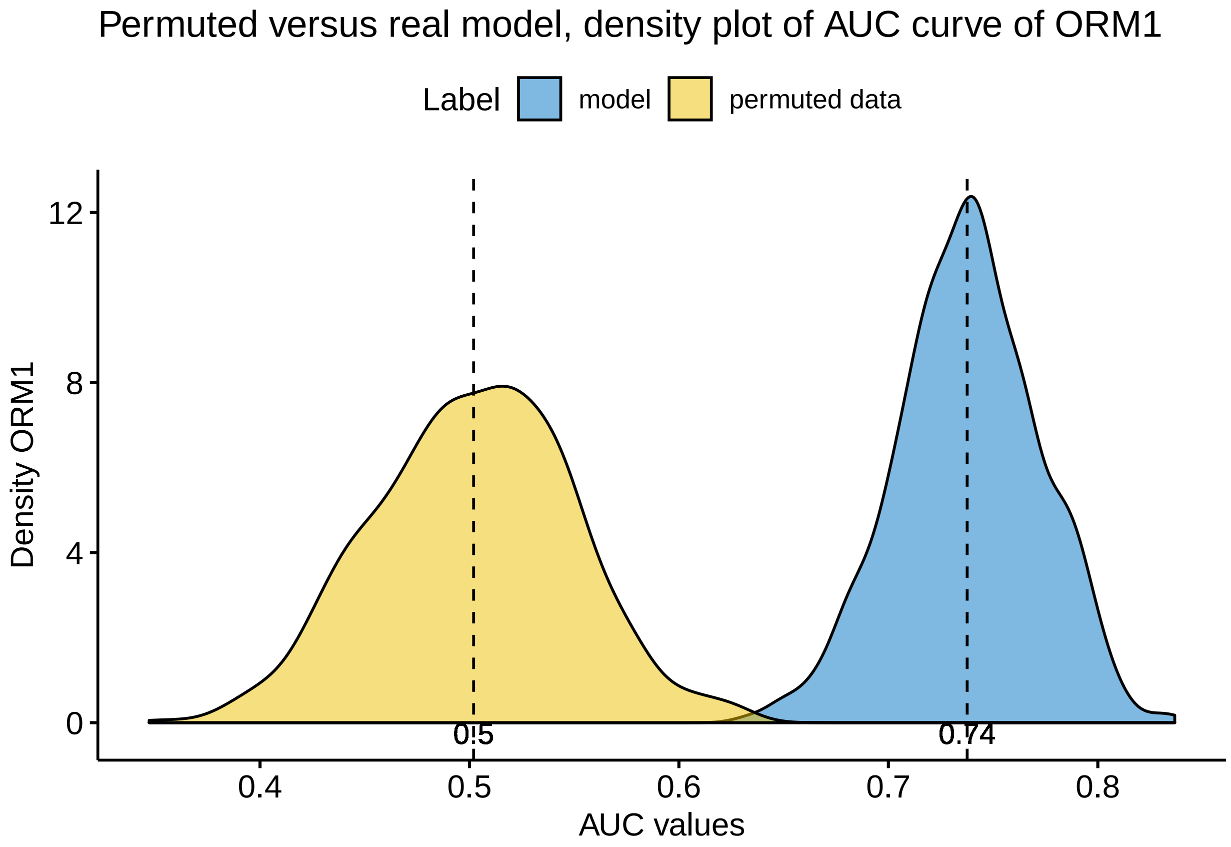

Permuted versus real model, density plot of AUC curve of ORM1
Label
model
permuted data
Density ORM1
12
8
4
0
0.5
0.74
0.4
0.5
0.6
0.7
0.8
AUC values

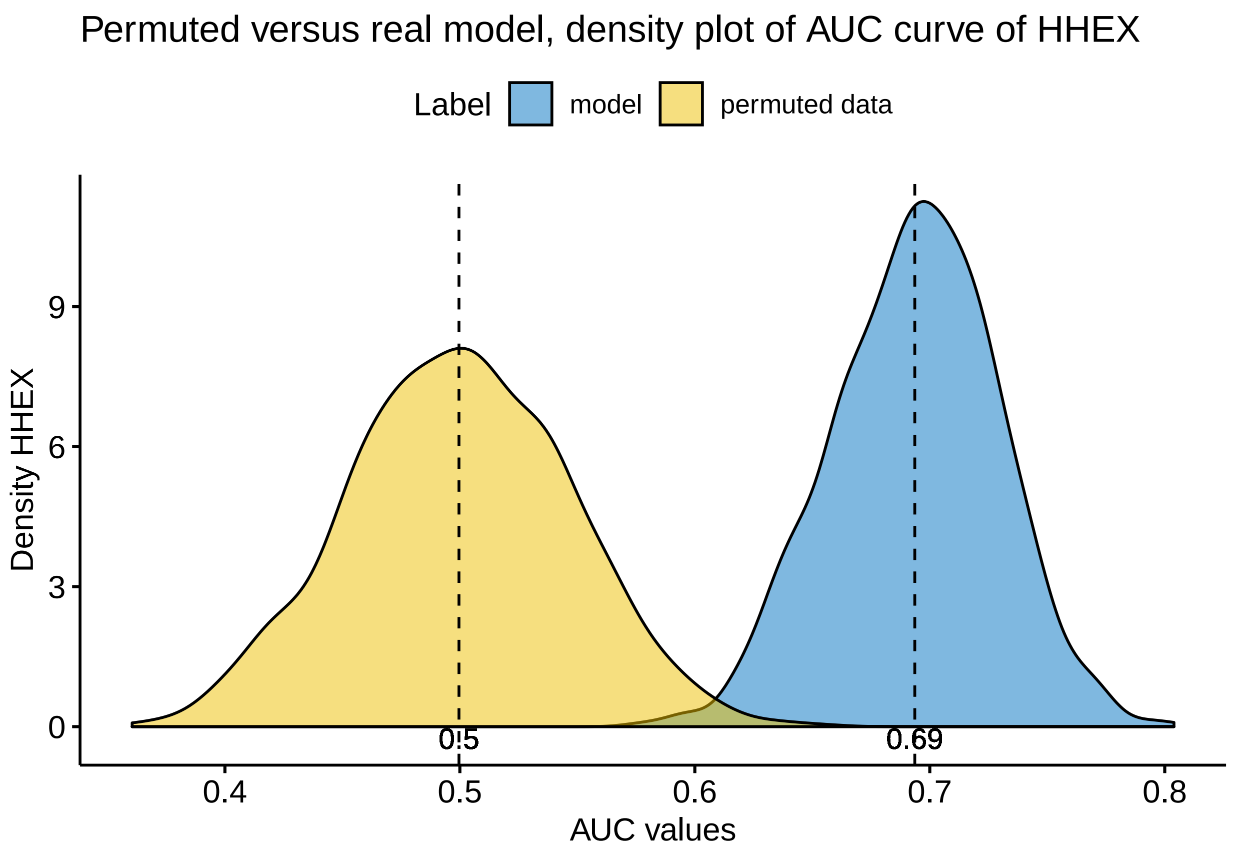
Permuted versus real model, density plot of AUC curve of HHEX
Label
model
permuted data
Density HHEX
9
6
3
0
0.5
0.69
0.4
0.5
0.6
0.7
0.8
AUC values

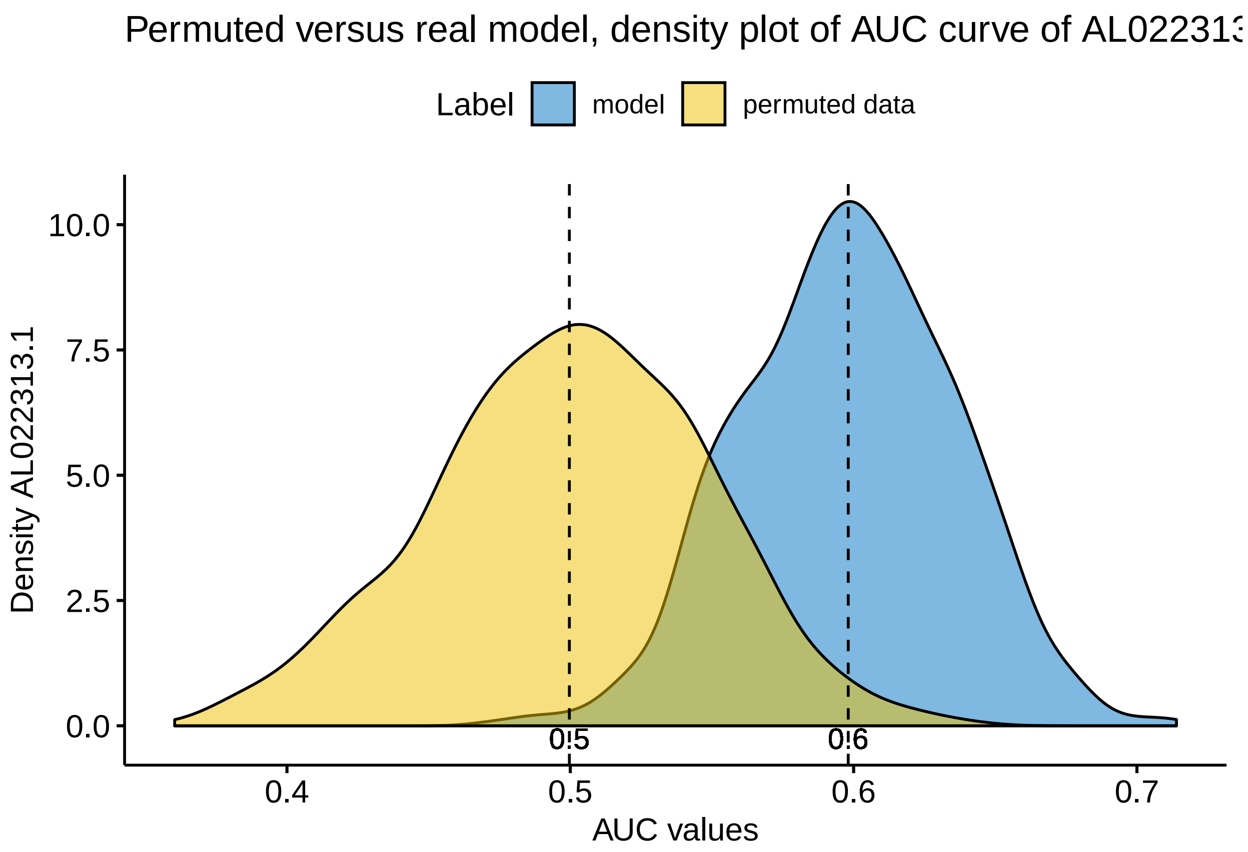

Permuted versus real model, density plot of AUC curve of AL022313
Label
model
permuted data
Density AL022313.1
10.0
7.5
5.0
2.5
0.0
0.5
0.6
0.4
0.5
0.6
0.7
AUC values

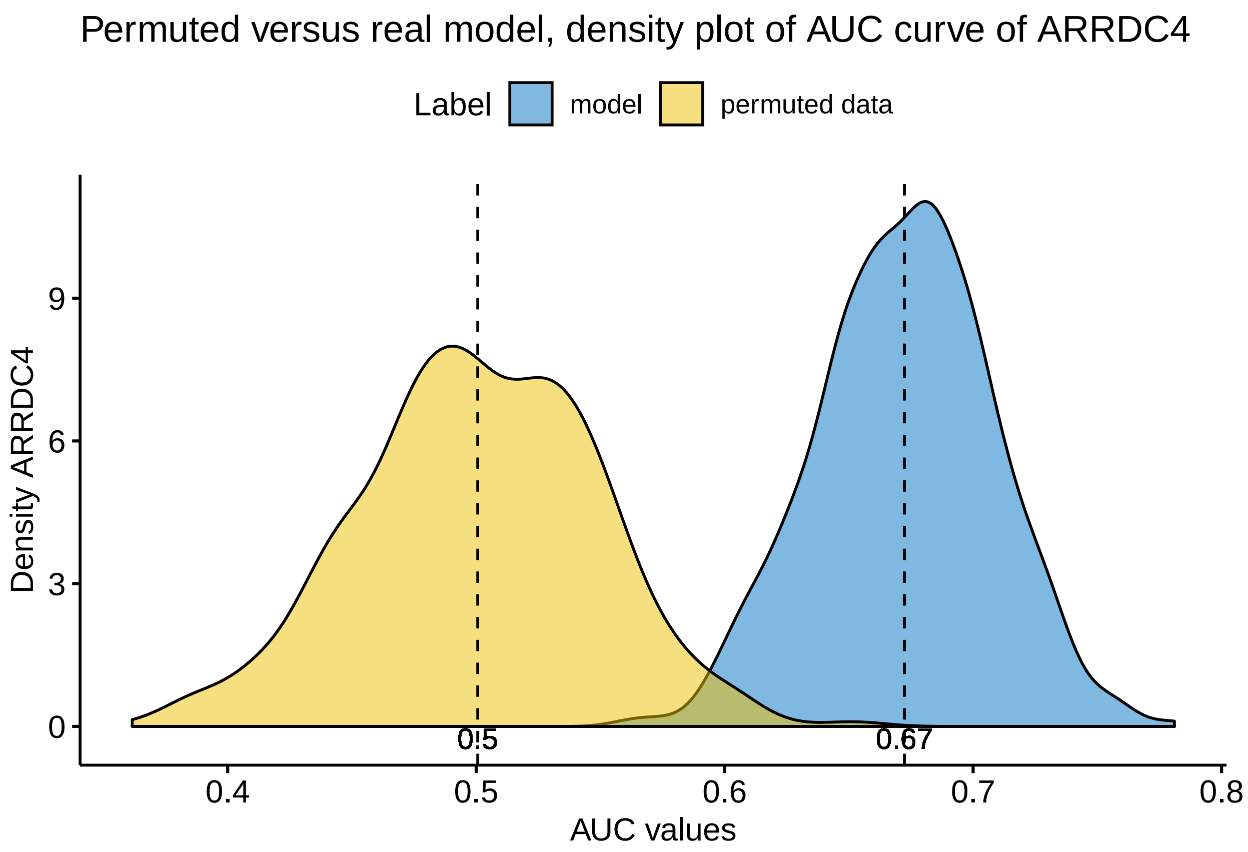

Permuted versus real model, density plot of AUC curve of ARRDC4
Label
model
permuted data
Density ARRDC4
9
6
3
0
0.5
0.67
0.4
0.5
0.6
0.7
0.8
AUC values

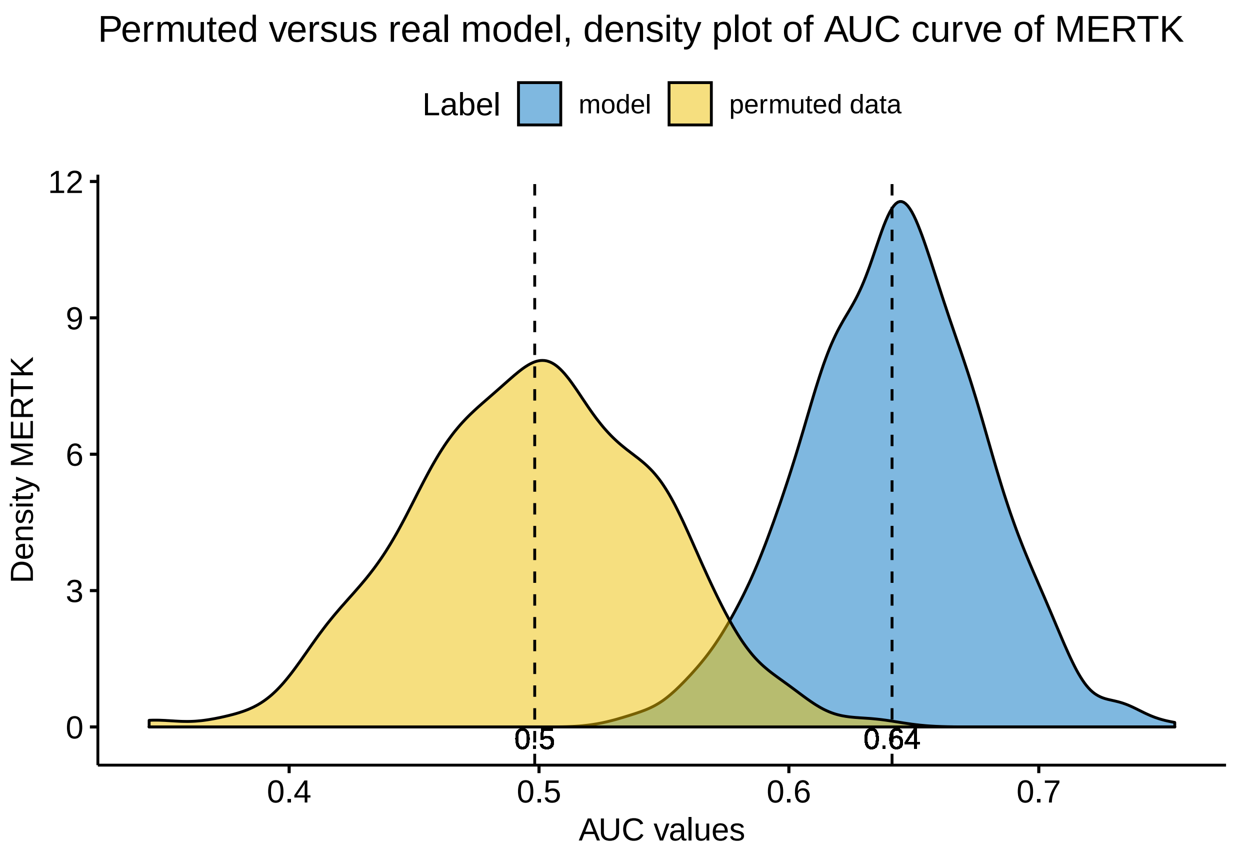
Permuted versus real model, density plot of AUC curve of MERTK
Label
model
permuted data
Density MERTK
12
9
6
3
0
0.5
0.64
0.4
0.5
0.6
0.7
AUC values

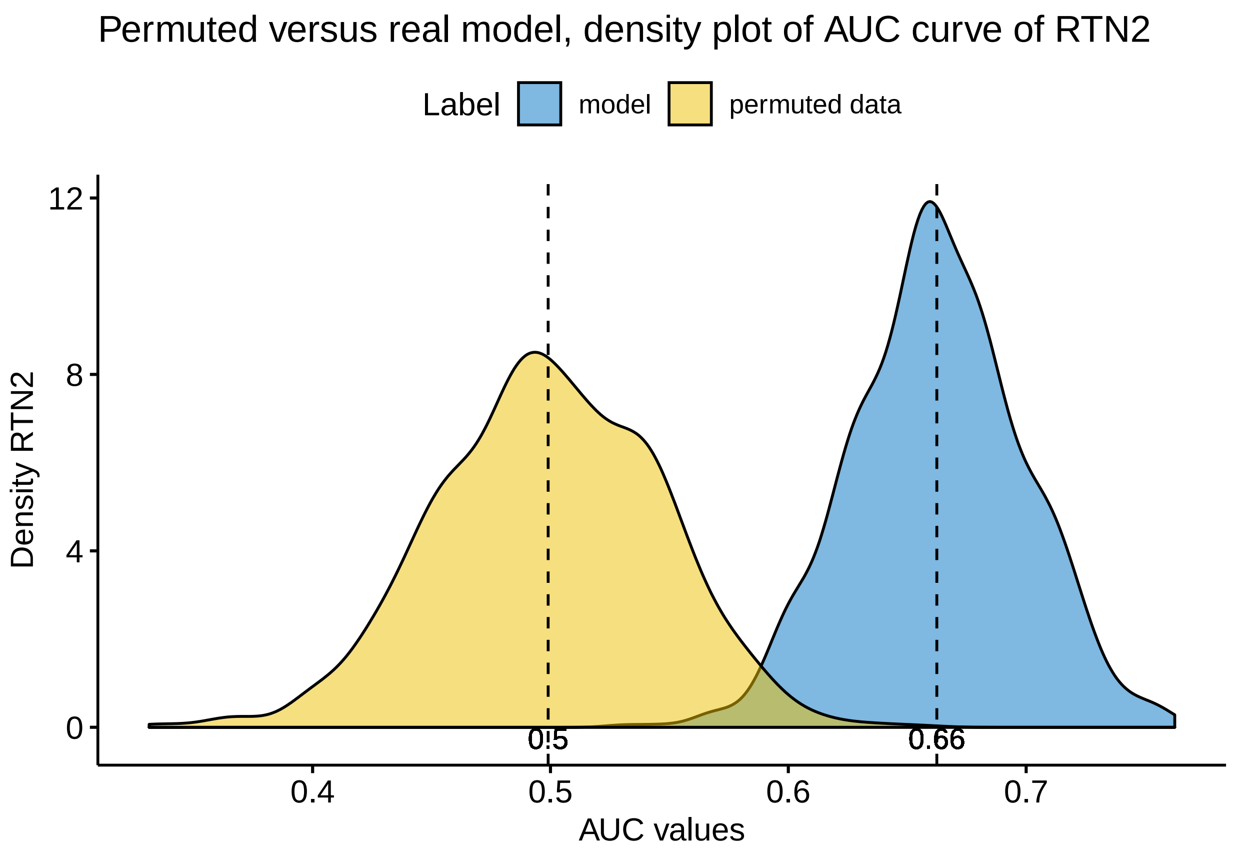


```
# #
n <- length(plot)

# ###Accuracy, Precision etc
#
plot3<-list()
plot4<-list()
Data<-c("Accuracy","Sensitivity","Specificity","Precision")
##### End Block 6
```

```
##### Begin Block 7


#
#Accuracy
Mono<-NULL
MonoR<-NULL
for (s in 1:(NumVar-1)){
  for (d in 1:4){
    for (j in 1:N){
      Mono1<-data.frame(Mono=MatsinglePlus[s,j][[1]][[d]])
      Mono<-rbind(Mono,Mono1)
      Mono1R<-data.frame(MonoR=MatsinglePlusR[s,j][[1]][[d]])
      MonoR<-rbind(MonoR,Mono1R)
    }
    MonoLab<-data.frame(Mono=Mono,Label=as.factor(c(rep("model",dim(Mono)[1]))))
    MonoLabR<-data.frame(Mono=MonoR,Label=as.factor(c(rep("permuted data",dim(MonoR)[1]))))
```

```
    colnames(MonoLabR)[1]<-c("Mono")
    Means<-data.frame(Mean=mean(MonoLab$Mono),MeanR=mean(MonoLabR$Mono))
    Mono<-rbind(MonoLab,MonoLabR)
    plot3[[d]]<-ggdensity(Mono, x = "Mono", fill = "Label", palette = "jco")+labs(title= paste("Value :
    Mono<-NULL
    MonoR<-NULL

  }
  print(grid.arrange(plot3[[2]], plot3[[3]],plot3[[4]],plot3[[1]], ncol=2))

}
```

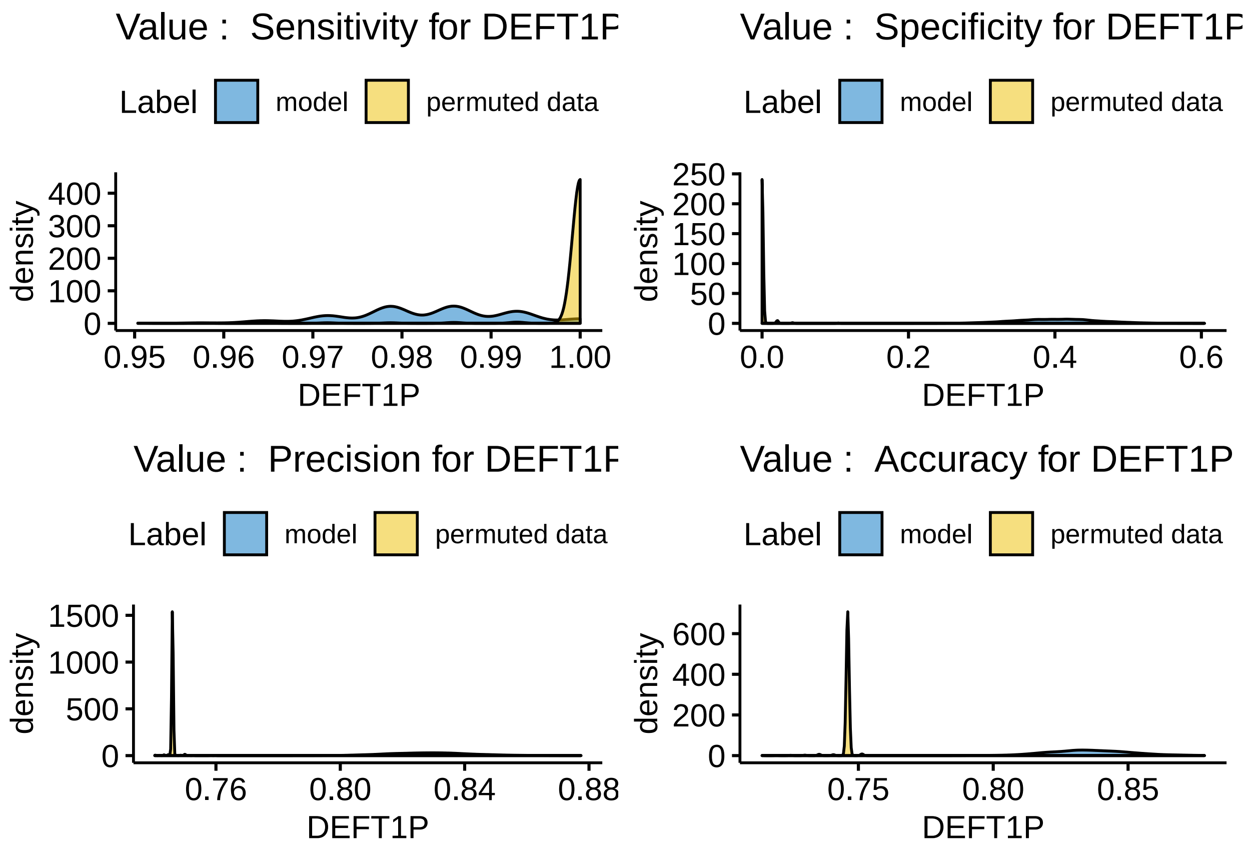


```
## TableGrob (2 x 2) "arrange": 4 grobs
##   z     cells    name           grob
## 1 1 (1-1,1-1) arrange gtable[layout]
## 2 2 (1-1,2-2) arrange gtable[layout]
## 3 3 (2-2,1-1) arrange gtable[layout]
## 4 4 (2-2,2-2) arrange gtable[layout]
```

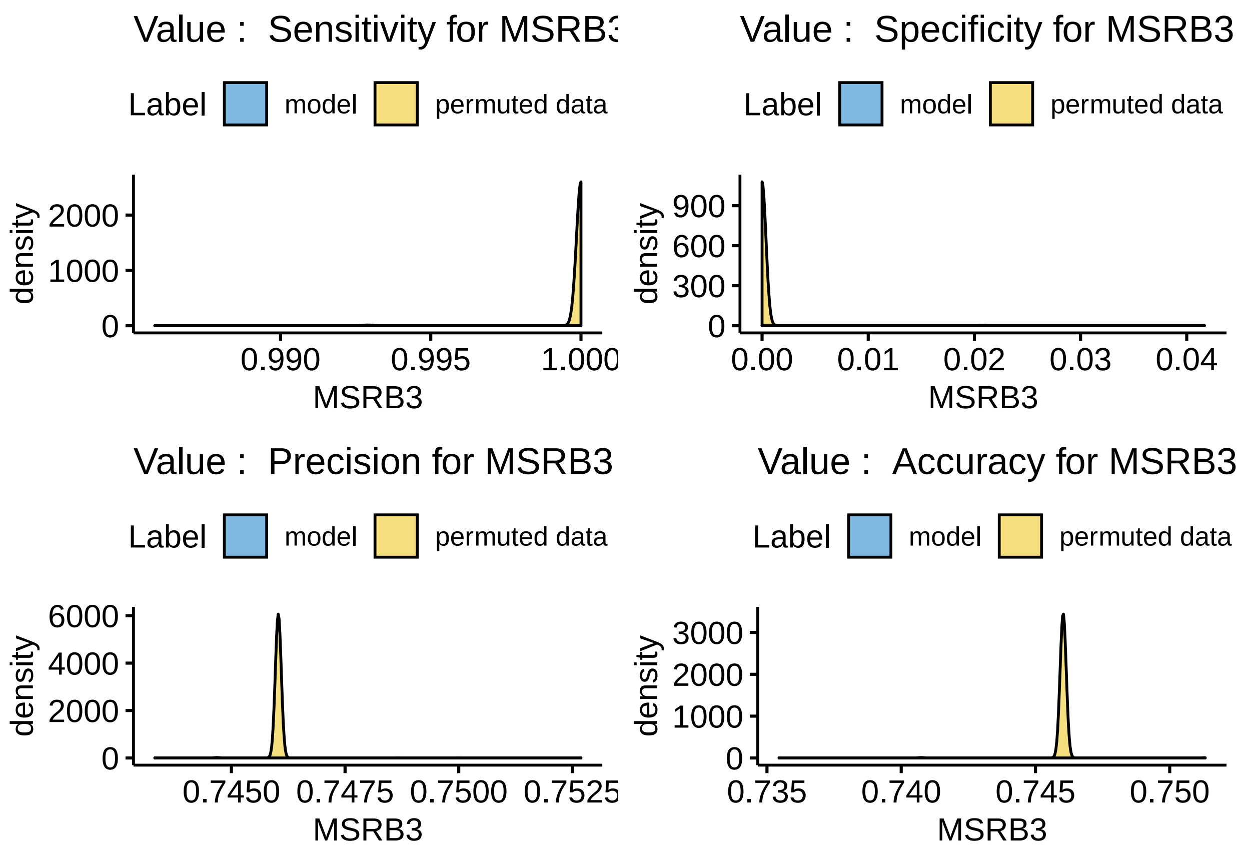


```
## TableGrob (2 x 2) "arrange": 4 grobs
##   z     cells    name           grob
## 1 1 (1-1,1-1) arrange gtable[layout]
## 2 2 (1-1,2-2) arrange gtable[layout]
## 3 3 (2-2,1-1) arrange gtable[layout]
## 4 4 (2-2,2-2) arrange gtable[layout]
```

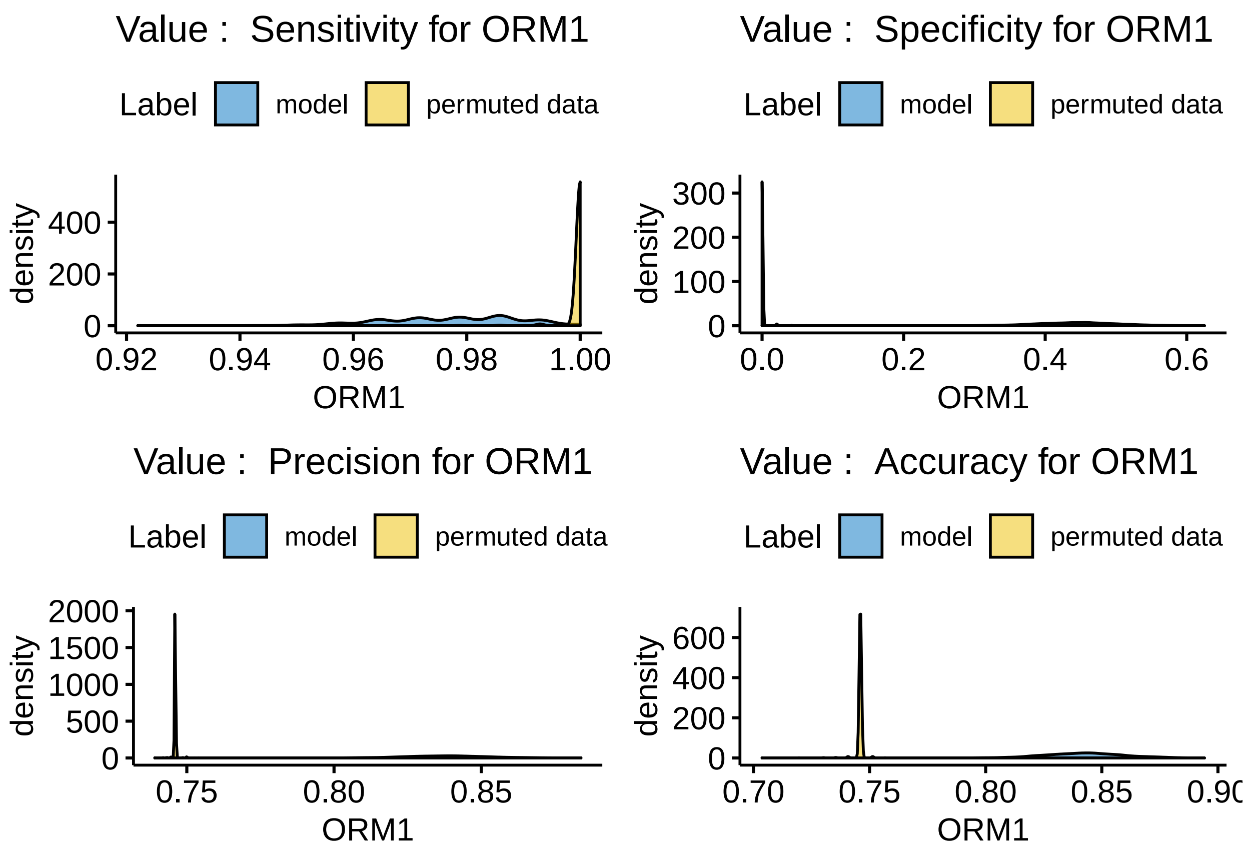


```
## TableGrob (2 x 2) "arrange": 4 grobs
##   z     cells    name           grob
## 1 1 (1-1,1-1) arrange gtable[layout]
## 2 2 (1-1,2-2) arrange gtable[layout]
## 3 3 (2-2,1-1) arrange gtable[layout]
## 4 4 (2-2,2-2) arrange gtable[layout]
```

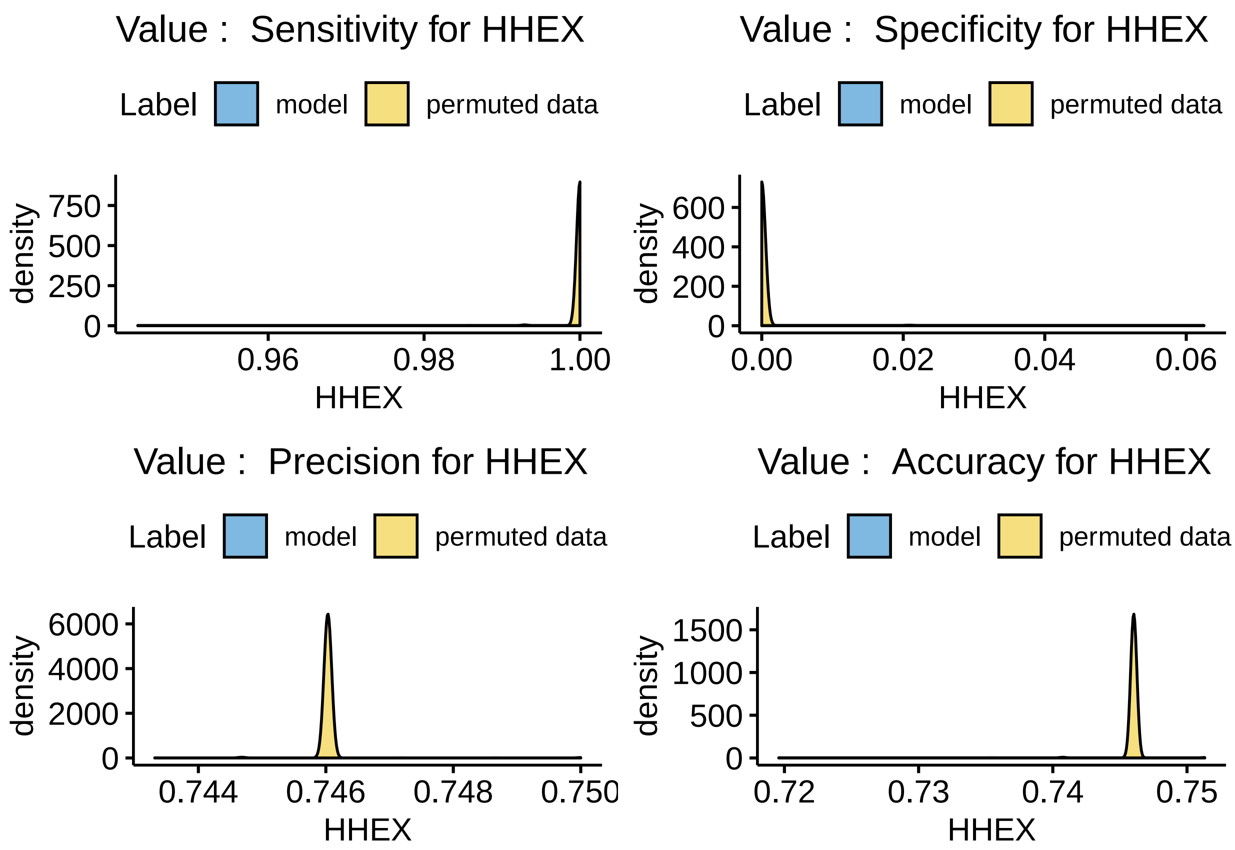


```
## TableGrob (2 x 2) "arrange": 4 grobs
##   z     cells    name           grob
## 1 1 (1-1,1-1) arrange gtable[layout]
## 2 2 (1-1,2-2) arrange gtable[layout]
## 3 3 (2-2,1-1) arrange gtable[layout]
## 4 4 (2-2,2-2) arrange gtable[layout]
```

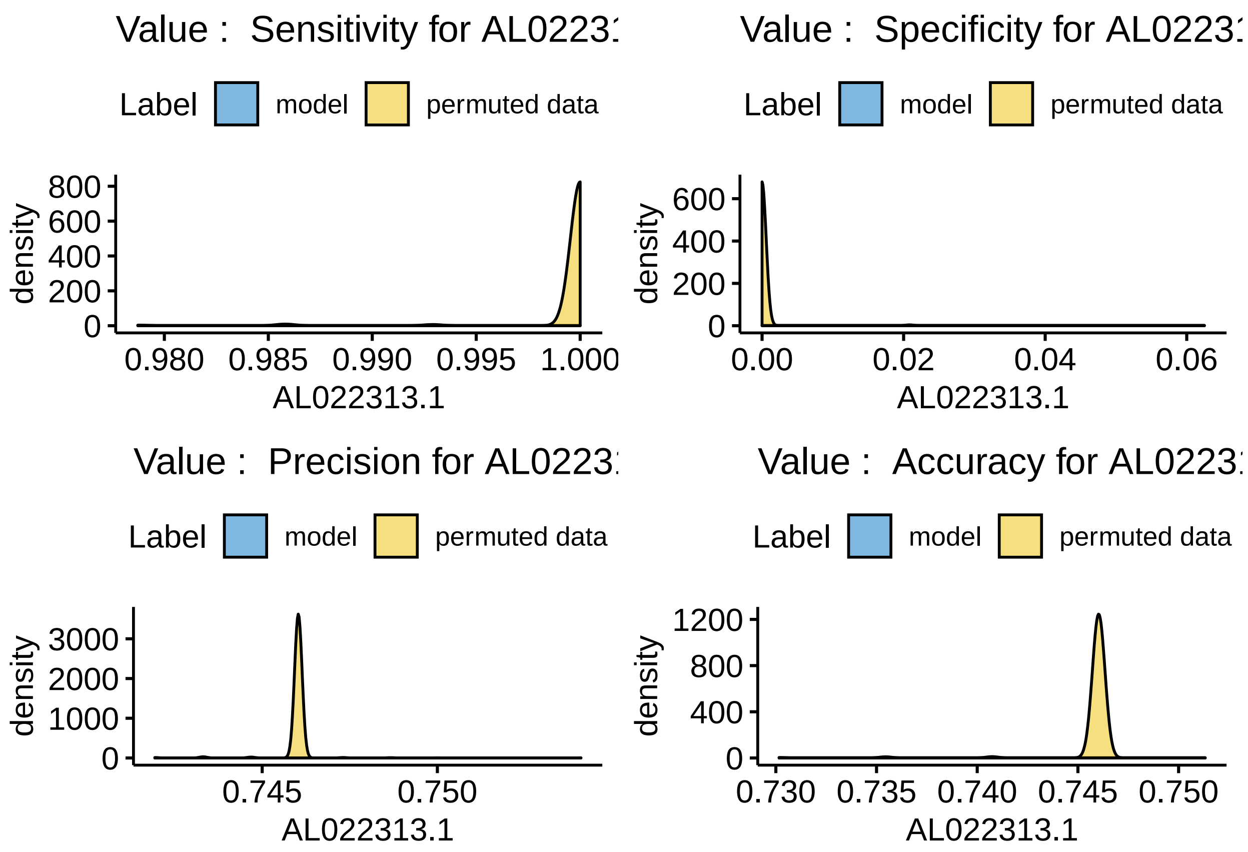


```
## TableGrob (2 x 2) "arrange": 4 grobs
##   z     cells    name           grob
## 1 1 (1-1,1-1) arrange gtable[layout]
## 2 2 (1-1,2-2) arrange gtable[layout]
## 3 3 (2-2,1-1) arrange gtable[layout]
## 4 4 (2-2,2-2) arrange gtable[layout]
```

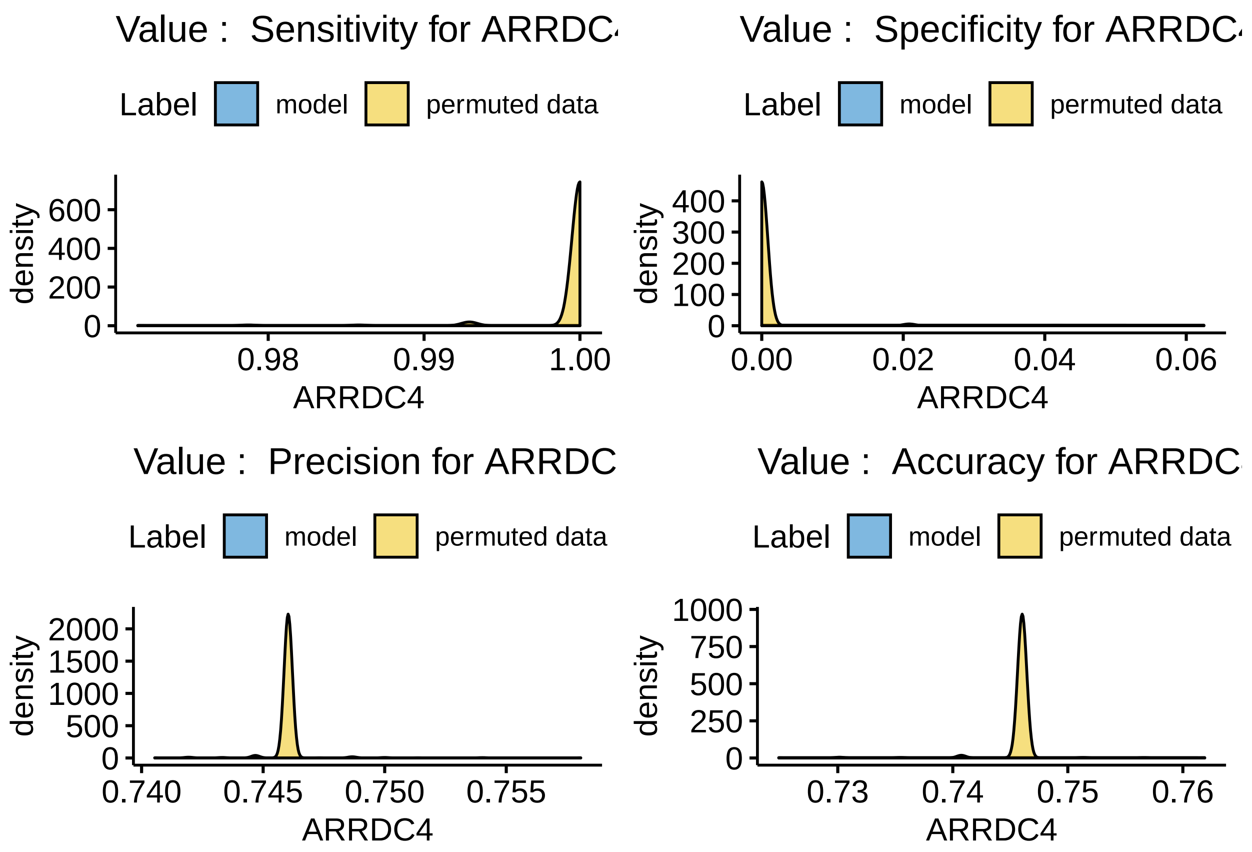


```
## TableGrob (2 x 2) "arrange": 4 grobs
##   z     cells    name           grob
## 1 1 (1-1,1-1) arrange gtable[layout]
## 2 2 (1-1,2-2) arrange gtable[layout]
## 3 3 (2-2,1-1) arrange gtable[layout]
## 4 4 (2-2,2-2) arrange gtable[layout]
```

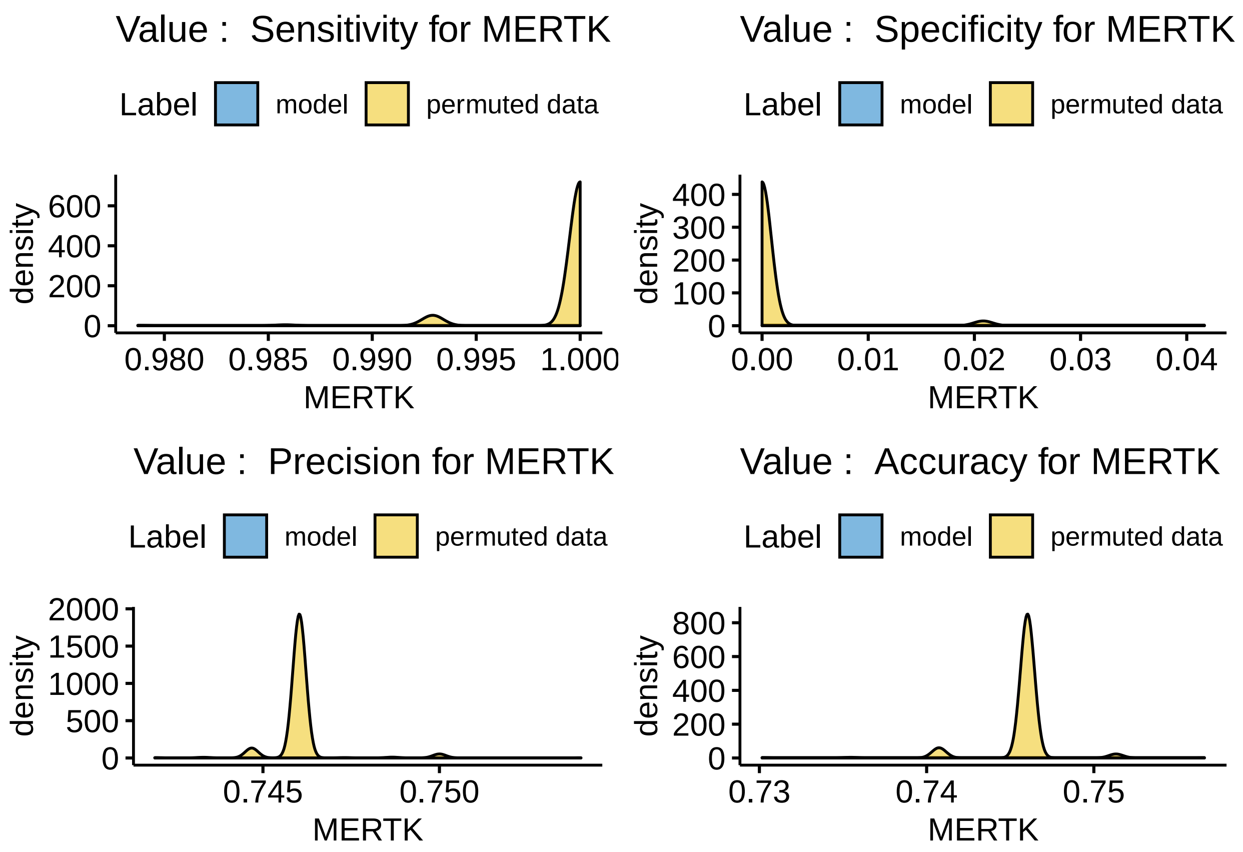


```
## TableGrob (2 x 2) "arrange": 4 grobs
##   z     cells    name           grob
## 1 1 (1-1,1-1) arrange gtable[layout]
## 2 2 (1-1,2-2) arrange gtable[layout]
## 3 3 (2-2,1-1) arrange gtable[layout]
## 4 4 (2-2,2-2) arrange gtable[layout]
```

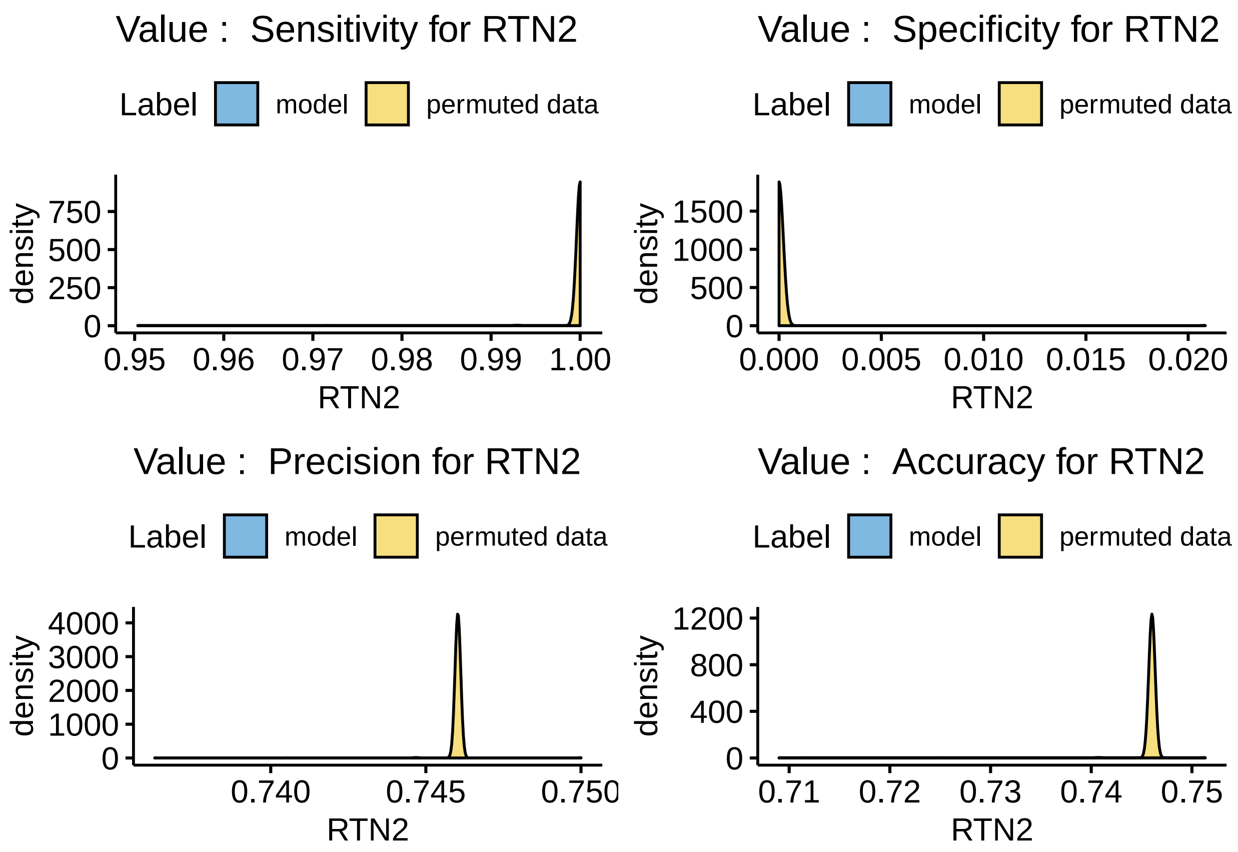


```
## TableGrob (2 x 2) "arrange": 4 grobs
##   z     cells    name           grob
## 1 1 (1-1,1-1) arrange gtable[layout]
## 2 2 (1-1,2-2) arrange gtable[layout]
## 3 3 (2-2,1-1) arrange gtable[layout]
## 4 4 (2-2,2-2) arrange gtable[layout]
```

```
#

Meanq<-data.frame(multipleAUC$Means,multipleAUCR$Means)
MA<-data.frame(Mono=t(multipleAUC[1:dim(multipleAUC)[2]-1]))
MA["Label"]<-as.factor(c(rep("model",dim(MA)[1])))
MAR<-data.frame(Mono=t(multipleAUCR[1:dim(multipleAUCR)[2]-1]))
MAR["Label"]<-as.factor(c(rep("permuted data",dim(MAR)[1])))
Mono<-rbind(MA,MAR)
pp<-ggdensity(Mono, x = "Mono", fill = "Label", palette = "jco")+geom_vline(xintercept=Meanq[1,1],linet
print(pp+geom_vline(xintercept=Meanq[1,2],linetype = 2,color="black",show.legend = TRUE))
```

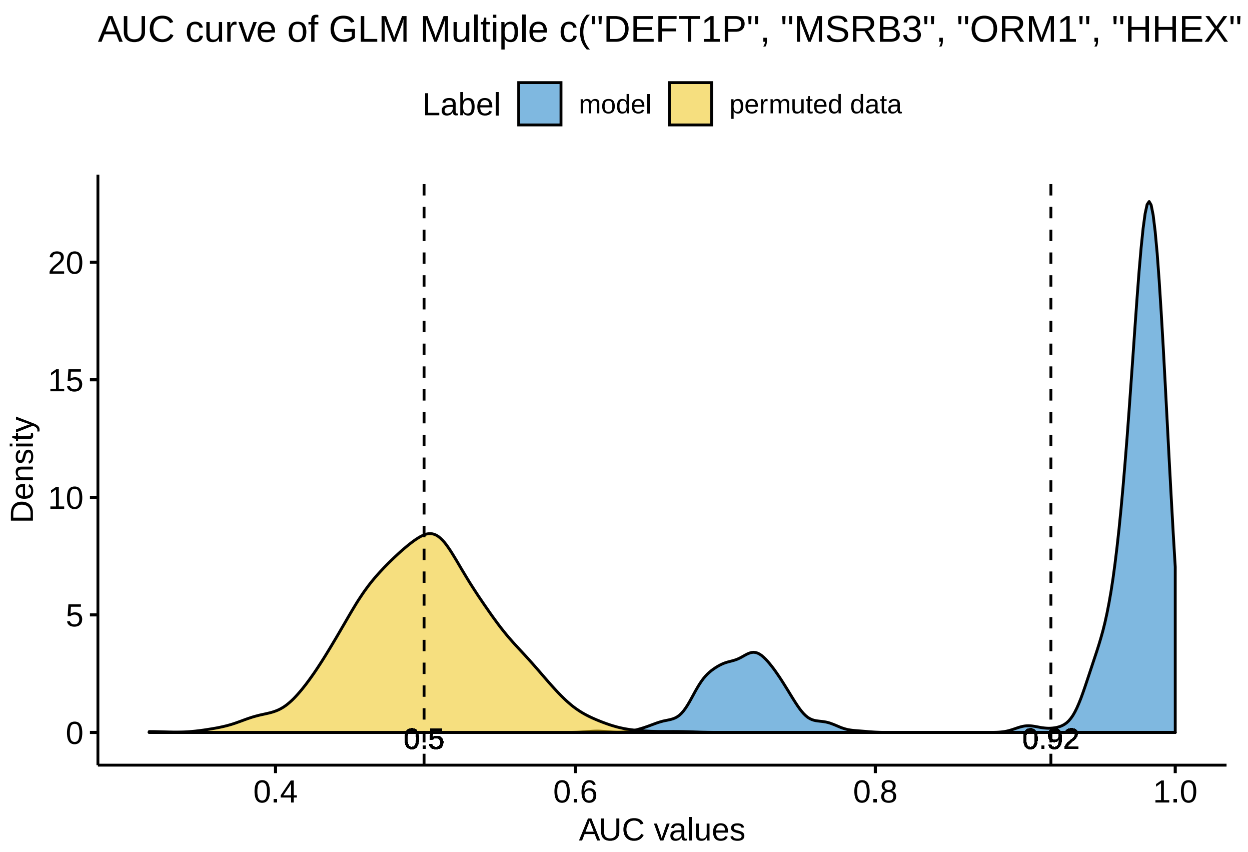


```
Meanq<-data.frame(multipleAUCNB$Means,multipleAUCNBR$Means)
MA<-data.frame(Mono=t(multipleAUCNB[1:dim(multipleAUCNB)[2]-1]))
MA["Label"]<-as.factor(c(rep("model",dim(MA)[1])))
MAR<-data.frame(Mono=t(multipleAUCNBR[1:dim(multipleAUCNBR)[2]-1]))
MAR["Label"]<-as.factor(c(rep("permuted data",dim(MAR)[1])))
Mono<-rbind(MA,MAR)
pp<-ggdensity(Mono, x = "Mono", fill = "Label", palette = "jco")+geom_vline(xintercept=Meanq[1,1],linety
print(pp+geom_vline(xintercept=Meanq[1,2],linetype = 2,color="black",show.legend = TRUE))
```

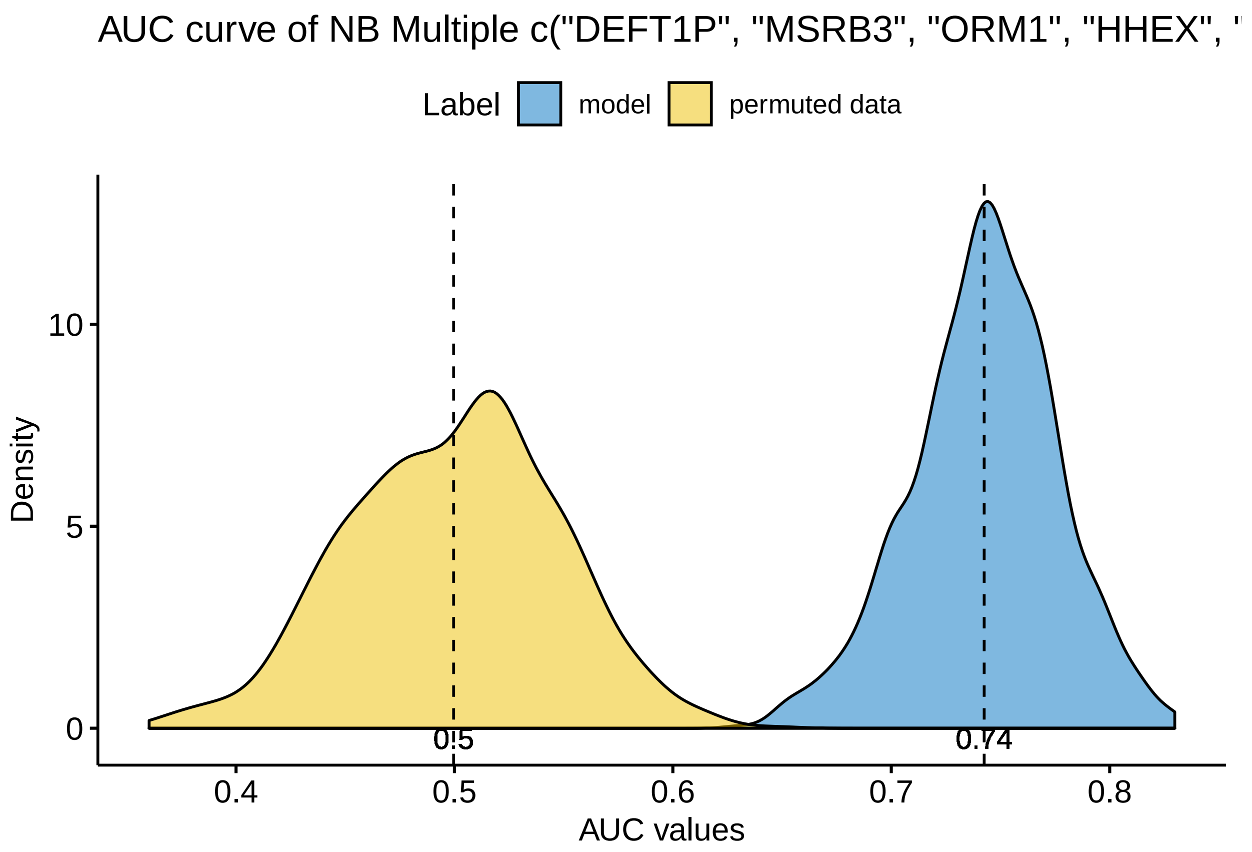


```
ee<-regex(names,".")

Final<-data.frame(MultiNB=t(multipleAUCNB),MultiNBRand=t(multipleAUCNBR),Multi=t(multipleAUC),MultiRand=
FinalMeans<-data.frame(MultiNB=multipleAUCNB$Means,MultiNBRand=multipleAUCNBR$Means,Multi=multipleAUC$Me

##### End Block 7



genes_to_network <- colnames(Betas_select)[-length(colnames(Betas_select))]
genes_to_networkStrict <- colnames(Betas_select2)[-length(colnames(Betas_select2))]


sink("genes_to_network.txt")
cat("Beta_Select_Intial")
```

```
## Beta_Select_Intial
```

```
cat("\n")
```

```
cat(genes_to_network, sep = "\n")
```

```
## LCN2
## CRISP3
## CAMP
## HHEX
## HOXA9
```

```
## KLF10
## SOX4
## BMP1
## TRIO
## SOCS5
## MAP3K7CL
## MAPK12
## DEFT1P
## CD8B2
## FSCN1
## DNAH10
## CYP4F3
## HOXA10
## EEF1A1
## C6orf48
## PNKD
## SLC17A9
## ORM1
## MARK4
## MMP9
## ASPH
## GAS5
## OLFM4
## VPREB3
## IL7R
## NETO2
## C5
## UBE2E2
## TUBB1
## MSRB3
## ORM2
## SNX33
## CFD
## RAB37
## RTN2
## ZNF469
## FLT3
## RPL22
## FBXL19
## IGF2BP2
## ERCC1
## CD160
## AL022313.1
## CD8B
## SLC25A21
## ITPR1
## MERTK
## ALOX12
## ARRDC4
```

```
cat("Beta_Select_Threshold")
```

```
## Beta_Select_Threshold
```

```r
cat("\n")
```

```r
cat(genes_to_networkStrict, sep = "\n")
```

```
## DEFT1P
## MSRB3
## ORM1
## HHEX
## AL022313.1
## ARRDC4
## MERTK
## RTN2
```

```r
sink()
```
